# Using AMOEBA to Uncover Deformation and Hydration Anisotropy in Collagen-Mimetic Peptides

**DOI:** 10.1021/acs.jpcb.6c02718

**Published:** 2026-09-04

**Authors:** Ronnie Mondal, Valerie Vaissier Welborn

**Affiliations:** † Department of Chemistry, 1757 Virginia Tech, Blacksburg, Virginia 24061, United States; ‡ Macromolecules Innovation Institute, Virginia Tech, Blacksburg, Virginia 24061, United States

## Abstract

Collagen is the primary constituent of the supramolecular fibers found in connective tissues. To be able to predict collagen’s properties, we need rigorous characterization at the molecular scale. In this work, we focus on collagen-mimetic peptides (CMPs) that are composed of short sequences of (PPG) tripeptides folded into a triple helix. We use molecular dynamics simulations with the AMOEBA polarizable force field to model these CMPs at physiological temperature. We show that AMOEBA captures length-dependent fraying of the triple helix, consistent with experimental observations. We also introduce new metrics to quantify the deformation of the triple helix and hydration dynamics. This enables a systematic quantification of CMPs under a variety of conditions, which was missing in the field. We apply our approach to mutated CMPs and CMPs in the presence of d-glucose. We find that translational diffusion anisotropy is a molecular signature of CMP structural integrity.

## Graphical abstract

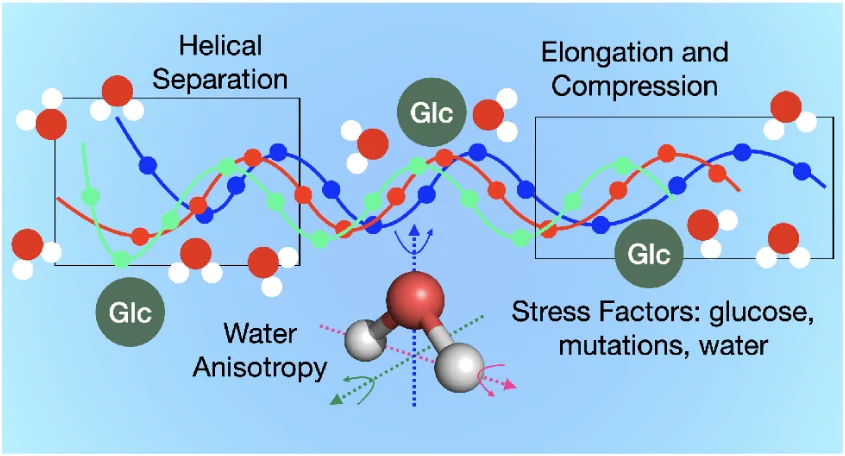

## Introduction

Collagen is the most abundant structural protein in mammals, forming the bulk of connective tissues such as tendons, ligaments, skin, and bone.^1–4^ Collagen is essential for tissue integrity and contributes to the remarkable mechanical properties of biological materials.^1,4–6^ As a result, understanding and controlling collagen structure in various environments is key to many research fields, including biology and tissue engineering.^7–9^

At the molecular level, the structural unit of collagen is a triple helix. The triple helix is a right-handed superhelix that consists of three left-handed polyproline II polypeptide chains, or strands.^1,4,10–12^ Multiple triple helices can then further assemble into protofibrils, fibrils, and fibers (Figure 1).

**Figure 1:**
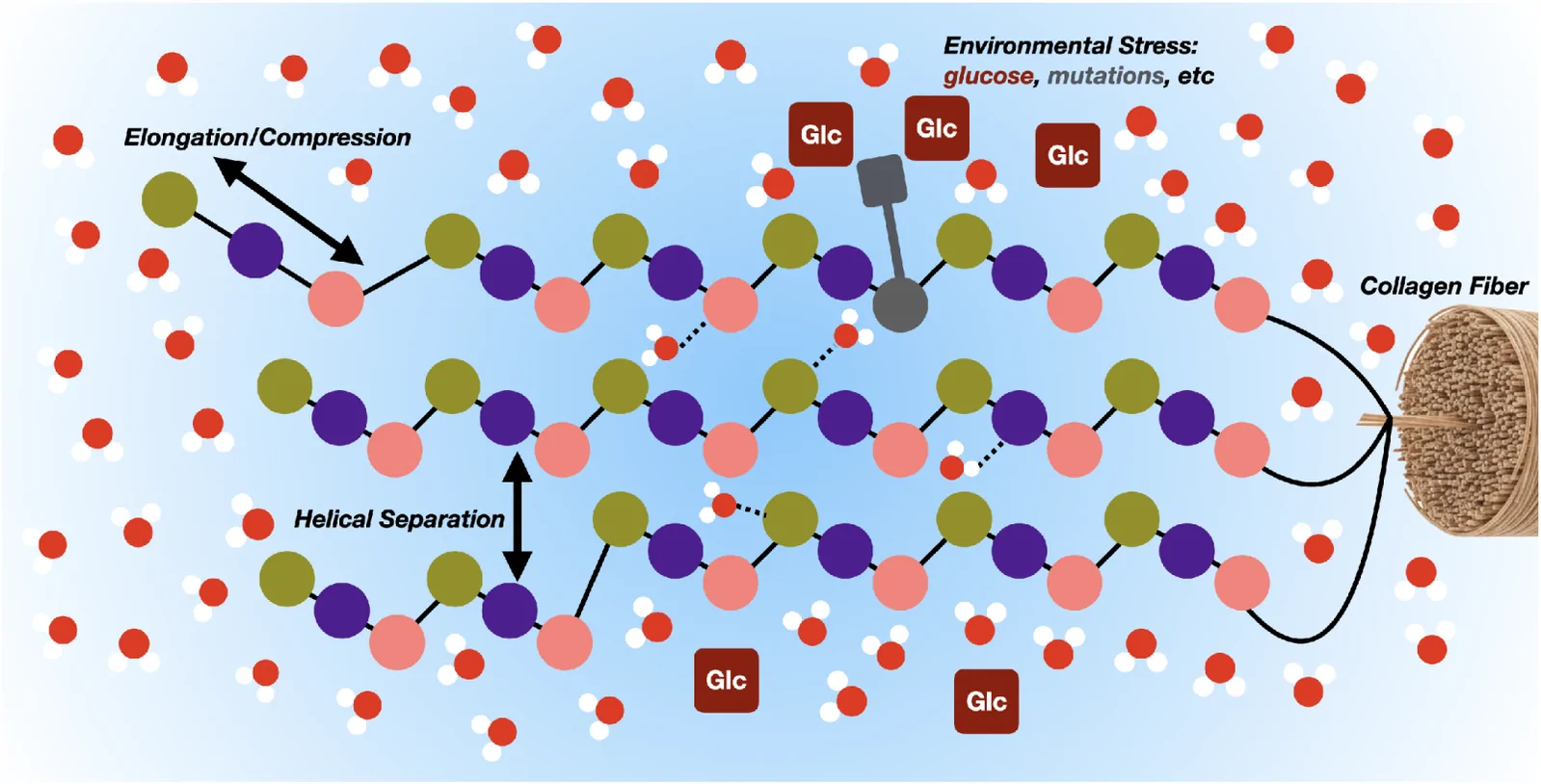
The collagen triple helix is the molecular unit of collagen fibers. Under environmental stress factors like temperature, modified residues, or the presence of reducing sugars, the triple helix can deform along (elongation/compression) or perpendicular to (helical separation) the primary helical axis.

*In vivo*, the collagen triple helix contains over a thousand amino acids per strand, reaching over 300 nm in length.^1,11,13^ Therefore, simplified model systems are often required to study collagen at the molecular scale. Collagen-mimetic peptides (CMPs), including homotrimeric peptides based on repeating (Pro–Pro–Gly)_*n*_ motifs or (PPG)_*n*_, have been widely used as minimal models of the collagen triple helix.^14–16^ These systems reproduce key geometric features of the triple-helical fold while reducing the sequence heterogeneity, length, and hierarchical complexity of native collagen. Native collagen sequences occur primarily as Xaa–Yaa–Gly triplets, where Gly appears every third position and Xaa and Yaa are frequently, but not exclusively, occupied by Pro or Hyp residues.^1,4,14,17^ In this context, idealized (PPG)_*n*_ peptides are best understood as simplified collagen-like systems: their regular sequence composition, including higher proline content than native collagen, enables controlled studies of triple-helix structure and stability, while necessarily abstracting away from the full chemical diversity and biological complexity of collagen *in vivo*. This balance between structural fidelity and molecular simplicity makes CMPs useful, fundamental platforms for isolating factors that influence triple-helix structure and stability, including hydrogen bonding, hydration, chain packing, and local conformational responses to chemical or sequence perturbations.^14–16^ The modular design of CMPs further enables the systematic introduction of mutations, providing a controlled means to examine how local variations in residue identity influence collagen-like triple-helix structure and stability.^18–20^

Experimental and crystallographic studies of (PPG)_*n*_ CMPs have provided important details about the structure of the triple helix, including backbone dihedral angles,^21–23^ pyrrolidine ring conformations,^23–25^ and interstrand hydrogen bonding.^22,24,25^ For example, deformation studies, both in the presence and absence of a mechanical force, revealed that the melting point of (PPG)_*n*_ triple helices depends strongly on chain length, with helices with *n* ≤ 12 exhibiting melting temperatures below the physiological temperature (310 K).^26–31^ Deformation of the triple helix has also been observed to correlate with the disruption of interstrand HBs by water molecules.^1,12,32,33^ Overall, the community agrees on the presence of one HB per tripeptide, established between the Gly­(NH) group of one strand and the Xaa­(CO) group of another.^21–23^ However, stabilizing HBs with water were also reported, between 3 and 4 per tripeptide, established between water and the backbone NH and CO groups. Other studies described water molecules forming bridges across strands (e.g., collagen-water-water-collagen HBs), which could further contribute to triple-helix stability. However, molecular dynamics (MD) simulations were not able to confirm the existence of such bridges.^27,34,35^

Waters involved in HB are a subset of the 7–15 water molecules per tripeptide that form the ordered hydration shell of (PPG)_*n*_ CMPs.^1,23,36^ The characteristic residence times of these water molecules fall within the subnanosecond range.^37–39^ However, CMP hydration studies thus far have primarily focused on quantifying hydrogen-bonding patterns and hydration numbers, providing limited information on hydration dynamics, particularly with respect to the anisotropy of (PPG)_*n*_ triple helices.

Computationally, most MD studies of CMPs have relied on force fields such as CHARMM^27,34,40–44^ or AMBER.^7,45–48^ For example, CHARMM36 + TIP3P (C3P) MD simulations reproduced aspects of (PPG)_*n*_ CMP structure, such as backbone and side-chain dihedral angles, at both the molecular (≤12 tripeptides) and submicron scales (∼85 nm).^44,49^ However, the short (PPG)_*n*_ CMPs modeled retained their triple-helical structure at 310 K, which contradicts the aforementioned deformation studies.^10,50–52^ More recently, Pantelopulos et al. introduced a correction for CHARMM36,^53^ called CHARMM36mGP,^54^ to improve the predictability of CMP crystal structures. Overall, they report that AMBER + TIP4P (A4P) and CHARMM36mGP + TIP4P (Cm4P) can best model CMP crystal structures, although they did not assess the deformation of short CMPs.^54^ The limitations of CHARMM36, CHARMM36mGP, and AMBER in characterizing the structure and dynamics of CMPs could be due to the lack of explicit polarization and/or a mistreatment of van der Waals interactions. Indeed, traditional force fields often overestimate protein–protein interactions over protein–water interactions.^55–58^ The balance of these interactions has proven critical for modeling globular and intrinsically disordered proteins,^59–61^ as well as carbohydrates,^62–64^ and we expect it to be critical for CMPs. Another CMP characteristic that could be mistreated with nonpolarizable force fields is noncovalent *n* → π* interactions between adjacent carbonyl groups.^65–67^ These interactions have been shown in density functional theory calculations to compete with interstrand hydrogen bonding,^68,69^ potentially disrupting triple-helix stability.

In this work, we present MD simulations of (PPG)_*n*_ CMPs (*n* = 5, 12, 25) with the AMOEBA polarizable force field.^58,70^ We first validate the performance of AMOEBA compared to C3P, CHARMM36+TIP4P (C4P), Cm4P, A4P, and expectations from prior experimental and computational work. We analyze hydration structure and dynamics around CMPs, uncovering directional dependencies linked to triple-helix deformation. Finally, we examine the effects of local sequence and chemical perturbations in (PPG)_*n*_ triple helices, focusing on Gly → Ala and Pro → Lys substitutions in the presence of d-glucose. These perturbations provide controlled model systems for probing how changes in residue identity, side-chain chemistry, and local sugar interactions can influence collagen-like triple-helix structure. Gly substitutions are of broad interest because Gly is required at every third position in collagen triple helices, and replacement by larger residues is known to disrupt triple-helix stability.^20,71^ Lys-containing sites are also relevant to collagen chemistry because Lys side chains contain reactive amine groups that can participate in nonenzymatic glycation reactions with reducing sugars, such as d-glucose, forming Schiff bases and subsequent Amadori products.^72–74^ To this end, we develop new metrics to systematically quantify the deformation of (PPG)_*n*_ triple helices.

## Methods

### System Preparation

CMPs were generated using the THeBuScr: Interactive triple-helical collagen building script.^75^ THeBuScr constructs triple helices using statistical conformational parameters derived from high-resolution experimental structures of collagen-like peptides. Its use to generate starting structures for CMP simulations is well established in the literature.^76–80^ Three lengths were built: (PPG)_5_, (PPG)_12_, and (PPG)_25_. Each model contained three strands in a canonical triple-helical conformation. We note that the deformation metrics presented in this paper are evaluated relative to these initial structures and therefore retain some dependence on the chosen starting geometry. In Figure S1, we show the structural overlap between our THeBuScr-generated (PPG)_12_ model and the experimentally determined (PPG)_10_ structure.^17^ The corresponding backbone and side-chain dihedral angles for both structures are reported in Table S1. We see that the relevant structural features of the generated model are consistent with those of the experimental (PPG)_10_ structure, supporting the use of idealized THeBuScr-generated conformations as starting configurations for our MD simulations.

Each CMP was then solvated with TIP3P^81^ and TIP4P^82^ water using the solvate command in gromacs.^83,84^ The box dimensions, calculated with packmol,^85^ were set to 55 × 55 × 55 Å^3^ for (PPG)_5_, 50 × 50 × 120 Å^3^ for (PPG)_12_, and 60 × 60 × 275 Å^3^ for (PPG)_25_. We validated the use of rectangular boxes by quantifying possible interactions between the unit cell and its periodic images (Figure S2) and by running a control simulation for (PPG)_12_ in which the linear and angular momenta were removed at each time step (Figure S3). After solvation, d-glucose was added using the gmx insert-molecules command to achieve final concentrations of 0 mM, 500 mM, 1000 mM, and 2000 mM. Solvated CMP coordinate files can be found following the link given in Data Availability.

### Molecular Dynamics Simulations

We started by running 500 ns MD simulations in gromacs in the NPT ensemble (1 atm, 310.15 K, 2 fs time step) using C3P, C4P, Cm4P, and A4P (ff19sb). Temperature and pressure were maintained with the Nosé–Hoover thermostat^86^ and the Parrinello–Rahman barostat,^87^ respectively. A link to the MD input files, prepared via charmm-gui,^88^ is given in Data Availability.

The final snapshots of the C3P simulations were then used as input files for simulations with the polarizable AMOEBA force field.^58^ The C3P simulations preserved the overall geometry of the triple helix, as indicated by RMSD values of 1.98, 2.19, and 3.98 Å between the initial and final structures of (PPG)_5_, (PPG)_12_, and (PPG)_25_, respectively (Figure S4). Therefore, AMOEBA simulations may be initiated from either the THeBuScr structure or the final frame of the C3P simulations. Figures S5–S7 confirm that the results presented in the main text are independent of this choice of starting structure.

The parameters for the protein and water were obtained from the amoebabio18 parameters.^58,89^ The parameters for d-glucose were obtained from previously published work.^64^ Each system was first minimized in tinker9 ^90^ using steepest descent, with a root-mean-square gradient per-atom criterion of 0.01 kcal/mol/Å, before running 100 ns MD in the NPT ensemble (1 atm, 310.15 K, 1 fs time step). Snapshots were saved every 1 ps, and the temperature and pressure were maintained with the Nosé–Hoover thermostat and barostat.^86,91^

(PPG)_12_ mutants were also prepared from the final snapshot of the C3P simulations. Using PyMOL’s mutagenesis wizard,^92^ two Gly → Ala sequences were created, introducing the mutation on each of the three strands. One sequence had the mutation at the sixth tripeptide $\left(\mathrm{Gly}\overset{\mathrm{mid}}{\to }\mathrm{Ala}\right)$ and another had a mutation at the tenth tripeptide $\left(\mathrm{Gly}\overset{\mathrm{end}}{\to }\mathrm{Ala}\right)$ . Similarly, two Pro → Lys mutations, $\mathrm{Pro}\overset{\mathrm{mid}}{\to }\mathrm{Lys}$ and $\mathrm{Pro}\overset{\mathrm{end}}{\to }\mathrm{Lys}$ , were introduced at the sixth and tenth tripeptides at the Yaa position. Note that since the Lys side chain is protonated at physiological pH, three chloride ions were added to the solvated system for charge balance. The link to all AMOEBA MD input structures is given in Data Availability.

Our MD simulations capture short-timescale deformations from a folded starting structure rather than a fully converged equilibrium ensemble, which would be expected to contain both triple helices and monomers, particularly for the shorter CMPs. Monitoring the system’s potential energy components revealed no sudden strains, energy spikes, instabilities, or anomalously strong interactions. The root-mean-square deviations (RMSDs) for all C3P, C4P, Cm4P, A4P, and AMOEBA simulations are presented in Figure S8.

### Computation of Angles, Distances, Hydrogen Bonds, and Radial Distribution Functions

The MD trajectories were analyzed using in-house codes in Tcl for vmd^93^ and Python3 to compute Radial Distribution Functions (RDFs), bonds, angles, dihedrals $\phi \left(C^{i-1}-N^{i}-C_{\alpha }^{i}-C^{i}\right),\psi \left(N^{i}-C_{\alpha }^{i}-C^{i}-N^{i+1}\right),\omega$ , and the $\left(C_{\alpha }^{i-1}-C^{i}-N^{i}-C_{\alpha }^{i}\right)$ pyrrolidine ring puckering angle $\chi (N^{i}-C_{\alpha }^{i}-C_{\beta }^{i}-C_{\gamma }^{i})$ (Figure 2a). We also computed HBs with the following bond and angle cutoffs (Figure 2c): 3.2 Å and 30° for Gly­(NH)-Xaa­(CO) HBs; 3.5 Å and 30° for Xaa­(C_α_)-CO HBs; and 3.2 Å and 30° for CMP-water HBs. A link to all scripts is given in Data Availability.

**Figure 2:**
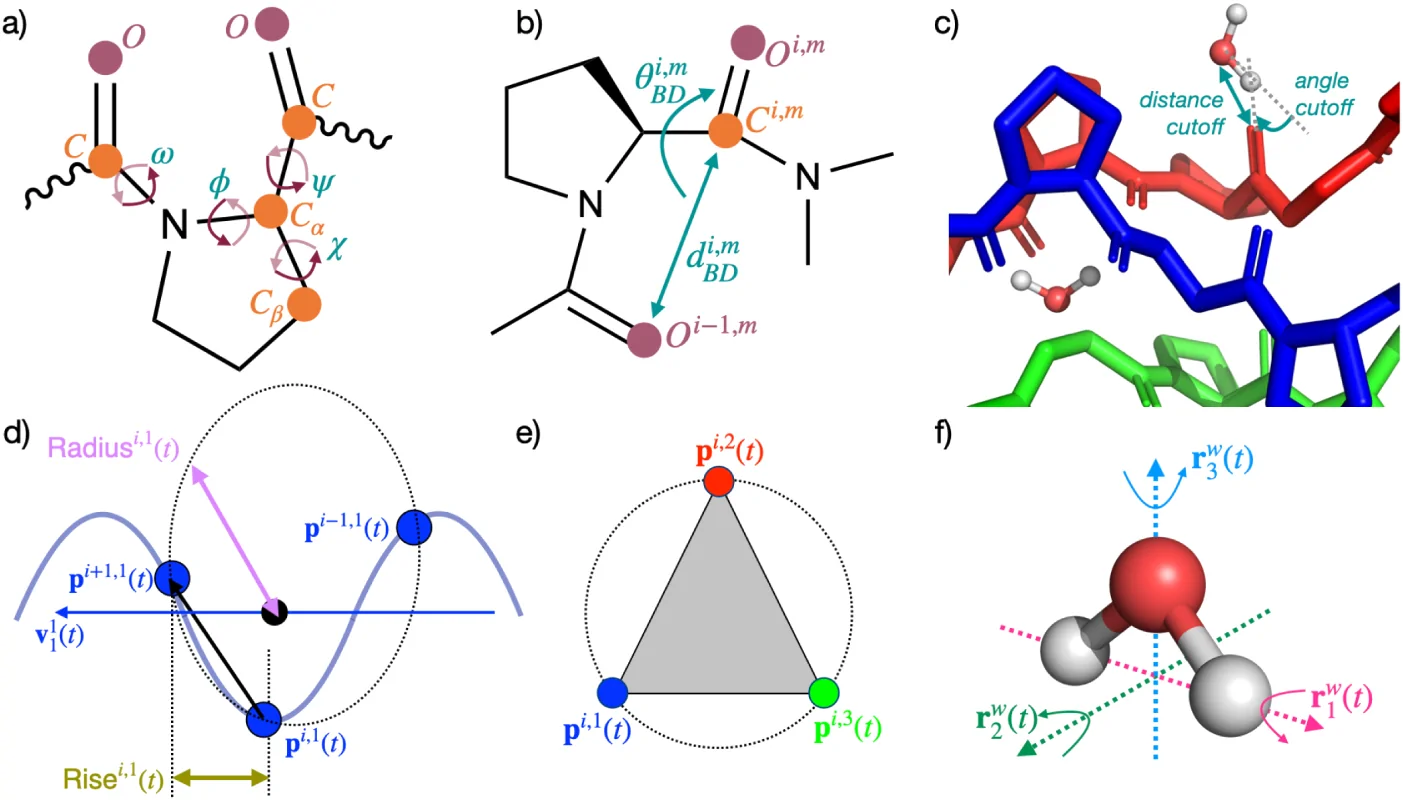
a)­ϕ, ψ, ω, and χ angles with reference to a Pro residue. Only ϕ, ψ, and ω are calculated for Gly. b) Bürgi–Dunitz distance $d_{BD}^{i,m}$ and angle $\theta_{BD}^{i,m}$ on a peptide bond, used to characterize *n* → π* interactions. c) (PPG)*_n_*-water HBs in the system, with distance and angle cutoffs for HBs. d) Rise^*i*,1^(*t*) and Radius^*i*,1^(*t*) of strand 1 (in blue), used to calculate intrastrand deformation. e) Triangle formed by three C_α_ on each strand (in blue, red, and green), used to calculate Area*^i^* (*t*) and Shape*^i^* (*t*) for interstrand deformations. f) $r_{1}^{w}$ , $r_{2}^{w}$ , and $r_{2}^{w}$ in the local frame of a water molecule *w*.

### Quantifying Triple-Helix Deformation

To quantify the deformation of the CMP triple helix, we used our in-house code, HeliXplore. A link to HeliXplore is also provided in the Data Availability.

#### Principal Strand Axes

The principal strand axis, $v_{1}^{m}\left(t\right)$ , is determined for each strand *m* at time *t* via principal component analysis^94–96^ with numpy.linalg. Specifically, we define $v_{1}^{m}\left(t\right)$ , as the principal eigenvector satisfying

1 $$\left[\frac{1}{N}\overset{N}{\underset{i=1}{\sum }}\left(p^{i,m}\left(t\right)-c^{m}\left(t\right)\right){\left(p^{i,m}\left(t\right)-c^{m}\left(t\right)\right)}^{T}\right]v_{1}^{m}\left(t\right)=\lambda_{1}^{m}\left(t\right)v_{1}^{m}\left(t\right)$$

where $\lambda_{1}^{m}\left(t\right)$ is the principal eigenvalue, *N* is the number of residues in strand *m,* and **p** ^*i*,*m*^(*t*) are the C_α_ coordinates of residue *i* on strand *m* at time *t*. **c** ^*m*^(*t*) is the average C_α_ coordinates on strand *m* at time *t*:

2 $$c^{m}\left(t\right)=\frac{1}{N}\overset{N}{\underset{i=1}{\sum }}p^{i,m}\left(t\right)$$

The average angle deviation between the principal axes of the three strands, δ_θ_, is calculated as

3 $$\delta_{\theta }\left(t\right)=\frac{\left(\delta_{\theta }^{12}\left(t\right)+\delta_{\theta }^{23}\left(t\right)+\delta_{\theta }^{31}\left(t\right)\right)}{3}$$

where

4 $$\delta_{\theta }^{mn}\left(t\right)=\frac{\left|\theta^{mn}\left(t\right)-\theta^{mn}\left(0\right)\right|}{\theta^{mn}\left(0\right)}$$

with *m* and *n* are strand indexes and

5 $$\theta^{mn}\left(t\right)=\arccos\left(\frac{v_{1}^{m}\left(t\right)}{∥v_{1}^{m}\left(t\right)∥}\cdot \frac{v_{1}^{n}\left(t\right)}{∥v_{1}^{n}\left(t\right)∥}\right)$$

In this paper, we also define the average principal axis of the strands, $v_{1}^{\mathrm{avg}}\left(t\right)$ , as

6 $$v_{1}^{\mathrm{avg}}\left(t\right)=\frac{1}{3}\left(v_{1}^{1}\left(t\right)+v_{1}^{2}\left(t\right)+v_{1}^{3}\left(t\right)\right)$$

We use its normalized unit vector, ${\mathrm{v̂}}_{1}^{\mathrm{avg}}\left(t\right)$ , to define the shift of strand *m* with respect to strand *n* at time *t*, *s* ^*mn*^(*t*), as

7 $$s^{mn}\left(t\right)=\left|{\mathrm{v̂}}_{1}^{\mathrm{avg}}\left(t\right)\cdot \left(c^{m}\left(t\right)-c^{n}\left(t\right)\right)\right|$$

where (*m*, *n*) ∈ {(1, 2), (2, 3), (3, 1)}. The overall shift of the principal axes at time *t*, *s*(*t*), is defined as the largest of the three pairwise shifts:

8 $$s\left(t\right)=\max\left\{s^{12}\left(t\right),s^{23}\left(t\right),s^{31}\left(t\right)\right\}$$

#### Intrastrand Deformation

The rise, labeled Rise^*i*,*m*^(*t*) for residue *i* on strand *m*, is calculated as

9 $${\mathrm{Rise}}^{i,m}\left(t\right)=\left(p^{i+1,m}\left(t\right)-p^{i,m}\left(t\right)\right)\cdot \frac{v_{1}^{m}\left(t\right)}{∥v_{1}^{m}\left(t\right)∥}$$

where *i* ranges from 1 to *N* –1 (i.e., no rise is calculated for the end residue). We then calculate the deviation in Rise^*i*,*m*^(*t*) as

10 $$\delta_{\mathrm{Rise}}^{i,m}\left(t\right)=\frac{{\mathrm{Rise}}^{i,m}\left(t\right)-{\mathrm{Rise}}^{i,m}\left(0\right)}{{\mathrm{Rise}}^{i,m}\left(0\right)}$$

We also calculate the circumradius, *R* ^*i*,*m*^(*t*), of the triangle made by three consecutive C_α_ on strand *m* as

11 $$R^{i,m}\left(t\right)=\left\{∥p^{i,m}\left(t\right)-p^{i-1,m}\left(t\right)∥∥p^{i+1,m}\left(t\right)-p^{i-1,m}\left(t\right)∥∥p^{i,m}\left(t\right)-p^{i+1,m}\left(t\right)∥\right\}/\left\{4\times \frac{1}{2}∥\left(p^{i,m}\left(t\right)-p^{i-1,m}\left(t\right)\right)\times \left(p^{i+1,m}\left(t\right)-p^{i-1,m}\left(t\right)\right)∥\right\}$$

Note that here, *i* ranges from 2 to *N* –1 (i.e., the circumradius is not calculated for end residues).

We define deviations in *R* ^*i*,*m*^(*t*) as

12 $$\delta_{\mathrm{Radius}}^{i,m}\left(t\right)=\frac{R^{i,m}\left(t\right)-R^{i,m}\left(0\right)}{R^{i,m}\left(0\right)}$$

Finally, we define a single unitless metric, κ^*i*,*m*^(*t*), whose average captures the overall intrastrand deformation as

13 $$\langle \kappa^{i,m}\left(t\right)\rangle_{j}=0.5\langle \delta_{\mathrm{Rise}}^{i,m}\left(t\right)\rangle_{j}+0.5\langle \delta_{\mathrm{Radius}}^{i,m}\left(t\right)\rangle_{j}$$

where *j* can be *i*, *m,* and/or *t* for an average over the number of residues, strands, and/or time, respectively.

#### Interstrand Deformation

In this paper, we build upon the idea of a ″triad″ by Ravikumar and Hwang^27^ and form a triangle using three C_α_ at position *i* of each strand. We then calculate the area, Area^*i*^(*t*), of the triangle as

14 $${\mathrm{Area}}^{i}\left(t\right)=\frac{1}{2}∥\left(p^{i,2}\left(t\right)-p^{i,1}\left(t\right)\right)\times \left(p^{i,3}\left(t\right)-p^{i,1}\left(t\right)\right)∥$$

with deviation

15 $$\delta_{{\mathrm{Area}}^{i}}\left(t\right)=\frac{{\mathrm{Area}}^{i}\left(t\right)-{\mathrm{Area}}^{i}\left(0\right)}{{\mathrm{Area}}^{i}\left(0\right)}$$

The shape of this triangle, Shape^*i*^(*t*), is calculated as the normalized inverse of the isoperimetric ratio:

16 $${\mathrm{Shape}}^{i}\left(t\right)=\left[4\pi {\mathrm{Area}}^{i}\left(t\right)\right]/{\left(∥p^{i,2}\left(t\right)-p^{i,1}\left(t\right)∥+∥p^{i,3}\left(t\right)-p^{i,2}\left(t\right)∥+∥p^{i,1}\left(t\right)-p^{i,3}\left(t\right)∥\right)}^{2}$$

Shape^*i*^(*t*) ranges from 0 to 1, where 1 represents a perfect circle and 0.605 represents an equilateral triangle. The deviation in Shape^*i*^(*t*) is defined as

17 $$\delta_{{\mathrm{Shape}}^{i}(t)}=\frac{{\mathrm{Shape}}^{i}\left(t\right)-{\mathrm{Shape}}^{i}\left(0\right)}{{\mathrm{Shape}}^{i}\left(0\right)}$$

Similarly to what we have done for the rise and radius, we define a unitless metric ζ^*i*^(*t*), whose average quantifies the overall interstrand deformation as

18 $$\langle \zeta^{i}\left(t\right)\rangle_{j}=0.5\langle \delta_{{\mathrm{Area}}^{i}(t)}\rangle_{j}+0.5\langle \delta_{{\mathrm{Shape}}^{i}(t)}\rangle_{j}$$

where *j* can be *i* and/or *t* for an average over the number of residues and/or time.

### Characterizing *n* → π* Interactions

We calculate the Bürgi–Dunitz distance $d_{BD}^{i,m}=O^{i-1,m}-C^{i,m}$ and angle $\theta_{BD}^{i,m}=O^{i-1,m}-C^{i,m}=O^{i,m}$ for each residue *i* on strand *m* (Figure 2b). Note that here, *i* ranges from 2 to *N*. Previous work has shown that *n* → π* interactions are present if *d*_*BD*_ = 3.2 Å and θ_*BD*_ = 109°.^65,67,69^ We use an in-house code in Python3 to calculate normalized deviations from these references as

19a $$\delta \theta_{BD}^{i,m}\left(t\right)=\frac{\theta_{BD}^{i,m}\left(t\right)-E\left(\theta_{BD}\right)}{E\left(\theta_{BD}\right)}$$

19b $$\delta d_{BD}^{i,m}\left(t\right)=\frac{d_{BD}^{i,m}\left(t\right)-E\left(d_{BD}\right)}{E\left(d_{BD}\right)}$$

where *E*(θ_*BD*_) = 109° and *E*(*d*_*BD*_) = 3.2 Å. See Data Availability section for a link to the code.

### Calculating the Residence Times of Water and d-Glucose

We calculate the residence times of nearby water and d-glucose using the approach of Impey et al.,^97–99^ implemented in Python3 with in-house code. A water molecule is considered nearby if its oxygen atom is less than 5 Å away from the (PPG)_*n*_ CMP.

We then calculate a binary occupancy function, *h* ^*j*^(*t*) for each molecule *j* of type *J,* where *j* = *w* for water or *g* for d-glucose. *h* ^*j*^(*t*) = 1 if molecule *j* is nearby and 0 otherwise. Each continuous segment of *h* ^*j*^(*t*) = 1 is treated as an individual residence event *e* of duration *L* ^*j*^(*e*), defined as

20 $$L^{j}\left(e\right)=\left(t_{\mathrm{end}}^{j}\left(e\right)-t_{\mathrm{start}}^{j}\left(e\right)+1\right)\Delta t$$

where $t_{\mathrm{start}}^{j}$ and $t_{\mathrm{end}}^{j}$ denote the first and last frames of event *e* for molecule *j* and Δ*t* = 1 ps. The mean residence time over all residence events, $\tau_{J}^{\mathrm{res}}$ , is defined as

21 $$\tau_{J}^{\mathrm{res}}=\frac{1}{k_{J}^{\mathrm{ex}}}=\frac{\overset{N_{J}}{\underset{j=1}{\sum }}\overset{N_{e}^{j}}{\underset{e=1}{\sum }}L^{j}\left(e\right)}{\overset{N_{J}}{\underset{j=1}{\sum }}N_{e}^{j}}$$

where $k_{J}^{\mathrm{ex}}$ is the exchange rate, $N_{e}^{j}$ is the number of residence events observed for molecule *j*, and *N*_*J*_ is the total number of molecules of type *J* in the system.

### Characterizing the Translational Motion of Water Molecules in the Hydration Shell

We start with isolating the water molecules in the first hydration shell of the CMP at each time frame, using the RDF shown in Figure S5. The hydration shell is defined using 3.5 Å as the distance cutoff. We use the coordinates of the oxygen atoms **p** ^*O*,*w*^(*t*) in Cartesian axes (**x**, **y**, **z**) to calculate the translational velocity **v** ^*w*^(*t*) of water molecule *w* at times 0, *t*_max_, and *t* as

22a $$v^{w}\left(0\right)=\frac{p^{O,w}\left(\Delta t\right)-p^{O,w}\left(0\right)}{\Delta t}$$

22b $$v^{w}\left(t_{\max}\right)=\frac{p^{O,w}\left(t_{\max}\right)-p^{O,w}\left(t_{\max}-\Delta t\right)}{\Delta t}$$

22c $$v^{w}\left(t\right)=\frac{p^{O,w}\left(t+\Delta t\right)-p^{O,w}\left(t-\Delta t\right)}{2\Delta t}$$

where *t*_*max*_ = 100 ns and Δ*t* = 1 ps.

We then calculate the translational diffusion tensor $D^{\mathrm{trans}}\in R^{3\times 3}$ , as

23 $$D_{\alpha \beta }^{\mathrm{trans}}=\frac{1}{2}\int_{0}^{t_{\max}}\underset{D_{\alpha \beta }^{\mathrm{trans}}\left(t\right)}{\underset{︸}{\langle v_{\alpha }^{w}\left(0\right)v_{\beta }^{w}\left(t\right)\rangle_{w}}}dt$$

where α, β ∈ {*x*, *y*, *z*}, *t*_max_ = 100 ns, and the average is taken over water molecules. The diagonal elements of ***D*** ^trans^, $D_{xx}^{\mathrm{trans}},D_{yy}^{\mathrm{trans}},D_{zz}^{\mathrm{trans}}$ are used to quantify the translational anisotropy as

24 $$c_{\mathrm{aniso}}^{\mathrm{trans}}=\frac{\max\left\{D_{xx}^{\mathrm{trans}},D_{yy}^{\mathrm{trans}},D_{zz}^{\mathrm{trans}}\right\}-\min\left\{D_{xx}^{\mathrm{trans}},D_{yy}^{\mathrm{trans}},D_{zz}^{\mathrm{trans}}\right\}}{\frac{1}{3}\mathrm{Tr}\left(D^{\mathrm{trans}}\right)}$$

We also fit the trace of the time-dependent tensor ***D*** ^trans^(*t*) to a biexponential, which is the functional form commonly used in the literature^100,101^ (using scipy.curve_fit):

25 $$\mathrm{Tr}\left(D^{\mathrm{trans}}\left(t\right)\right)=A^{\mathrm{trans}}e^{-t/\tau_{1}^{\mathrm{trans}}}+B^{\mathrm{trans}}e^{-t/\tau_{2}^{\mathrm{trans}}}$$

where $\tau_{1}^{\mathrm{trans}},\tau_{2}^{\mathrm{trans}}$ are time constants and *A* ^trans^, *B* ^trans^ are their coefficients.

### Characterizing the Rotational Motion of Water Molecules in the Hydration Shell

We define the local frame of water *w* from the Cartesian coordinates of the water atoms at time *t*:

26a $$r_{3}^{w}\left(t\right)=p^{O}\left(t\right)-\frac{p^{H1}\left(t\right)+p^{H2}\left(t\right)}{2}$$

26b $$r_{1}^{w}\left(t\right)=\left(p^{H1}\left(t\right)-p^{O}\left(t\right)\right)-\left(\left(p^{H1}\left(t\right)-p^{O}\left(t\right)\right)\cdot r_{3}^{w}\left(t\right)\right)$$

26c $$r_{2}^{w}\left(t\right)=r_{1}^{w}\left(t\right)\times r_{3}^{w}\left(t\right)$$

where **p** ^*O*,*w*^(*t*), **p** ^*H*1,*w*^(*t*), and **p** ^*H*2,*w*^(*t*) are the Cartesian coordinates of the oxygen and hydrogen atoms of water *w* at time *t*, respectively.

We use the components of these vectors $r_{1}^{w}\left(t\right)={[r_{1x}^{w}(t),r_{1y}^{w}(t),r_{1z}^{w}(t)]}^{T}$ , $r_{2}^{w}\left(t\right)={[r_{2x}^{w}(t),r_{2y}^{w}(t),r_{2z}^{w}(t)]}^{T}$ , and $r_{3}^{w}\left(t\right)={[r_{3x}^{w}(t),r_{3y}^{w}(t),r_{3z}^{w}(t)]}^{T}$ to define the following rotation matrix **R** ^*w*^(*t*):

27 $$R^{w}\left(t\right)=\left[\begin{array}{lll} \frac{r_{1x}^{w}\left(t\right)}{∥r_{1}^{w}\left(t\right)∥} & \frac{r_{1y}^{w}\left(t\right)}{∥r_{1}^{w}\left(t\right)∥} & \frac{r_{1z}^{w}\left(t\right)}{∥r_{1}^{w}\left(t\right)∥}\\ \frac{r_{2x}^{w}\left(t\right)}{∥r_{2}^{w}\left(t\right)∥} & \frac{r_{2y}^{w}(t}{∥r_{2}^{w}\left(t\right)∥}) & \frac{r_{2z}^{w}\left(t\right)}{∥r_{2}^{w}\left(t\right)∥}\\ \frac{r_{3x}^{w}\left(t\right)}{∥r_{3}^{w}\left(t\right)∥} & \frac{r_{3y}^{w}\left(t\right)}{∥r_{3}^{w}\left(t\right)∥} & \frac{r_{3z}^{w}\left(t\right)}{∥r_{3}^{w}\left(t\right)∥} \end{array}\right]$$

**R** ^*w*^(*t*) transforms any local coordinate vector into a global coordinate vector through **u**_global_(*t*) = **R** ^*w*^(*t*) **u**_local_(*t*). We then define the unit quaternion **Q** ^*w*^(*t*) that represents **R** ^*w*^(*t*) as

28 $$Q^{w}\left(t\right)={[q_{0}^{w}(t),q^{w}\left(t\right)]}^{T}$$

where the real part $q_{0}^{w}\left(t\right)$ is related to the angle of rotation and the imaginary part $q^{w}\left(t\right)={\left(q_{1}^{w}(t),q_{2}^{w}(t),q_{3}^{w}(t)\right)}^{T}$ is the rotation axis. The components of **Q** ^*w*^(*t*) are calculated from the components of **R** ^*w*^(*t*) using the Shepperd method,^102,103^ as detailed in Supporting Information.

Angular velocities for each water *w* at time *t*, **ω** ^*w*^(*t*), can then be computed in the local frame as^104^

ωw(t)=2Im((Qw(t))*∂Qw(t)∂t) 29

where ${\left(Q^{w}\left(t\right)\right)}^{*}={[q_{0}^{w}(t),-q^{w}\left(t\right)]}^{T}$ is the complex conjugate of **Q** ^*w*^(*t*). The calculation of the components of **ω** ^*w*^ (*t*) from the components of **Q^*w*^(*t*)** is shown in Supporting Information.

We use **ω** ^*w*^(*t*) to define the rotational diffusion tensor ***D*** ^rot^ as

30 $$D_{\alpha \beta }^{\mathrm{rot}}=\frac{1}{2}\int_{0}^{t_{\max}}\underset{D_{\alpha \beta }^{\mathrm{rot}}\left(t\right)}{\underset{︸}{\langle \omega_{\alpha }^{w}\left(0\right)\omega_{\beta }^{w}\left(t\right)\rangle_{w}}}dt$$

where $\alpha ,\beta \in \left\{r_{1}^{w},r_{2}^{w},r_{3}^{w}\right\}$ , *t*_max_ = 100 ns, and the average is taken over water molecules. Rotational anisotropy was calculated as^104^

31 $$c_{\mathrm{aniso}}^{\mathrm{rot}}=\frac{\max\left\{D_{r_{1}r_{1}}^{\mathrm{rot}},D_{r_{2}r_{2}}^{\mathrm{rot}},D_{r_{3}r_{3}}^{\mathrm{rot}}\right\}-\min\left\{D_{r_{1}r_{1}}^{\mathrm{rot}},D_{r_{2}r_{2}}^{\mathrm{rot}},D_{r_{3}r_{3}}^{\mathrm{rot}}\right\}}{\frac{1}{3}\mathrm{Tr}\left(D^{\mathrm{rot}}\right)}$$

We also process the trace of the time-dependent tensor ***D*** ^rot^(*t*) with a second-order Legendre polynomial to facilitate fitting to a monoexponential, using scipy.curve_fit, as

32 $$\frac{3\mathrm{Tr}(D^{\mathrm{rot}}\left(t\right){)}^{2}-1}{2}=A^{\mathrm{rot}}e^{-t/\tau^{\mathrm{rot}}}$$

where τ^rot^ is the time constant and *A* ^rot^ is the coefficient.

A link to the codes used to quantify hydration dynamics is provided in the Data Availability section.

## Results and Discussion

### Conformational Flexibility of (PPG)*_n_* CMP Strands

In Figure 3, we show the Ramachandran plots of Pro at position Xaa of (PPG)_5_, (PPG)_12_, and (PPG)_25_ with A4P (left) and AMOEBA (right). The Ramachandran plots for C3P, C4P, and Cm4P are shown in Figure S10. We observe that all force fields yield the same averages, ⟨ψ­(*t*), ϕ­(*t*)⟩_*t*_, within 10°. For example, ⟨ψ­(*t*),ϕ­(*t*)⟩*_t_* = (147° ± 10°, −55° ± 13°), (152° ± 9°, −64° ± 12°), (159° ± 19°, −65° ± 12°^°^), (156° ± 17°, −71° ± 16°), and (158° ± 18°, −62° ± 25°) for (PPG)_12_ with C3P, C4P, Cm4P, A4P, and AMOEBA, respectively (Table S2). These results compare well with the (164°,–77°) reported from experimental characterization of (PPG)_*n*_ CMPs.^24,25,48,105,106^ However, we note in Figure 3 that ψ­(*t*) and ϕ­(*t*) stay within 30° of that average during the whole duration of the C3P, C4P, Cm4P, and A4P MD simulations, while there are a lot more variations in the AMOEBA MD simulations. Overall, the standard deviation in (ψ­(*t*), ϕ­(*t*)) with AMOEBA is over two times that with the other force fields (Table S2). This higher standard deviation in (ψ­(*t*), ϕ­(*t*)) indicates that the backbone of the CMP strands is a lot more flexible with AMOEBA, consistent with residue-water and water–water interactions of similar magnitudes. Similar observations can be made from the Ramachandran plots of Pro at position Yaa and Gly (Figure S10). Further analysis indicates that the large standard deviation is characteristic of deformation of the triple helix rather than excessive flexibility caused by overly favorable water interactions in AMOEBA (Figure S11).

**Figure 3:**
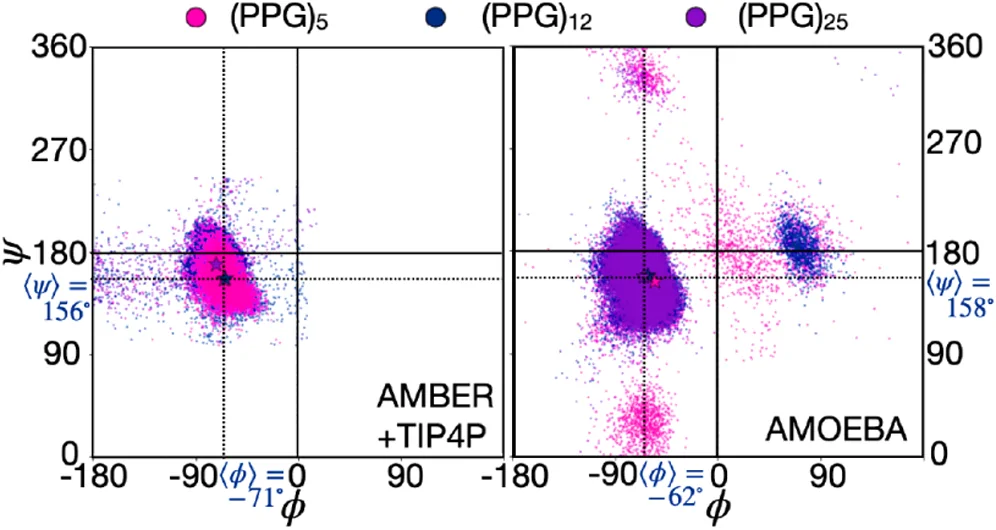
Ramachandran plots of Pro at position Xaa for (PPG)_5_, (PPG)_12_, and (PPG)_25_ modeled with A4*P* = AMBER + TIP4P (left) and AMOEBA (right). $\psi \left(t\right)=N^{i}-C_{\alpha }^{i}-C^{i}-N^{i+1}$ and $\phi \left(t\right)=C^{i-1}-N^{i}-C_{\alpha }^{i}-C^{i}$ . In each plot, the stars indicate the time average ⟨ψ­(*t*), ϕ­(*t*)⟩*_t_*. The averages for (PPG)_12_ are emphasized with dotted lines.

While ψ­(*t*) and ϕ­(*t*) characterize (PPG)_*n*_ backbone flexibility, ω­(*t*) specifies the configuration of the peptide bond. We provide the averages and standard deviations of ω­(*t*) in Table S2. In our simulations, all force fields yield ⟨ω­(*t*)⟩_*t*_ = 176 ± 5° for all (PPG)_*n*_ CMPs. This value of ⟨ω­(*t*)⟩_*t*_ means that the *trans* isomer is predominant, which agrees with experiments where ω_exp_ = 178°.^24,25,48,105,106^

In Table S3, we show the average and standard deviation of the pyrrolidine ring pucker angle, ⟨χ­(*t*)⟩_*t*_, of Pro at positions Xaa and Yaa. Negative ⟨χ­(*t*)⟩_*t*_ specifies the exo pucker, often associated with a more compact conformation of the protein backbone.^25,48,105^ Positive ⟨χ­(*t*)⟩_*t*_ specifies the endo pucker, favoring more extended conformations.^25,48,105^ For the exo pucker, we find ⟨χ_exo_(*t*)⟩_*t*_ to be −27° ± 8°, −27° ± 4°, −27° ± 8°, −32° ± 9°, and −33° ± 12° for C3P, C4P, Cm4P, A4P, and AMOEBA, respectively. This is consistent with the −30° reported experimentally.^1,24,25,48,105,106^ Similarly, we observe good agreement between force fields and between theory and experiments for the endo pucker, where the average ⟨χ_endo_(*t*)⟩_*t*_ is 29° ± 8°, 29° ± 5°, 29° ± 8°, 34° ± 9°, 34° ± 11°, and 31° for C3P, C4P, Cm4P, A4P, AMOEBA, and experiments, respectively. However, there is a significant difference in the relative population of exo:endo puckers during the MD simulations. With C3P and C4P, we report a dominant exo pucker for Pro at positions Xaa (75:25) and Yaa (85:15) for (PPG)_5_, (PPG)_12_, and (PPG)_25_. With Cm4P, A4P, and AMOEBA, we report a dominant exo pucker for Pro at the position Yaa (75:25) for (PPG)_5_, (PPG)_12_, and (PPG)_25_, but a more evenly weighted 50:50 ratio at Xaa. These pucker ratios are in agreement with the 50:50 and 78:22 pucker ratios reported experimentally for Pro at positions Xaa and Yaa, respectively (Table S3).^24,25,48,105,106^ This means that Cm4P, A4P, and AMOEBA yield better results than C3P and C4P in the predicting χ­(*t*), yielding more extended CMP strands. These results align with our expectations, as CHARMM36mGP and AMBER have been reported to reproduce puckering propensities more accurately than CHARMM36.^54^

Finally, we calculate deviations in the Bürgi–Dunitz distance, $\delta d_{BD}^{i,m}\left(t\right)$ (eq 19) and angle $\delta \theta_{BD}^{i,m}\left(t\right)$ , (eq 19) for residue *i* on strand *m*. These deviations characterize the geometric features of CMP strands associated with the subtle noncovalent *n* → π* interactions, which have been predicted through density functional theory.^66,67,69,105,107^ $\delta d_{BD}^{i,m}\left(t\right)$ for (PPG)_12_ as a function of time and residue index is shown in Figure S12a. We find that Cm4P maintains $\delta d_{BD}^{i,m}\left(t\right)$ less than 0.1 for 89% of the time, compared to 85% with A4P and 82% with AMOEBA. This suggests that the CMPs are more constrained with Cm4P than with A4P, and most flexible with AMOEBA. However, we see that Cm4P maintains $\delta \theta_{BD}^{i,m}\left(t\right)$ less than 0.1 only 10% of the time, compared to 16% for A4P and 19% for AMOEBA (Figure S12b). Interestingly, we note that only 9.7% of the frames simultaneously verify both conditions in Cm4P, compared to 13% in A4P and 16% in AMOEBA (Figure S12c). This indicates that AMOEBA provides a relatively better description of *n* → π* interactions, accommodating flexible (PPG)_*n*_ CMP strands. Similar observations can be made for (PPG)_25_, where 13.5% of the frames simultaneously verify both conditions with AMOEBA, compared to 8.9% with Cm4P and 10.8% with A4P (Figure S13). Older density functional theory calculations on single (PPG) units predict that *n* → π* interactions lower the energy by 0.1 to 0.46 kcal/mol.^66,69^ A 0.1 (0.46) kcal/mol energy difference yields a 54% (68%) Boltzmann weight for the conformations with *n* → π* interactions. A newer study using M062*X*/6–31+G­(d,p)^108^ identifies the energy to be 0.9 kcal/mol, which translates to an 81% Boltzmann weight. However, these calculations were performed in continuum solvent and therefore represent upper limits for the energy stabilization provided by *n* → π* interactions. In reality, these interactions compete with hydrogen bonding to explicit water molecules, which would also lower the overall energy of the peptides. Further, the *n* → π* interactions predicted on single (PPG) units would likely be disrupted on a longer CMP modeled at 310 K, like we do here. In our simulations, AMOEBA produces CMP length-dependent results, closer to our expectations, although neither approaches the upper limit set by density functional theory.

### Deformation of the Triple Helix during the MD Simulations

Here, we calculate the overall deformation of the strands in each (PPG)_*n*_ CMP. When the triple helix is intact, as in the beginning of the simulations, the principal axes of the three strands are aligned and θ­{^*mn*^(*t*) (eq 4) is zero for every strand pair. Then, the deviation δ_θ_(*t*) (eq 3) is negligible. In Figure 4a, we show ⟨δ_θ_(*t*)⟩_*t*_ for (PPG)_5_, (PPG)_12_, and (PPG)_25_ with C3P, C4P, Cm4P, A4P, and AMOEBA. We see that, with AMOEBA, ⟨δ_θ_(*t*)⟩_*t*_ = 0.7 ± 0.2 for (PPG)_5_, which is greater than the 0.2 ± 0.1 for (PPG)_12_ and the 0.0 ± 0.0 for (PPG)_25_. This means that the principal axes of the three strands in (PPG)_25_ remain aligned during the simulation, while those in (PPG)_5_ do not. This length-dependent deformation is expected.^10,50–52,78,113^ Indeed, we ran our MD simulations at 310 K, which is higher than the melting temperature of (PPG)_5_ (<283 K)^50,51^ and is very close to the melting temperature of (PPG)_12_ (313 K).^50,51^ In contrast, the melting point of (PPG)_25_ is greater than 335 K,^50,51^ such that we do not expect deformation at 310 K. We see a similar trend, although with a much lower magnitude, with A4P, where ⟨δ_θ_(*t*)⟩_*t*_ = 0.2 ± 0.1, 0.1 ± 0.0, 0.0 ± 0.0 for (PPG)_5_, (PPG)_12_, and (PPG)_25_, respectively. With C3P, C4P, and Cm4P, ⟨δ_θ_(*t*)⟩_*t*_ is close to zero for all lengths of CMP, exposing a nonphysical behavior. Similar observations can be made for the average shift of the principal axes, ⟨*s*(*t*)⟩_*t*_ (eq 8, Figure 4b and S8b). With AMOEBA, ⟨*s*(*t*)⟩_*t*_ = 13 ± 7, 9 ± 6, and 7 ± 4 Å for (PPG)_5_, (PPG)_12_, and (PPG)_25_, respectively. However, with C3P, C4P, Cm4P, and A4P, ⟨*s*(*t*)⟩_*t*_ is not inversely proportional to *n* and all three (PPG)_*n*_ CMPs exhibit a 6 Å shift. This shift is of the same magnitude as the shift reported in crystal structures of (PPG)_10<*n*<15_ CMPs. Since our starting structures mimic the crystal structure, this indicates that C3P, C4P, Cm4P, and A4P preserve the initial geometry and do not adapt to solvent, temperature, or CMP length. Meanwhile, AMOEBA allows for more realistic deformations over time. These relative differences are visible in Figure 5a-b, where we show snapshots at the end of the C3P, C4P, Cm4P, A4P, and AMOEBA MDs for (PPG)_12_. The snapshots for PPG_5_ and PPG_25_ are provided in Figure S15.

**Figure 4:**
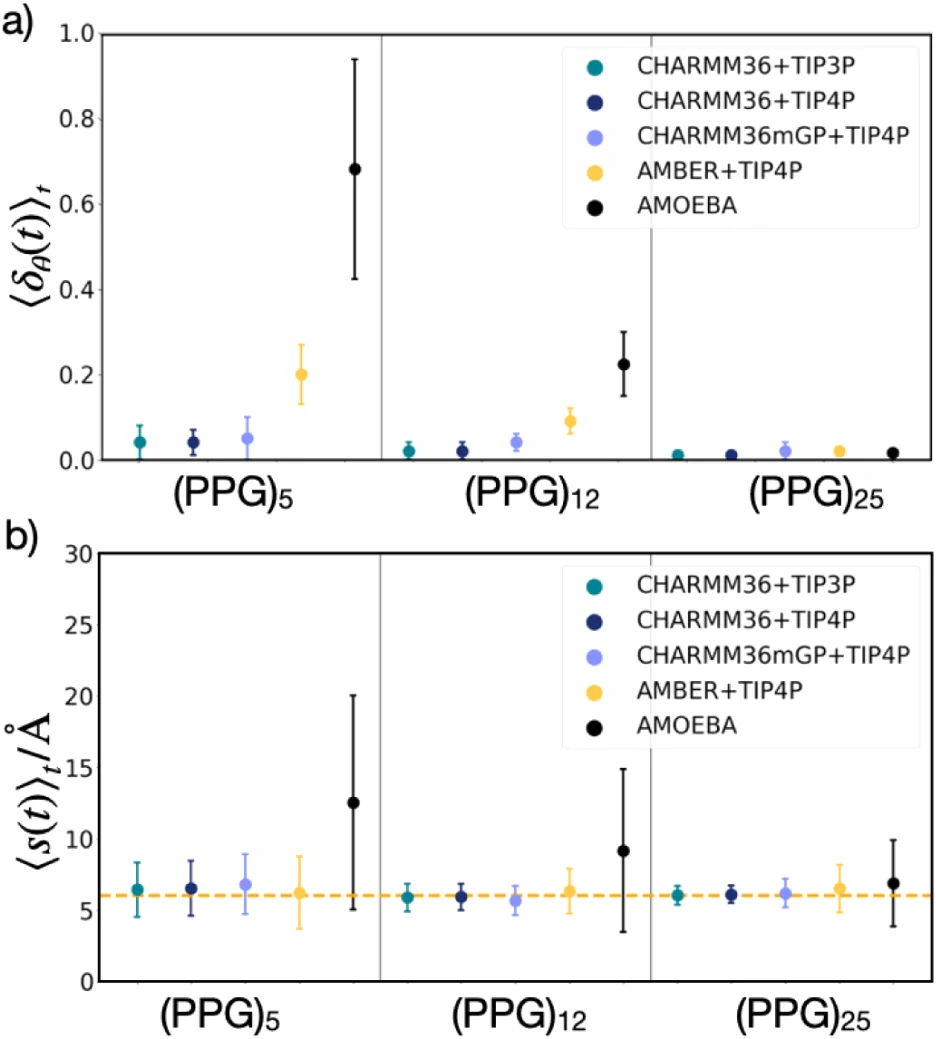
a) ⟨δ_θ_(*t*)⟩*_t_* for (PPG)_5_, (PPG)_12_, and (PPG)_25_ from C3*P* = CHARMM36 + TIP3P, C4*P* = CHARMM36 + TIP4P, Cm4*P* = CHARMM36mGP+TIP4P, A4*P* = AMBER + TIP4, and AMOEBA. b) Average axial shift of one strand with respect to another for C3*P* = CHARMM36 + TIP3P, C4*P* = CHARMM36 + TIP4P, Cm4*P* = CHARMM36mGP + TIP4P, A4*P* = AMBER +TIP4P, and AMOEBA. The orange line shows the approximate axial shift from crystal structures of CMPs with 10 ≤ *n* ≤ 15.^109–112^

**Figure 5:**
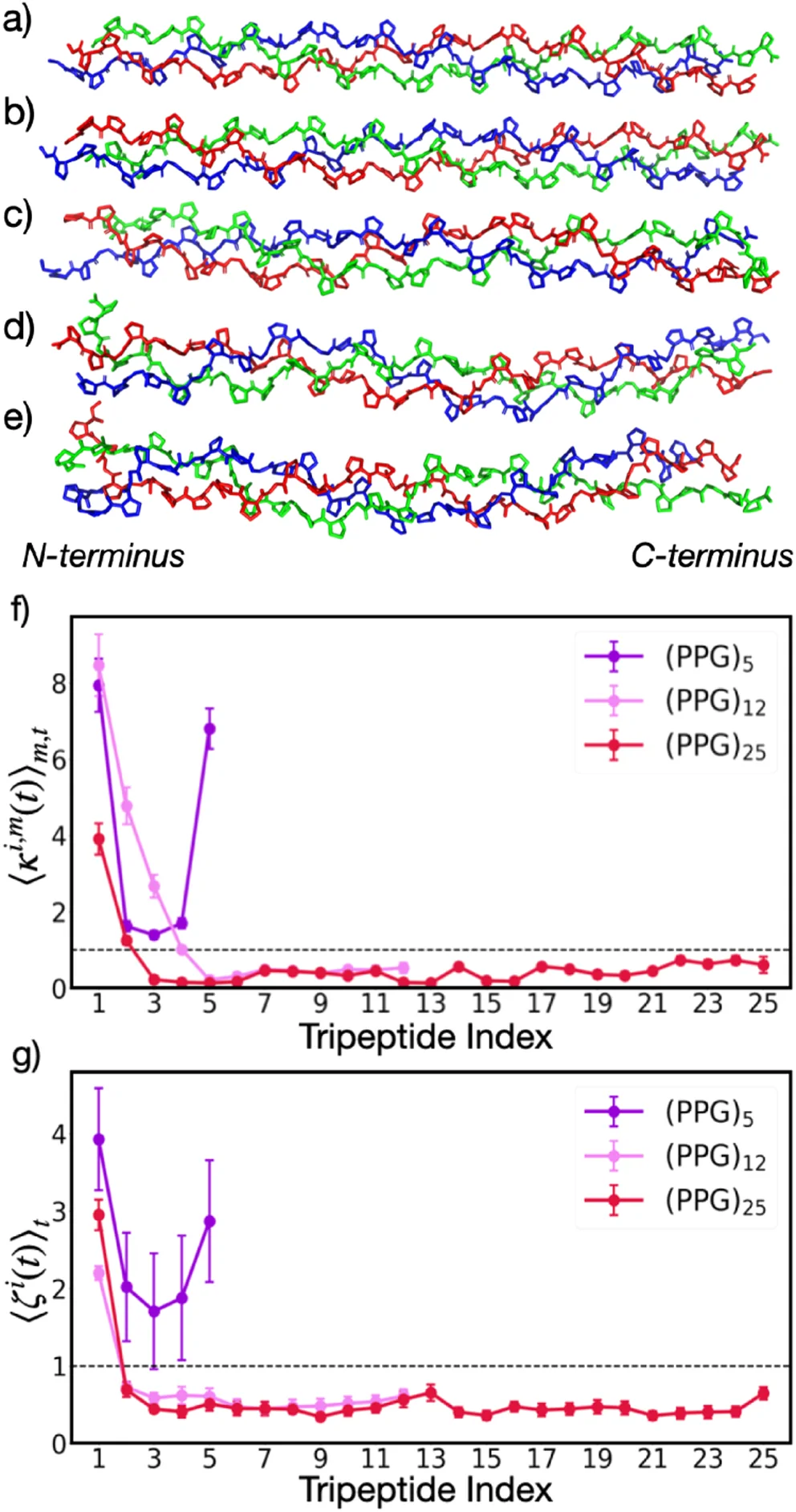
Snapshots for (PPG)_12_ at the end of a) C3*P* = CHARMM36 + TIP3P, b) C4*P* = CHARMM36 + TIP4P, c) Cm4*P* = CHARMM36mGP + TIP4P, d) A4*P* = AMBER + TIP4P, and e) AMOEBA MD simulations, with the N- (left) and C-termini (right) labeled. Water molecules and hydrogen atoms are omitted for clarity. Each strand is colored differently in blue, red, and green. f) ⟨κ^*i,m*^(*t*)⟩_*m,t*_ for (PPG)_12_ over tripeptide index. g) ⟨ζ*^i^* (*t*)⟩*_t_* for (PPG)_12_ over tripeptide index.

To further resolve these deformations at the tripeptide scale, we introduce two unitless metrics, κ^*i*,*m*^(*t*) (eq 13), which quantifies intrastrand deformation, and ζ^*i*^(*t*) (eq 18), which quantifies interstrand deformation. However, since our observations thus far have repeatedly shown that AMOEBA yields to greater (PPG)_*n*_ CMP deformation, we will focus on analyzing AMOEBA trajectories exclusively.

Figure 5d and e showS ⟨κ^*i*,*m*^(*t*)⟩_*m*,*t*_ and ⟨ζ^*i*^(*t*)⟩_*t*_ for (PPG)_5_, (PPG)_12_, and (PPG)_25_ with AMOEBA. For both metrics, we considered a tripeptide deformed if the value exceeded 1. That threshold corresponds to the amount of deformation where κ^*i*,*m*^(*t*) and ζ^*i*^(*t*) are twice κ^*i*,*m*^(0) and ζ^*i*^(0), respectively. Although the choice of a threshold of 1 is not unique, the observed trends and overall conclusions are robust across a broad range of cutoff values.

For the intrastrand deformation, we see that ⟨{κ^*i*,*m*^(*t*)⟩_*m*,*t*_ ≥ 1 for all tripeptides in (PPG)_5_. In contrast, only tripeptides #1 and #2 at the N-terminus of (PPG)_25_ are deformed, with ⟨κ^*i*,*m*^(*t*)⟩_*m*,*t*_ = 3.9 ± 0.4 and 1.3 ± 0.1, respectively. (PPG)_12_ deforms more than (PPG)_25_ but less than (PPG)_5_, with the first four tripeptides at the N-terminus being deformed. We see a similar trend for the interstrand deformation with ⟨ζ^*i*^(*t*)⟩_*t*_. Indeed, all tripeptides in (PPG)_5_ are deformed, but only the first N-terminus tripeptide in (PPG)_12_ and (PPG)_25_ are deformed, with ⟨ζ^*i*^(*t*)⟩_*t*_ = 2.2 ± 0.1 and 3.0 ± 0.2, respectively. The fact that the N-terminus is more deformed than the C-terminus is consistent with the direction of triple helix unfolding previously reported.^111,114–116^ The time dependence of ⟨κ^*i*,*m*^(*t*)⟩_*m*_ and ζ^*i*^(*t*) is shown in Figure S16. Movie S1 illustrates the correlation between ⟨κ^*i*,*m*^(*t*)⟩_*i*,*m*_ and ⟨ζ^*i*^(*t*)⟩_*i*_ in (PPG)_12_ over the course of the AMOEBA simulation. We identify two populations, the first with ⟨ζ^*i*^(*t*)⟩_*i*_ ≤ 0.75 and variable ⟨κ^*i*,*m*^(*t*)⟩{_*i*,*m*_, and the second with ⟨ζ^*i*^(*t*)⟩_*i*_ ≥ 0.75 and variable ⟨κ^*i*,*m*^(*t*)⟩_*i*,*m*_. Initially, from 0 to approximately 17 ns, the triple helix undergoes only intrastrand deformation, with no significant interstrand deformation, corresponding to the first population. At around 19 ns, partial strand separation occurs and persists for 3 to 4 ns, corresponding to the second population, after which the triple helix returns to a regime dominated by intrastrand deformation—elongation and compression—of each strand for approximately 20 ns. This alternating sequence of localized strand separation followed by periods of primarily intrastrand deformation continues throughout the 100 ns trajectory. This suggests a progression in which local strand elongation or compression destabilizes the interstrand contacts, eventually permitting separation. Overall, the triple helix in (PPG)_12_ retains structural integrity despite significant deformation within each strand. This validates our use of ⟨κ^*i*,*m*^(*t*)⟩_*m*,*t*_ and ⟨ζ^*i*^(*t*)⟩_*t*_, which provide a novel quantification of triple helix deformation for systematic analysis of CMPs.

### Hydrogen Bonds and Hydration Analysis

Figure 6 shows the number of interstrand HBs per tripeptide for (PPG)_12_, which includes Gly­(NH)-Xaa­(CO) and aliphatic Xaa­(C_α_)-CO HBs. No other backbone atoms form a direct HB with another strand in our simulations. All MDs start with one interstrand Gly­(NH)-Xaa­(CO) HB per tripeptide.^1,21–24,36,117^ However, we observe several of these HBs breaking over time such that the average number becomes 0.2 ± 0.2, 0.75 ± 0.2, and 0.8 ± 0.2 HBs per tripeptide for (PPG)_5_, (PPG)_12_, and (PPG)_25_, respectively (Table S4). Unsurprisingly, the HBs break predominantly at the N-terminus of each CMP, where we found greater strand deformation. Overall, AMOEBA predicts Gly­(NH)-Xaa­(CO) HBs to have an average N–O distance of 2.9 ± 0.1 Å, an average H···O distance of 2.3 ± 0.2 Å, and an average angle of 27 ± 3°. These results are consistent with the literature, where N–O distances are reported to be 2.9 Å and H···O distances are 2.2 Å with an angle of 30°.^21,23,24,36^ For Xaa­(C_α_)-CO HBs, we start with a total of 1, 1, and 2 HBs for (PPG)_5_, (PPG)_12_, and (PPG)_25_, respectively. However, we find the average number of these HBs to be 0, 3, and 5 (Table S4), which indicates that Xaa­(C_α_)-CO HBs are formed during the simulation. These HBs have an average C_α_–O distance of 3.1 ± 0.2 Å, an H···O distance of 2.5 ± 0.1 Å, and an angle of 28 ± 2°. These values agree with the C_α_–O distance of 3.2 Å, H···O distance of 2.4 Å, and angles varying between 30° and 40° reported in the literature.^1,21–24,36^

**Figure 6:**
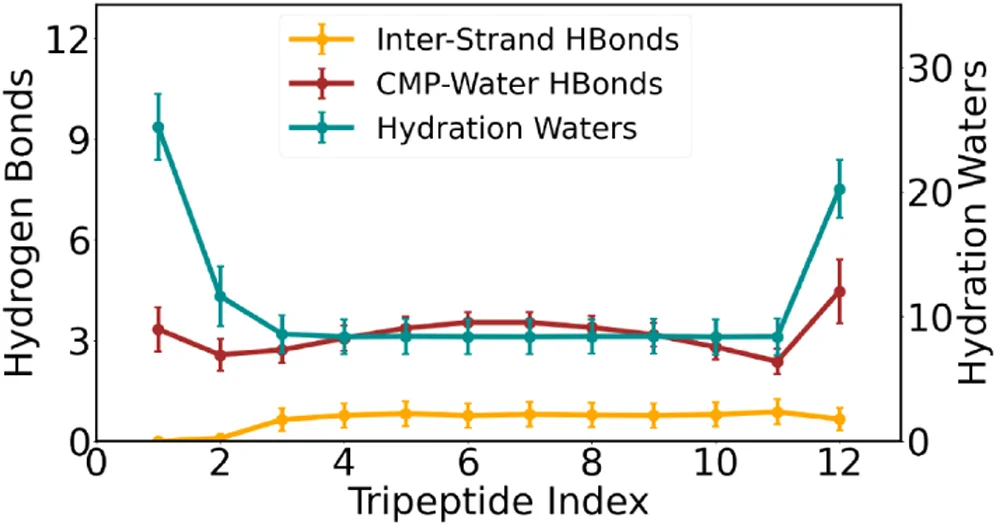
Average interstrand HBs, CMP-water HBs, and hydration waters for each tripeptide for (PPG)_12_ in AMOEBA.

Figure 6 also shows the number of HBs that each tripeptide in (PPG)_12_ forms with water. We see that both end tripeptides form 4 HBs with water, compared to 3 for the other tripeptides. For (PPG)_25_, the first three tripeptides form 7 HBs with water each, compared to 4 for the other tripeptides (Figure S17). Meanwhile, the number of HBs with water was constant at 4 in (PPG)_5_ (Figure S17). A subset of these (PPG)_*n*_-water HBs are the HBs between water and the CO group. In our simulations, the average numbers of CO-water HBs are 1.1 ± 0.3 HBs for the CO of Pro and 1.0 ± 0.2 for the CO of Gly. This compares well with experimental literature, where the average number of CO-water HBs was found to be 1.1.^36,105^

Finally, we show in Figure 6 the number of waters in the hydration shell of (PPG)_12_ CMP. We see that there are between 5 and 10 water molecules around each tripeptide engaged in the triple helix. This number rises to over 20 for the end tripeptides. Interestingly, the end tripeptide at the N-terminus is exposed to five more waters than the end tripeptide at the C-terminus. Similar observations can be made for (PPG)_5_ and (PPG)_25_ (Figure S17). Overall, the tripeptides we identified as deformed in each CMP are exposed to more water than nondeformed tripeptides. Table 1 shows the translational and rotational diffusion coefficients of these hydration-shell waters. We find $D_{\mathrm{iso}}^{\mathrm{trans}}=1.1$ to 1.2 × 10^–5^ cm^2^/s for all (PPG)_*n*_. For comparison, the diffusion coefficient of water in the bulk at 310 K is $D_{\exp}^{\mathrm{trans}}=3\times 10^{-5}{\mathrm{cm}}^{2}/s$ .^118–120^ This indicates that the waters in the hydration shell of (PPG)_*n*_ diffuse 2.5 to 2.7 times slower than in the bulk. This slow down is consistent with the 2 to 3 retardation factor reported in other proteins.^121–125^ These diffusion kinetics also correspond to an asymmetric distribution of individual water residence times, with a long tail at long lifetimes (≥20 ps) and a median of 3 ps (Figure S18). Over 50% of the residence events are less than 5 ps, which is typical of protein hydration waters.^98,126^ Finally, we characterize the translational relaxation dynamics by fitting the decay of Tr­(*D* ^trans^(*t*)) to a biexponential (eq 28, Figure S19a,c,e) for (PPG)_*n*_. We find $\tau_{1}^{\mathrm{trans}}=0.3\mathrm{ps}$ and $\tau_{2}^{\mathrm{trans}}=3.15\mathrm{ps}$ for (PPG)_12_ (Figure S19c). The short timescale corresponds to fast relaxations within the local cage of HBs, while the longer component reflects escape into the broader hydration environment. These time constants are within the range reported for various proteins, where $\tau_{1}^{\mathrm{trans}}\leq 1\mathrm{ps}$ and $2\leq \tau_{2}^{\mathrm{trans}}\leq 7.5\mathrm{ps}$ .^121,122,127^ These time constants are relatively consistent in the other (PPG)_*n*_, with $\tau_{1}^{\mathrm{trans}}=0.28$ and 0.35 ps, $\tau_{2}^{\mathrm{trans}}=3.15$ and 3.35 ps for (PPG)_5_ and (PPG)_25_, respectively (Figure S19). For rotational dynamics, we fit 3­((Tr­(*D* ^rot^(*t*))^2^ – 1)/2 to a single exponential (eq 37, Figure S19b,d,f). We find τ^rot^ = 0.65, 0.78, and 0.80 ps for (PPG)_5_, (PPG)_12_, and (PPG)_25_, respectively. These are close to the τ^rot^ ≈ 1 ps reported in the literature.^128–130^

**Table 1:** Comparison of the Diagonal Elements of ***D*** ^trans^ and ***D*** ^rot^ for the Entire (PPG)_5_, (PPG)_12_, and (PPG)_25_ Triple Helices, Along with $D_{\mathrm{iso}}^{\mathrm{trans}}$ , $D_{\mathrm{iso}}^{\mathrm{rot}}$ , $c_{\mathrm{aniso}}^{\mathrm{trans}}$ , and $c_{\mathrm{aniso}}^{\mathrm{rot}}$ ^a^

$D_{kk}^{\mathrm{trans}}$ (10–^5^ cm^2^/s)	(PPG)_5_	(PPG)_12_	(PPG)_25_
$D_{\mathrm{iso}}^{\mathrm{trans}}$	1.1	1.2	1.2
$D_{xx}^{\mathrm{trans}}$	1.2	1.5	1.4
$D_{yy}^{\mathrm{trans}}$	1.2	1.2	1.3
$D_{zz}^{\mathrm{trans}}$	1.0	1.0	1.0
$c_{\mathrm{aniso}}^{\mathrm{trans}}$	0.02	0.44	0.38

$D_{kk}^{\mathrm{rot}}$ (rad^2^/s)	(PPG)_5_	(PPG)_12_	(PPG)_25_
$D_{\mathrm{iso}}^{\mathrm{rot}}$	1.3	1.2	1.3
$D_{r_{1}r_{1}}^{\mathrm{rot}}$	1.3	1.4	1.4
$D_{r_{2}r_{2}}^{\mathrm{rot}}$	1.4	1.3	1.4
$D_{r_{3}r_{3}}^{\mathrm{rot}}$	1.1	1.0	1.1
$c_{\mathrm{aniso}}^{\mathrm{rot}}$	0.03	0.37	0.28

a 0.1 ≤ *err* ≤ 0.3 for all elements of ***D*** ^trans^ and ***D*** ^rot^. The error of the mean (*err*) is calculated using the standard deviation divided by $\sqrt{5}$ , where 5 corresponds to the 5 nonoverlapping trajectory Segments of 20 ns each .

These results further validate the benefits of using the polarizable AMOEBA force field, which has proven to reliably form and break protein–protein and protein–water HBs in response to thermal noise and to correctly predict water kinetics. In our simulations, we conclude that a greater number of HBs with water is associated with greater deformation, although they are localized at the fraying ends of the CMPs. Interestingly, we also find variations in the magnitudes of the components of $D_{\mathrm{iso}}^{\mathrm{trans}}$ . Specifically, we see in Table 1 that $D_{zz}^{\mathrm{trans}}$ is consistently lower than $D_{xx}^{\mathrm{trans}}$ and $D_{yy}^{\mathrm{trans}}$ Since the CMP principal axes are oriented along the **z**-axis in our simulations, these results indicate that water diffuses more slowly along the triple helix than perpendicular to it. We quantify this anisotropy in diffusion with the parameter $c_{\mathrm{aniso}}^{\mathrm{trans}}$ (eq 27). $c_{\mathrm{aniso}}^{\mathrm{trans}}$ is almost zero for (PPG)_5_, which reflects the fact that (PPG)_5_ is short and almost completely deformed during the simulation, eliminating any directional preference. Meanwhile, $c_{\mathrm{aniso}}^{\mathrm{trans}}$ = 0.44 and 0.38 for (PPG)_12_ and (PPG)_25_, respectively. Such anisotropy is absent in bulk water,^131,132^ highlighting its direct origin in the confined collagen environment. A similar anisotropic pattern was observed in the rotational diffusion coefficients. In this case, water is slower around the **r_3**-axis (the water dipole).

### Influence of Stress Factors on (PPG)*_n_* CMPs

In this section, we examine how d-glucose affects the structure of (PPG)_12_ CMPs. The inclusion of d-glucose is motivated by the role of reducing sugars in collagen chemistry, including nonenzymatic protein glycation.^133–136^ In this work, d-glucose concentrations of 500–2000 mM were used as controlled systems to probe how increasing sugar concentration influences the structural properties of collagen-mimetic triple helices. The elevated concentrations chosen increase d-glucose occupancy near the CMP, providing adequate sampling of glucose–CMP interactions within accessible MD timescales. They also create progressively solute-rich local environments around the peptide, allowing us to examine how increased molecular packing near the CMP influences its structural behavior. Although d-glucose alone does not reproduce the full chemical complexity of *in vivo* crowding, these systems capture a relevant aspect of crowded environments: an increased local abundance of surrounding molecules near the protein surface. We further introduce targeted sequence substitutions to examine how local residue identity and side-chain chemistry modulate the response of (PPG)_12_ triple helices. $\mathrm{Pro}\overset{\mathrm{mid}}{\to }\mathrm{Lys}$ and $\mathrm{Pro}\overset{\mathrm{end}}{\to }\mathrm{Lys}$ substitutions introduce amine-containing side chains relevant to glycation chemistry, whereas $\mathrm{Gly}\overset{\mathrm{mid}}{\to }\mathrm{Ala}$ and $\mathrm{Gly}\overset{\mathrm{end}}{\to }\mathrm{Ala}$ substitutions probe the effect of replacing the conserved glycine position with a larger residue.^20,71^ As described in Methods, these mutations were introduced into tripeptides that remained undeformed in the control simulations, allowing their effects to be distinguished from those of CMP instability.

We find that d-glucose concentration does not affect interstrand or CMP-water HBs in nonmutated (PPG)_12_ CMPs (Figure S20). However, we see that the introduction of Ala and Lys breaks interstrand HBs at or near the mutation site (Figures S21–24). At the Ala mutation sites, CMP-water HBs exhibit a greater standard deviation but remain, on average, of the same magnitude as those in the nonmutated (PPG)_12_. The average number of hydration waters also remains statistically similar to that of the nonmutated tripeptides. This indicates that the presence of Ala does not significantly affect the hydration structure. In contrast, we see between 6 and 10 CMP-water HBs near the Lys mutation sites, which is a significant increase from the 3 to 5 HBs seen for nonmutated tripeptides. This observation holds true for all d-glucose concentrations. At the same time, the number of hydration waters around the Lys mutation site shows a dip from 5 to 10 to 2–4, which can be attributed to steric exclusion caused by the bulkier side chain. In effect, the side chain creates a local environment where fewer water molecules can occupy the hydration shell, but those that do are more likely to form HBs. Importantly, we observed these features regardless of the position of the mutation. This indicates that these effects are due to the chemical nature of the Lys substitution.

These HB disruptions result in varying levels of intrastrand and interstrand deformations, as quantified by ⟨κ^*i*,*m*^(*t*)⟩_*m*,*t*_ and ⟨ζ^*i*^(*t*)⟩_*t*_. For (PPG)_12_ CMP, the first four tripeptides exhibit statistically similar levels of intrastrand deformation at all concentrations (Figure S25). For the interstrand deformation, however, ⟨ζ^*i*^(*t*)⟩_*t*_ for tripeptide #1 increases from 2.2 at 0 mM to 5.5 at 2000 mM. Similarly, there is greater interstrand deformation for tripeptide #2 when d-glucose is present. For all other tripeptides, ⟨ζ^*i*^(*t*)⟩_*t*_ ≤ 1.

Interestingly, we observe the opposite effect in the Ala-mutated (PPG)_12_ CMPs (Figure S26). Indeed, for $\mathrm{Gly}\overset{\mathrm{end}}{\to }\mathrm{Ala}$ , ⟨κ^*i*,*m*^(*t*)⟩_*m*,*t*_ at tripeptides #11 and #12 decreases from 2.0 ± 0.5 at 0 mM to below 1 in the presence of d-glucose. For $\mathrm{Gly}\overset{\mathrm{mid}}{\to }\mathrm{Ala}$ , ⟨κ^*i*,*m*^(*t*)⟩_*m*,*t*_ is ≥ 1 near the mutation site for all concentrations, but ⟨ζ^*i*^(*t*)⟩_*t*_ at tripeptide #5 decreases from 1.5 ± 0.5 at 0 mM to 0.6 ± 0.2 at 2000 mM. The d-glucose-dependent deformation at the Ala mutation site can be seen visually in Figure 7. Indeed, the CMP only bends into an L-shape in the absence of d-glucose. This is also supported by the end-to-end distances of $\mathrm{Gly}\overset{\mathrm{end}}{\to }\mathrm{Ala}$ and $\mathrm{Gly}\overset{\mathrm{mid}}{\to }\mathrm{Ala}$ CMPs, which only decrease significantly at 0 mM (Figures S27a and b). For $\mathrm{Pro}\overset{\mathrm{end}}{\to }\mathrm{Lys}$ and $\mathrm{Pro}\overset{\mathrm{mid}}{\to }\mathrm{Lys}$ , both κ^*i*,*m*^(*t*)⟩_*m*,*t*_ ≤ 1 and ⟨ζ^*i*^(*t*)⟩_*t*_ ≤ 1 at the mutated tripeptides for all concentrations of d-glucose. This means that the presence of Lys does not cause intra- or interstrand deformations. No bending is observed for these CMPs either, as shown by their end-to-end distances (Figures S27c and d).

**Figure 7:**
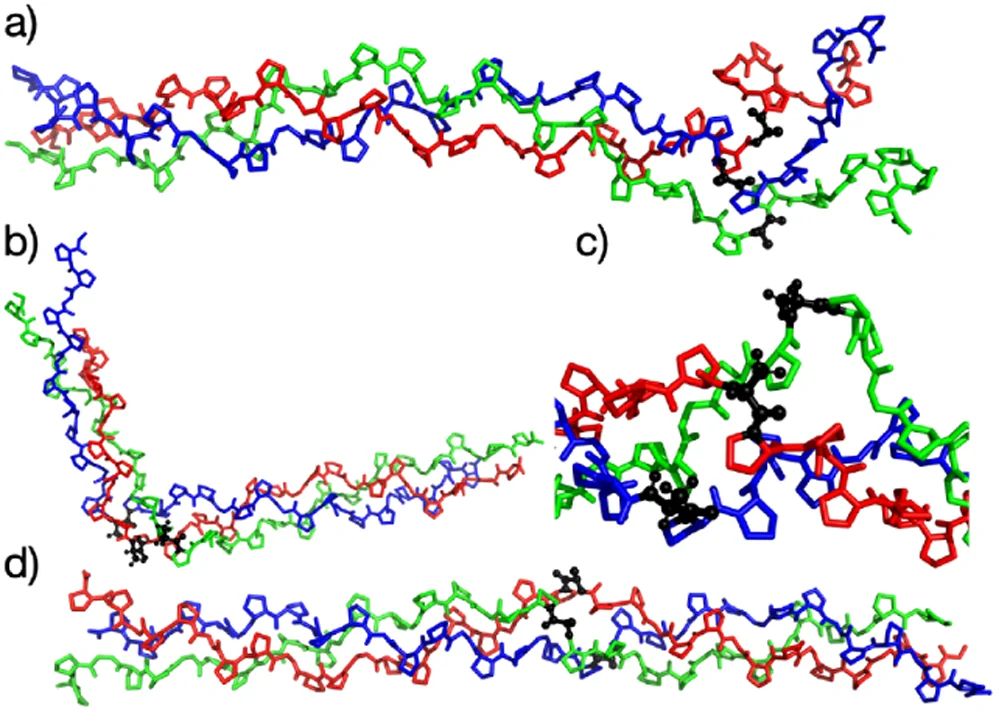
Structural deformation in the triple helix due to a Gly → Ala mutation (black) in AMOEBA. Water and d-glucose molecules are omitted for clarity. a) $\mathrm{Gly}\overset{\mathrm{end}}{\to }\mathrm{Ala}$ with 0 mM d-glucose at 95 ns. b) $\mathrm{Gly}\overset{\mathrm{mid}}{\to }\mathrm{Ala}$ with 0 mM d-glucose at 95 ns. c) The mutated site for $\mathrm{Gly}\overset{\mathrm{mid}}{\to }\mathrm{Ala}$ from (b), enlarged. d) $\mathrm{Gly}\overset{\mathrm{mid}}{\to }\mathrm{Ala}$ with 2000 mM d-glucose at 95 ns.

Thus, far, the effect of d-glucose has been seen to be system-specific and localized at the N-terminus and/or mutation sites. However, these localized disruptions do not threaten the structural integrity of the triple helix. In fact, we observe the opposite in the correlation between ⟨κ^*i*,*m*^(*t*)⟩_*i*,*m*_ and ⟨ζ^*i*^(*t*)⟩_*i*_ (Figures S24–S28). In this case, the two populations identified in Movie S1 are seen across all d-glucose concentrations. However, the fractional size of the second population decreases from 5.13% at 0 mM to 3.12%, 2.99%, and 0.07% for (PPG)_12_ at 500 mM, 1000 mM, and 2000 mM, respectively (Figure S28). We observe similar trends for $\mathrm{Gly}\overset{\mathrm{mid}}{\to }\mathrm{Ala}$ (Figure S29), $\mathrm{Gly}\overset{\mathrm{end}}{\to }\mathrm{Ala}$ (Figure S30), $\mathrm{Pro}\overset{\mathrm{end}}{\to }\mathrm{Lys}$ (Figure S31), and $\mathrm{Pro}\overset{\mathrm{mid}}{\to }\mathrm{Lys}$ (Figure S32). This means that the presence of glucose prevents the strands from moving apart, resulting in lower instances of high ⟨ζ^*i*^(*t*)⟩_*i*_.

Our results indicate that d-glucose indirectly stabilizes all CMPs. Indeed, the RDF in Figure S33 shows that d-glucose does not stay in close proximity to the CMPs, and therefore does not directly interact with the peptide for extended periods of time. The longest residence time is about 4 ns at 2000 mM for the nonmutated and Ala-mutated (PPG)_12_ CMPs (Table S6). However, we note that the maximum residence time rises to 5.1 and 5.7 ns at 2000 mM for $\mathrm{Pro}\overset{\mathrm{end}}{\to }\mathrm{Lys}$ and $\mathrm{Pro}\overset{\mathrm{mid}}{\to }\mathrm{Lys}$ , respectively. This is due to the ${\mathrm{NH}}_{3}^{+}$ group of the Lys side chain (Table S6), which provides a localized favorable hotspot. Functionally, Lys increases the probability of prolonged contact and raises the chances of nonenzymatic glycation. In our simulations, d-glucose decreases the available space for the triple helix, thus keeping the strands physically proximate. Our results from AMOEBA compare well with recent experimental studies on CMPs with d-glucose and trehalose, which show that saccharides overall stabilize the collagen triple helix by a common mechanism, independent of saccharide structure, by increasing the melting temperature of the triple helix.^137,138^

Finally, we note that the magnitude of $D_{\mathrm{iso}}^{\mathrm{trans}}$ and $D_{\mathrm{iso}}^{\mathrm{rot}}$ do not change with d-glucose concentration or mutation (Table S6). In all cases, $D_{\mathrm{zz}}^{\mathrm{trans}}$ and $D_{r_{3}r_{3}}^{\mathrm{rot}}$ are also consistently lower than the other components for all mutated CMPs, just like in (PPG)_12_. This persistent trend across all mutations and d-glucose concentrations implies that neither d-glucose nor the presence of a mutation changes the directional preference in hydration water mobility. In the absence of d-glucose, $c_{\mathrm{aniso}}^{\mathrm{trans}}=0.31$ for the Ala-mutated CMPs and 0.36–0.39 for the Lys-mutated CMPs, compared to 0.44 for (PPG)_12_. It is likely that $c_{\mathrm{aniso}}^{\mathrm{trans}}$ decreases more for Ala-mutated CMPs because they bend, which disrupts the elongation in the **z**-direction. $c_{\mathrm{aniso}}^{\mathrm{trans}}$ drops dramatically for all CMPs at 500 mM before increasing again at 1000 and 2000 mM. Importantly, we see that $c_{\mathrm{aniso}}^{\mathrm{trans}}$ behaves similarly for (PPG)_12_ and the Lys-mutated CMPs and that it is independent of the position of the mutation. This implies that $c_{\mathrm{aniso}}^{\mathrm{trans}}$ is a characteristic of the integrity of the triple helix, rather than a characteristic of its chemical nature. Overall, we expect greater anisotropy in translational kinetics in healthy CMPs. In contrast, $c_{\mathrm{aniso}}^{\mathrm{rot}}$ stays constant at 0.31–0.32 for all CMPs, except $\mathrm{Gly}\overset{\mathrm{end}}{\to }\mathrm{Ala}$ , where it decreases to 0.08. We suspect this is an outlier, probably due to high interstrand deformation at the four C-terminus tripeptides. Similarly, with a couple of exceptions, d-glucose concentration does not significantly affect $c_{\mathrm{aniso}}^{\mathrm{rot}}$ . This is because water rotation is a more localized motion than translation and therefore is less likely to be impacted by global changes in the environment.

## Conclusion

In this work, we have carried out a systematic investigation of (PPG)_*n*_ CMPs using the AMOEBA force field. We introduce new structural metrics and provide an analysis of hydration dynamics as a function of CMP length, sequence, and solvent composition. We find that AMOEBA is well-suited to predict pyrrolidine ring puckering, similar to Cm4P and A4P. Further, we find that AMOEBA, and, to a lower extent, A4P captures the experimentally observed length-dependent deformation of CMPs, a feature not reproduced by C3P, C4P, or Cm4P. Compared to nonpolarizable force fields, AMOEBA is also able to better describe noncovalent *n* → π* interactions, which act competitively with interstrand hydrogen bonding and are typically only described at the quantum level. Our new metrics, κ^*i*,*m*^(*t*) and ζ^*i*^(*t*) differentiate between deformations arising from bending, stretching, or strand separation, providing information not previously available. Note that these metrics are not specific to (PPG)_*n*_ CMPs and can serve in future computational studies of arbitrary multistrand helices.

We also demonstrate that water molecules around CMPs exhibit both translational and rotational anisotropies that are not present in bulk water. These findings highlight the link between triple-helix conformation and hydration dynamics and suggest that anisotropic water diffusion may serve as a sensitive molecular signature of ordered collagen-like CMP structures.

Finally, we examined how mutations modulate the structure and local environment of the CMP triple helix. Gly → Ala substitutions produced local structural disruptions in the triple helix, whereas Lys substitutions introduced additional interactions with water and d-glucose through the ${\mathrm{NH}}_{3}^{+}$ side chain. We found that d-glucose exhibits long-lived residence times near Lys, slowing down relative to bulk motion and generating local environments that may be favorable for early steps of nonenzymatic glycation chemistry.

Overall, this work provides a controlled molecular perspective on how hydration, local sequence changes, and sugar interactions influence collagen-like triple-helical structures. While (PPG)_*n*_ CMPs do not reproduce the full sequence diversity, chemical complexity, or hierarchical organization of native collagen, they offer useful fundamental models for isolating molecular mechanisms that may be relevant to collagen stability and glycation-related structural changes. Future extensions incorporating more native-like sequences, post-translational modifications, and complex extracellular environments will help connect these atomistic insights to broader biological and biomedical contexts.

## Supplementary Material



## Data Availability

The data and codes that support the findings of this study are available by clicking here (DOI:10.5281/zenodo.18330925). HeliXplore is also archived and available by clicking here (DOI:10.5281/zenodo.18331481).

## Notes

Mathematical derivation of **Q** ^*w*^(*t*) and **ω** ^*w*^(*t*) and additional simulation data, as follows: Metrics to check for interaction between CMP and periodic images for (PPG)_12_ and (PPG)_12_ in AMOEBA. ⟨κ^i,m^(t)⟩_m,t_ and ⟨ζ^i^(t)⟩_t_ for (PPG)_12_ for the AMOEBA simulations and simulations with the sc remove-inertia keyword in TINKER. Backbone RMSD of (PPG)_*n*_ CMPs over time in C3P, C4P, Cm4P, A4P, and AMOEBA. RDF of the (PPG)_*n*_ CMPs with water and sc d-glucose from in AMOEBA. Mean dihedral angles (ϕ, ψ, χ, and ω) and Ramachandran plots from C3P, C4P, Cm4P, A4P, and AMOEBA. *n* → π* interaction conditions for (PPG)_12_ and (PPG)_25_. δ_θ_(*t*), s­(*t*), ⟨κ^i,m^(t)⟩_m_, and ζ^i^(t) over time to accompany Figures 4 and 5. (PPG)_*n*_ snapshots to accompany Figure 5. Interstrand HBs for (PPG)_*n*_ without sc d-glucose in AMOEBA. Interstrand HBs, CMP-water HBs, and hydration waters for each tripeptide for (PPG)_*n*_ and singly mutated CMPs in AMOEBA. Distribution of hydration water residence times for (PPG)_*n*_ without sc d-glucose in AMOEBA. Decay of Tr­(**D** ^trans^(t)) and $\frac{1}{2}\left(3{\left(\mathrm{Tr}(D^{\mathrm{rot}}\left(t\right))\right)}^{2}-1\right)$ for the waters in the first hydration shell of (PPG)_n_. ⟨κ^i,m^(t)⟩_m,t_ and ⟨ζ^i^(t)⟩_t_ for (PPG)_12_ and singly mutated CMPs with sc d-glucose over the tripeptide index. Correlation of ⟨κ^i,m^(t)⟩_m,t_ and ⟨ζ^i^(t)⟩_t_ for (PPG)_12_ and singly mutated CMPs with sc d -glucose. End-to-end distances over time for singly mutated CMPs with sc d-glucose. Diagonal elements of *D* ^trans^ and *D* ^rot^ for (PPG)_12_ and the singly mutated CMPs in sc d-glucose, along with $D_{iso}^{\mathrm{trans}}$ , $D_{iso}^{\mathrm{rot}}$ , $c_{\mathrm{aniso}}^{\mathrm{trans}}$ , $c_{\mathrm{aniso}}^{\mathrm{rot}}$ . RDF of sc d-glucose with Lys ${\mathrm{NH}}_{3}^{+}$ for Pro→Lys CMPs from AMOEBA. $k_{g}^{\mathrm{ex}}$ and maximum L^g^(e) for sc d-glucose for (PPG)_12_ and the singly mutated CMPs.(PDF)
