# Supplementary material for: Using AMOEBA to Uncover Deformation and Hydration Anisotropy in Collagen-Mimetic Peptides
Source: J Phys Chem B. 2026 Sep 4;130(37):9481–95. doi: 10.1021/acs.jpcb.6c02718 (PMC13592025; doi:10.1021/acs.jpcb.6c02718)
Supplement: Supplementary file 1 [file jp6c02718_si_001.pdf]

Using AMOEBA to Uncover Deformation and Hydration Anisotropy in Collagen Mimetic Peptides

Supplementary Information

Ronnie Mondal[1,2], Valerie Vaissier Welborn*[1,2]

1. Department of Chemistry, Virginia Tech, Blacksburg, Virginia, USA 24061

2. Macromolecules Innovation Institute, Virginia Tech, Blacksburg, Virginia, USA 24061

*vwelborn@vt.edu

August 12, 2026

The components of unit quaternion $\mathbf{Q}^w(t)$ are computed from the components of $\mathbf{R}^w(t)$ using Shepperd's algorithm.[1,2] There are four numerically stable formulae depending on $\mathrm{Tr}(\mathbf{R}^w(t)) = r^w_{1x}(t) + r^w_{2y}(t) + r^w_{3z}(t)$ and the individual diagonal elements $r^w_{1x}(t)$, $r^w_{2y}(t)$ and $r^w_{3z}(t)$.

Case A: If $\mathrm{Tr}(\mathbf{R}^w(t)) > 0$ (the most common case), then

$$q^w_0(t) = \tfrac{1}{2}\sqrt{r^w_{1x}(t) + r^w_{2y}(t) + r^w_{3z}(t) + 1} \tag{1a}$$

$$q^w_1(t) = \frac{r^w_{3y}(t) - r^w_{2z}(t)}{2\sqrt{r^w_{1x}(t) + r^w_{2y}(t) + r^w_{3z}(t) + 1}} \tag{1b}$$

$$q^w_2(t) = \frac{r^w_{1z}(t) - r^w_{3x}(t)}{2\sqrt{r^w_{1x}(t) + r^w_{2y}(t) + r^w_{3z}(t) + 1}} \tag{1c}$$

$$q^w_3(t) = \frac{r^w_{2x}(t) - r^w_{1y}(t)}{2\sqrt{r^w_{1x}(t) + r^w_{2y}(t) + r^w_{3z}(t) + 1}} \tag{1d}$$

Case B: If $\mathrm{Tr}(\mathbf{R}^w(t)) \leq 0$ and $r^w_{1x}(t)$ is the largest diagonal element, then

$$q^w_0(t) = \frac{r^w_{3y}(t) - r^w_{2z}(t)}{2\sqrt{1 + r^w_{1x}(t) - r^w_{2y}(t) - r^w_{3z}(t)}} \tag{2a}$$

$$q^w_1(t) = \tfrac{1}{2}\sqrt{1 + r^w_{1x}(t) - r^w_{2y}(t) - r^w_{3z}(t)} \tag{2b}$$

$$q^w_2(t) = \frac{r^w_{1y}(t) + r^w_{2x}(t)}{2\sqrt{1 + r^w_{1x}(t) - r^w_{2y}(t) - r^w_{3z}(t)}} \tag{2c}$$

$$q^w_3(t) = \frac{r^w_{1z}(t) + r^w_{3x}(t)}{2\sqrt{1 + r^w_{1x}(t) - r^w_{2y}(t) - r^w_{3z}(t)}} \tag{2d}$$

Case C: If $\mathrm{Tr}(\mathbf{R}^w(t)) \leq 0$ and $r^w_{2y}(t)$ is the largest diagonal element, then

$$q^w_0(t) = \frac{r^w_{1z}(t) - r^w_{3x}(t)}{2\sqrt{1 + r^w_{2y}(t) - r^w_{1x}(t) - r^w_{3z}(t)}} \tag{3a}$$

$$q^w_1(t) = \frac{r^w_{1y}(t) + r^w_{2x}(t)}{2\sqrt{1 + r^w_{2y}(t) - r^w_{1x}(t) - r^w_{3z}(t)}} \tag{3b}$$

$$q^w_2(t) = \tfrac{1}{2}\sqrt{1 + r^w_{2y}(t) - r^w_{1x}(t) - r^w_{3z}(t)} \tag{3c}$$

$$q^w_3(t) = \frac{r^w_{2z}(t) + r^w_{3y}(t)}{2\sqrt{1 + r^w_{2y}(t) - r^w_{1x}(t) - r^w_{3z}(t)}} \tag{3d}$$

Case D: If $\mathrm{Tr}(\mathbf{R}^w(t)) \leq 0$ and $r^w_{3z}(t)$ is the largest diagonal element, then

$$q^w_0(t) = \frac{r^w_{2x}(t) - r^w_{1y}(t)}{2\sqrt{1 + r^w_{3z}(t) - r^w_{1x}(t) - r^w_{2y}(t)}} \tag{4a}$$

$$q^w_1(t) = \frac{r^w_{1z}(t) + r^w_{3x}(t)}{2\sqrt{1 + r^w_{3z}(t) - r^w_{1x}(t) - r^w_{2y}(t)}} \tag{4b}$$

$$q^w_2(t) = \frac{r^w_{2z}(t) + r^w_{3y}(t)}{2\sqrt{1 + r^w_{3z}(t) - r^w_{1x}(t) - r^w_{2y}(t)}} \tag{4c}$$

$$q^w_3(t) = \tfrac{1}{2}\sqrt{1 + r^w_{3z}(t) - r^w_{1x}(t) - r^w_{2y}(t)} \tag{4d}$$

A tiny perturbation of 10^{-10} was added to the denominators if $\leq 10^{-10}$ to prevent numerical instabilities.

The components of the angular velocity $\boldsymbol{\omega}^w(t)$were computed from the component of the unit quaternion $\mathbf{Q}^w(t)$ as:

$$\begin{bmatrix}\omega^w_{r_1}(t)\\ \omega^w_{r_2}(t)\\ \omega^w_{r_3}(t)\end{bmatrix} = 2\,\mathrm{Im}\left((\mathbf{Q}^w(t))^* \frac{\partial \mathbf{Q}^w(t)}{\partial t}\right) = 2\begin{bmatrix} q^w_0(t)\frac{\partial q^w_1(t)}{\partial t} - q^w_1(t)\frac{\partial q^w_0(t)}{\partial t} - q^w_2(t)\frac{\partial q^w_3(t)}{\partial t} + q^w_3(t)\frac{\partial q^w_2(t)}{\partial t}\\ q^w_0(t)\frac{\partial q^w_2(t)}{\partial t} - q^w_2(t)\frac{\partial q^w_0(t)}{\partial t} - q^w_3(t)\frac{\partial q^w_1(t)}{\partial t} + q^w_1(t)\frac{\partial q^w_3(t)}{\partial t}\\ q^w_0(t)\frac{\partial q^w_3(t)}{\partial t} - q^w_3(t)\frac{\partial q^w_0(t)}{\partial t} - q^w_1(t)\frac{\partial q^w_2(t)}{\partial t} + q^w_2(t)\frac{\partial q^w_1(t)}{\partial t}\end{bmatrix} \quad (5)$$

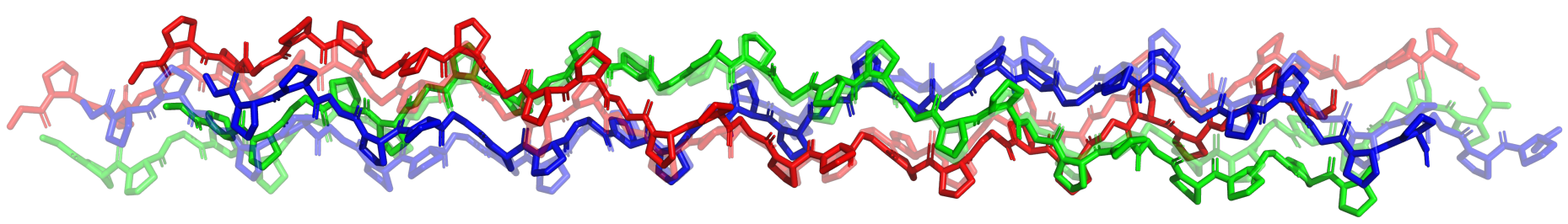

Figure S1: Superposition of the THeBuScr-generated $(PPG)_{12}$ structure and the experimentally determined $(PPG)_{10}$ structure. We count 10 Gly(NH)-Xaa(CO) hydrogen bonds in $(PPG)_{10}$ compared to 12 in $(PPG)_{12}$; and 2 Xaa(C-alpha)-CO hydrogen bonds in $(PPG)_{10}$ compared to 3 in $(PPG)_{12}$. The axial shift is 5.6 Å with $\delta_\theta = 0$ in $(PPG)_{10}$ compared to 6.1 Å with $\delta_\theta = 0$ in $(PPG)_{12}$.

**Angle**	**Residue**	**$(PPG)_{12}$** From THeBuScr[3]	**$(PPG)_{10}$** From Exp.[4]
ϕ	Pro(Xaa)	-74	-75
	Pro(Yaa)	-60	-60
	Gly	-71	-72
ψ	Pro(Xaa)	161	164
	Pro(Yaa)	150	152
	Gly	174	176
ω	All	179	177
χ	Exo:Endo (Xaa)	50:50	50:50
	Exo:Endo (Yaa)	75:25	50:50
	Endo	33	31
	Exo	-31	-30

Table S1: Geometric comparison between the THeBuScr-generated $(PPG)_{12}$ structure and the experimentally determined $(PPG)_{10}$ structure (both shown in Figure S3). THeBuScr-generated structures are used as initial configurations for the MD simulations in this work.

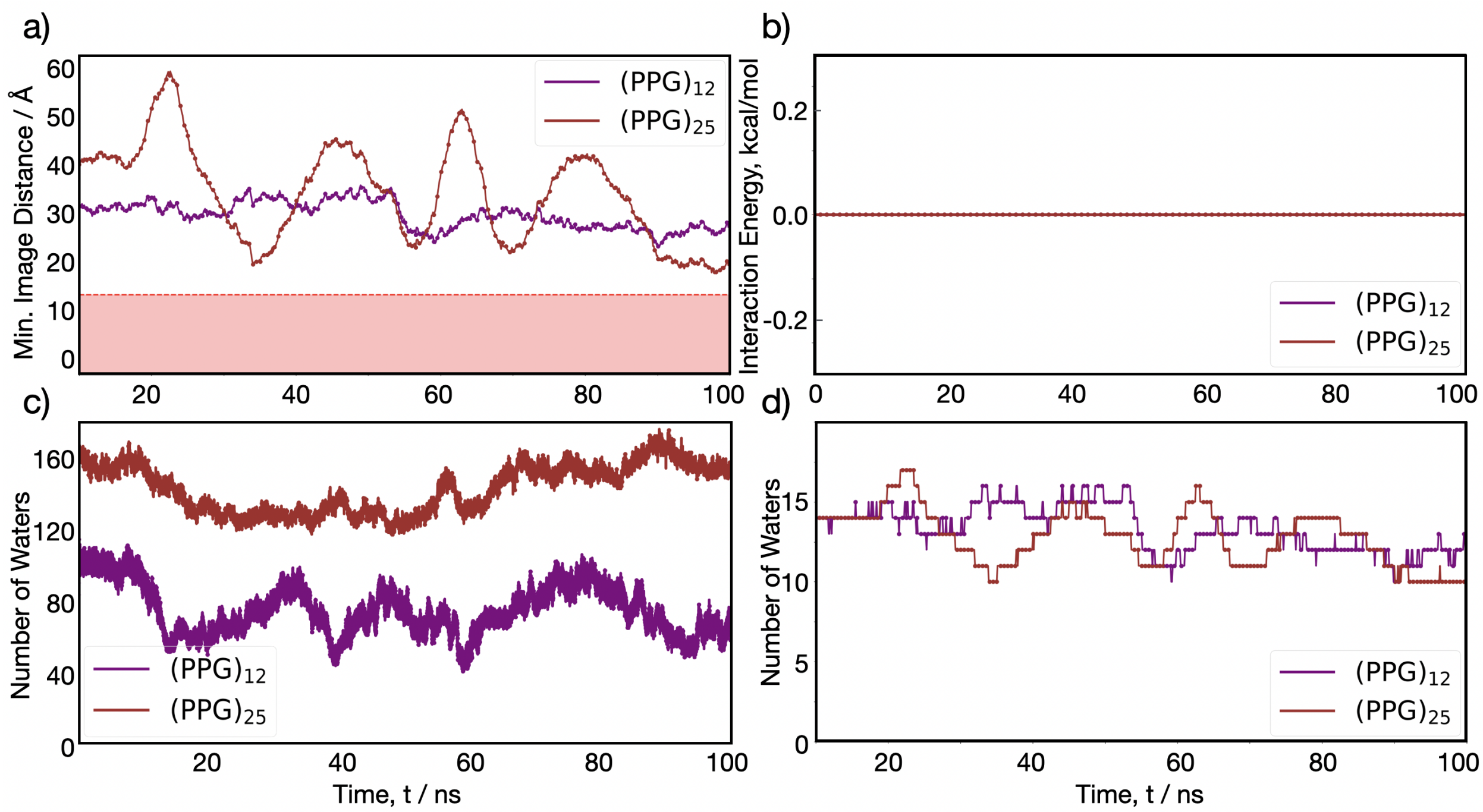

Figure S2: a) Minimum distance between a non-H atom in the main box with another non-H atom in the periodic image in $\pm x$ and $\pm y$ directions for $(PPG)_{12}$ and $(PPG)_{25}$ in AMOEBA. The red region shows a 15 Å cutoff considered for interaction. b) Total interaction energy between the CMP and its periodic image in $\pm x$ and $\pm y$ directions. Here, the interaction energy is calculated using Tinker9 ANALYZE[5] as the sum of van der Waals, permanent and induced electrostatics between all unique unit cell-image (in the $\pm x$ and $\pm y$ directions) atom pairs. c) The total number of water molecules within 3.5 Å of the CMP in the main box. d) The number of water molecules along the minimum image distance between the most proximate non-H atoms in the $\pm x$ and $\pm y$ periodic images.

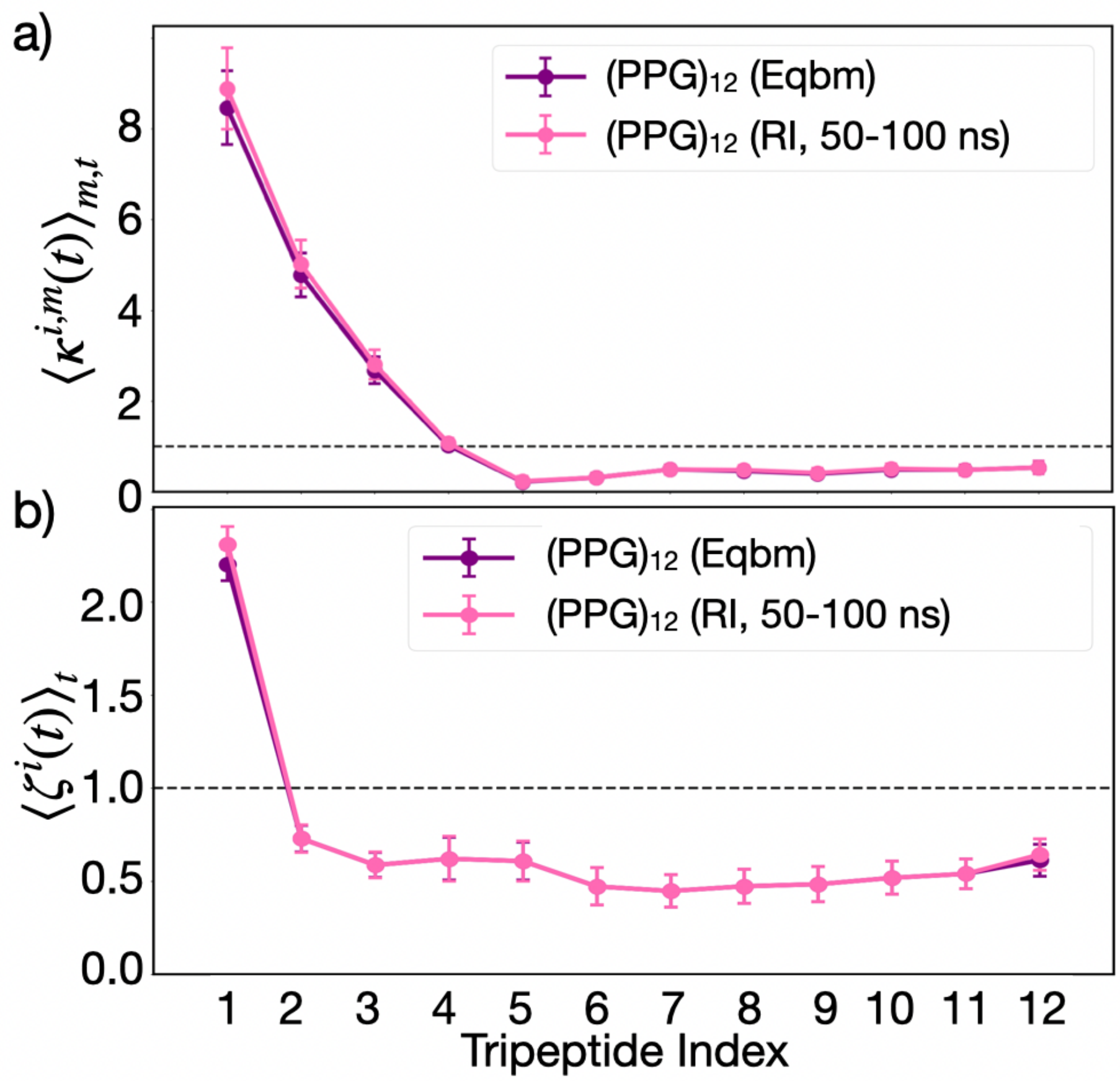

Figure S3: a) Intra-strand ($\langle \kappa^{i,m}(t) \rangle_{m,t}$) and b) inter-strand ($\langle \zeta^{i}(t) \rangle_{t}$) deformation for each tripeptide in $(PPG)_{12}$ with AMOEBA. The values marked as "Eqbm." are used in the manuscript and are averaged over the 50-100 ns time frames. The values marked as "RI" are the control simulations run removing the linear and angular momenta at each time step of the simulations.

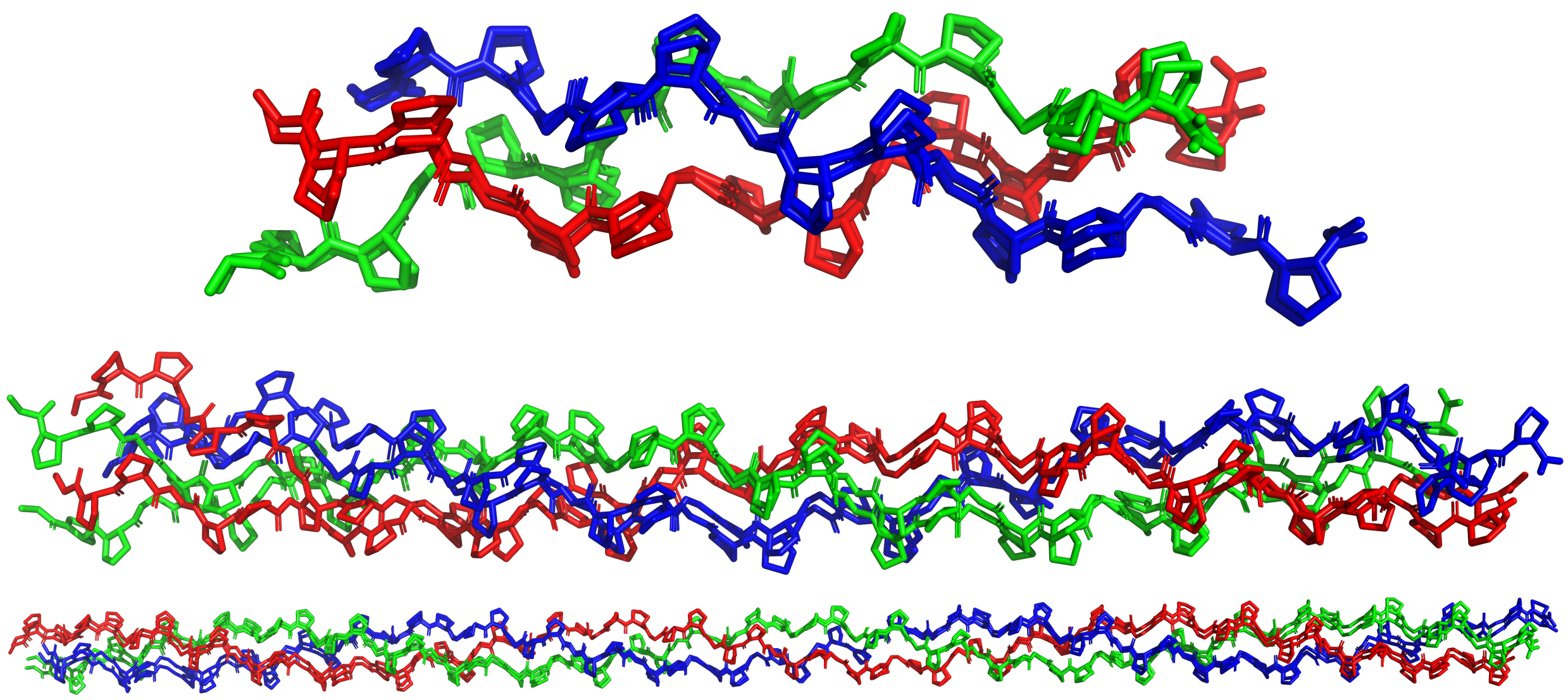

Figure S4: Superposition of the initial and final CMP geometries from the C3P MD simulations of $(PPG)_5$ (top), $(PPG)_{12}$ (middle), and $(PPG)_{25}$ (bottom). The corresponding RMSD values are 1.98, 2.19, and 3.98 Å, respectively. For $(PPG)_5$ and $(PPG)_{12}$, both geometries were used as starting structures for the AMOEBA simulations, and the results are independent of this choice (Figures S4–S6).

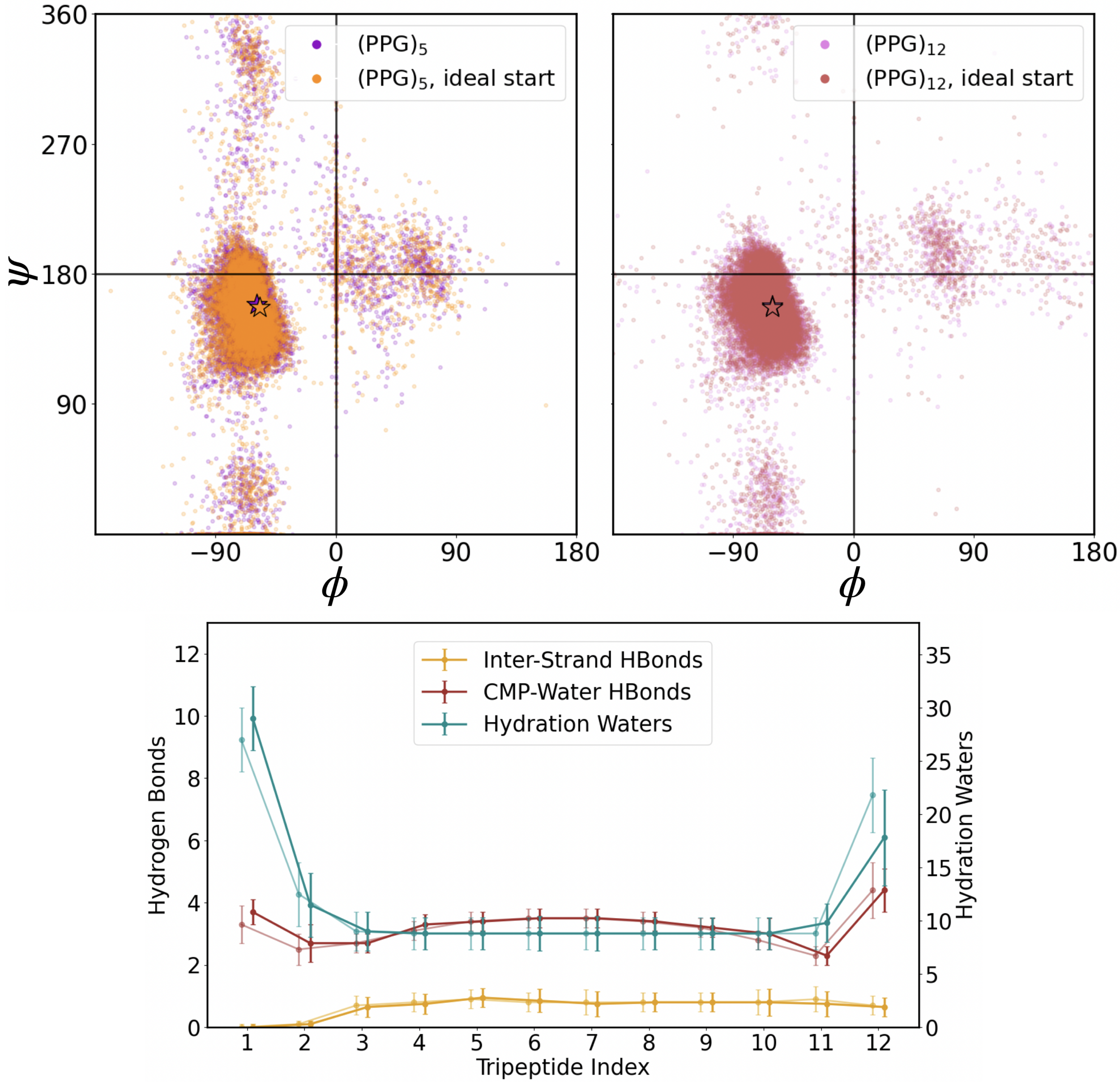

Figure S5: Top: Ramachandran plots for $(PPG)_5$ and $(PPG)_{12}$ in AMOEBA simulations starting from the ideal triple helix geometry generated by the BuSCR script and starting from the final frame of the C3P simulations. Bottom: Hydrogen bonds in the AMOEBA simulations starting from the ideal triple helix geometry generated by the BuSCR script (light lines) and starting from the final frame of the C3P simulations (dark lines). Note that the data points correspond to integer values of the tripeptide index but have been slightly offset to improve readability.

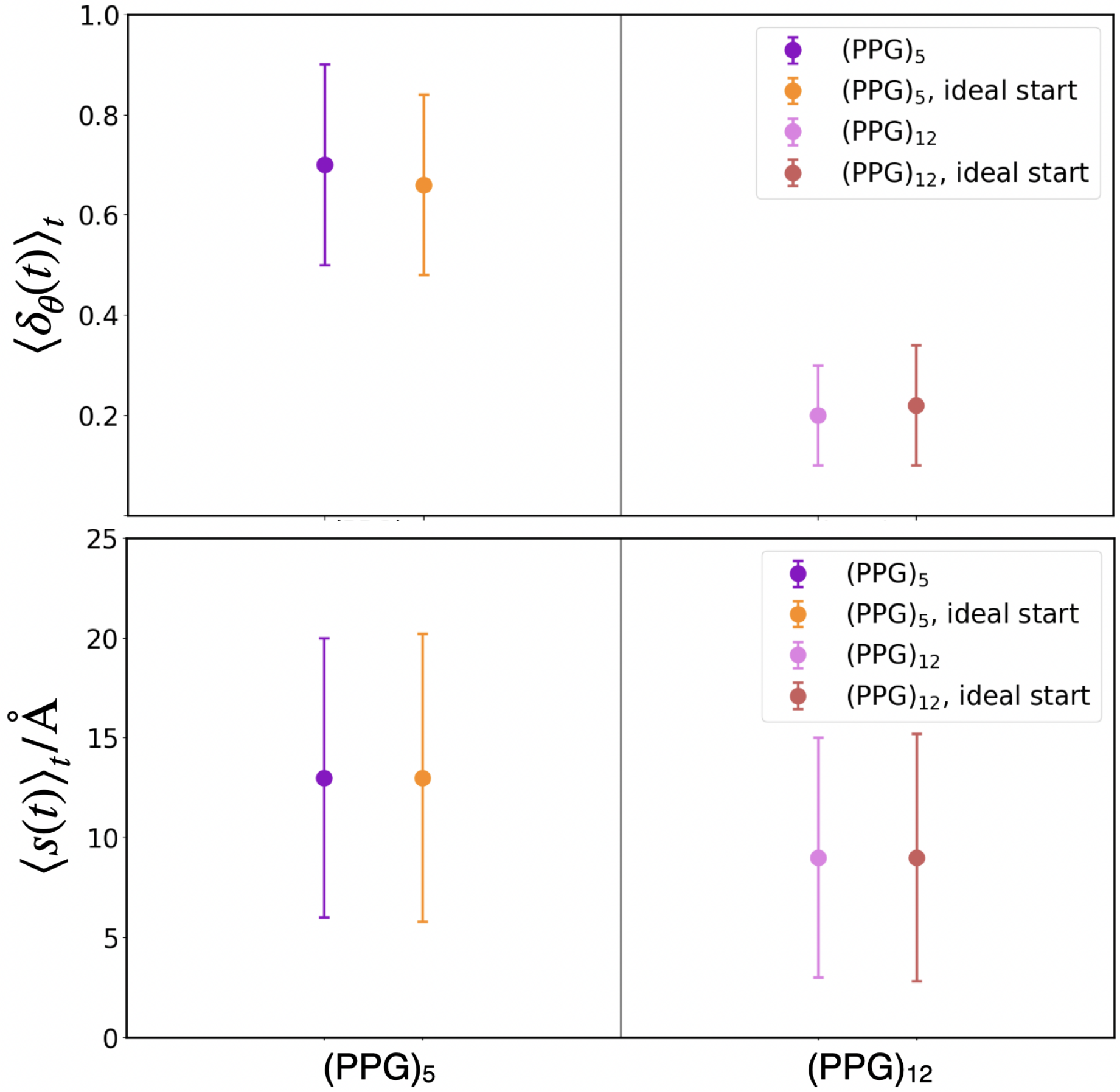

Figure S6: $\langle \delta_\theta(t) \rangle_t$ and average axial shift the AMOEBA simulations starting from the ideal triple helix geometry generated by the BuSCR script and starting from the final frame of the C3P simulations.

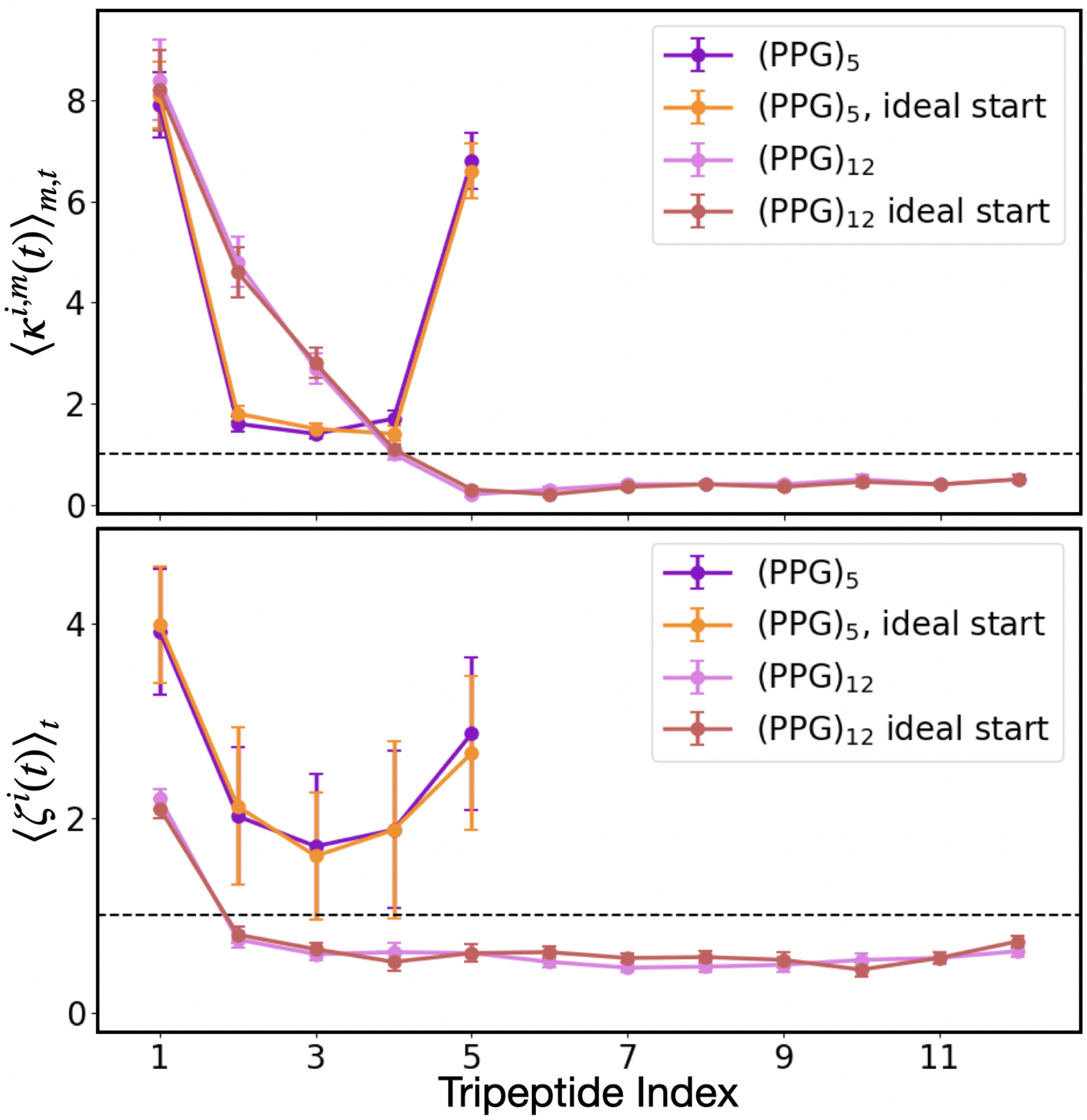

Figure S7: $\langle \kappa^{i,m}(t) \rangle_{m,t}$ for $(PPG)_5$ and $(PPG)_{12}$ over tripeptide index in the AMOEBA simulations starting from the ideal triple helix geometry generated by the BuSCR script and starting from the final frame of the C3P simulations. g) $\langle \zeta^{i}(t) \rangle_{t}$ for $PPG)_5$ and $(PPG)_{12}$ over tripeptide index in the AMOEBA simulations starting from the ideal triple helix geometry generated by the BuSCR script and starting from the final frame of the C3P simulations..

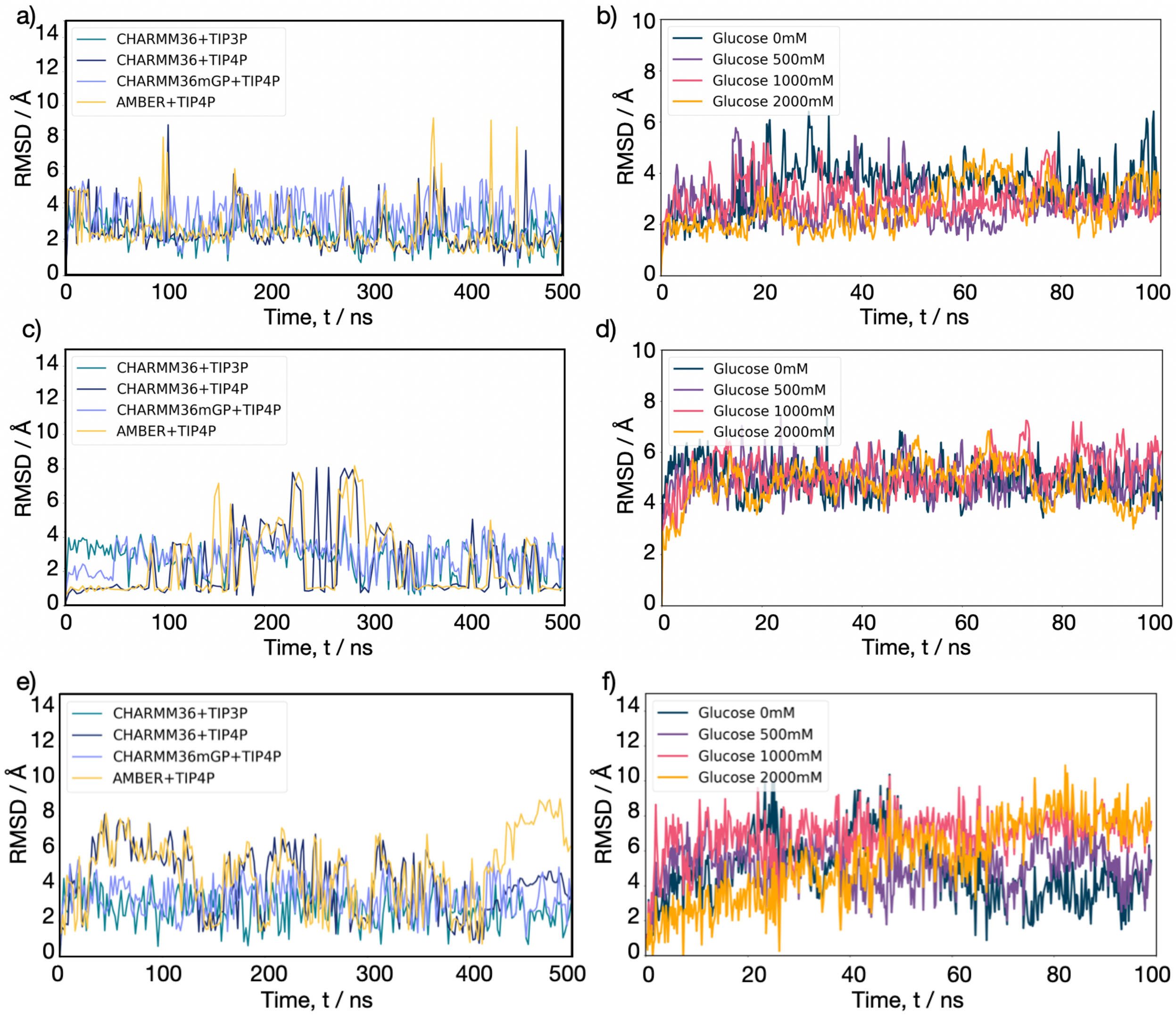

Figure S8: Backbone RMSD for the protein over the MD simulation time. a) $(PPG)_5$ with no D-glucose in CHARMM36+TIP3P, CHARMM36+TIP4P, CHARMM36mGP+TIP4P and AMBER+TIP4P. b) $(PPG)_5$ with AMOEBA. c) $(PPG)_{12}$ with no D-glucose in CHARMM36+TIP3P, CHARMM+TIP4P, CHARMM36mGP+TIP4P and AMBER+TIP4P. d) $(PPG)_{12}$ with AMOEBA. e) $(PPG)_{25}$ with no D-glucose in CHARMM36+TIP3P, CHARMM+TIP4P, CHARMM36mGP+TIP4P and AMBER+TIP4P. f) $(PPG)_{25}$ with AMOEBA.

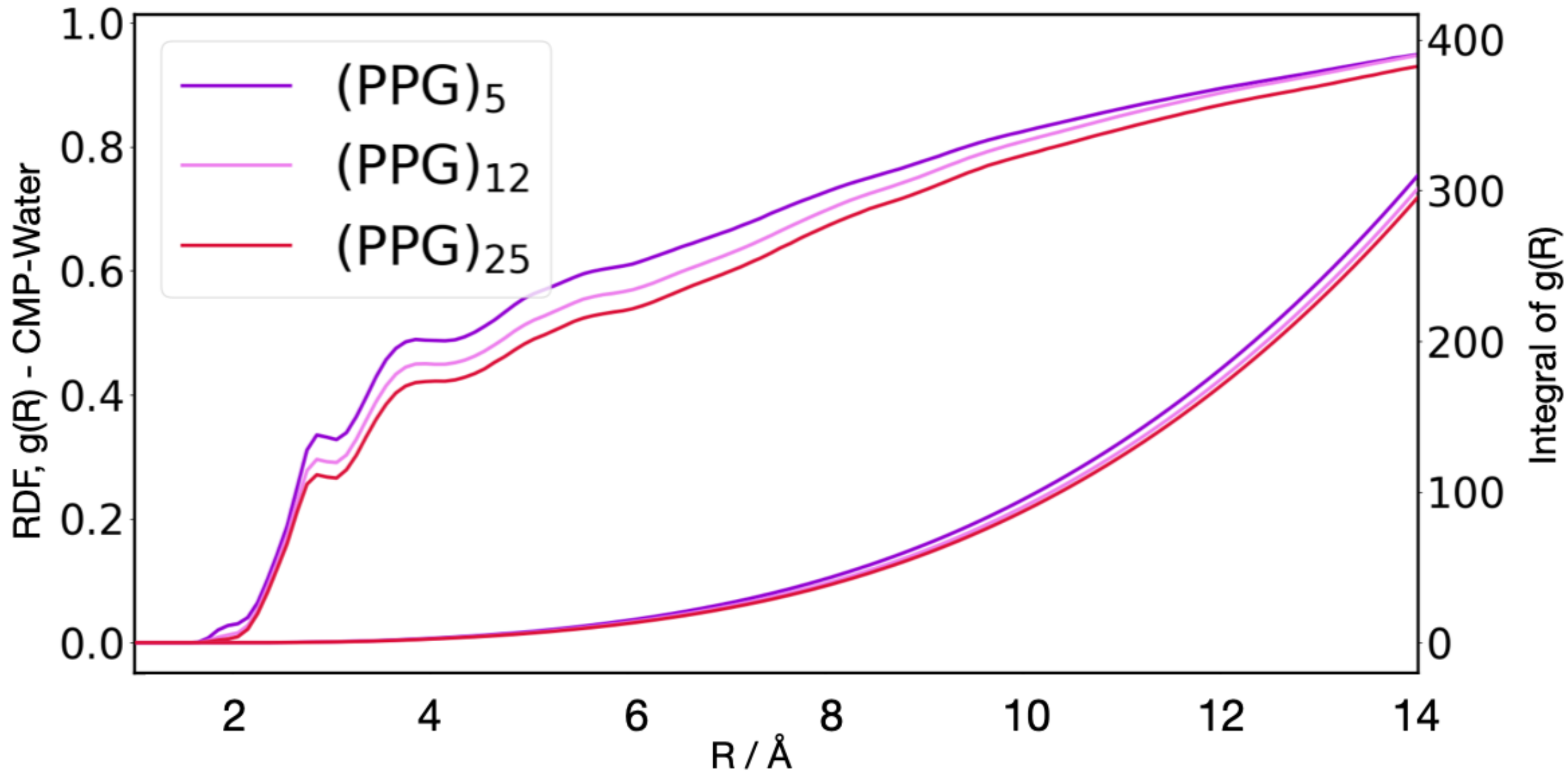

Figure S9: RDF of the (PPG)$_n$ CMPs with water in AMOEBA, used to calculate the extent of the first hydration shell (3.5 Å).

Conformational flexibility of $(PPG)_n$ CMP strands

Angle	Force Field	Residue	$(PPG)_5$	$(PPG)_{12}$	$(PPG)_{25}$
ϕ	CHARMM36+TIP3P	Pro(Xaa)	-54 ± 13	-55 ± 13	-56 ± 14
		Pro(Yaa)	-46 ± 9	-45 ± 9	-45 ± 9
		Gly	-58 ± 9	-60 ± 11	-60 ± 13
	CHARMM36+TIP4P	Pro(Xaa)	-64 ± 11	-64 ± 12	-66 ± 11
		Pro(Yaa)	-58 ± 14	-60 ± 10	-59 ± 10
		Gly	-60 ± 12	-61 ± 13	-62 ± 15
	CHARMM36mGP+TIP4P	Pro(Xaa)	-68 ± 16	-65 ± 12	-67 ± 11
		Pro(Yaa)	-55 ± 14	-58 ± 10	-60 ± 10
		Gly	-65 ± 14	-66 ± 13	-65 ± 11
	AMBER+TIP4P	Pro(Xaa)	-65 ± 13	-71 ± 16	-72 ± 16
		Pro(Yaa)	-65 ± 13	-72 ± 16	-72 ± 16
		Gly	-54 ± 14	-60 ± 17	-61 ± 16
	AMOEBA	Pro(Xaa)	-56 ± 37	-62 ± 25	-66 ± 21
		Pro(Yaa)	-58 ± 28	-66 ± 12	-64 ± 13
		Gly	-62 ± 48	-54 ± 39	-57 ± 27
	Experiment	Pro(Xaa)		-77	
		Pro(Yaa)		-63	
		Gly		-70	
ψ	CHARMM36+TIP3P	Pro(Xaa)	145 ± 11	147 ± 10	149 ± 10
		Pro(Yaa)	141 ± 9	139 ± 9	140 ± 9
		Gly	171 ± 9	169 ± 10	172 ± 9
	CHARMM36+TIP4P	Pro(Xaa)	153 ± 10	152 ± 9	151 ± 11
		Pro(Yaa)	148 ± 10	150 ± 12	150 ± 11
		Gly	170 ± 9	172 ± 12	171 ± 14
	CHARMM36mGP+TIP4P	Pro(Xaa)	158 ± 12	159 ± 10	155 ± 14
		Pro(Yaa)	151 ± 15	155 ± 11	157 ± 11
		Gly	175 ± 16	173 ± 14	175 ± 12
	AMBER+TIP4P	Pro(Xaa)	155 ± 16	156 ± 17	168 ± 12
		Pro(Yaa)	155 ± 16	156 ± 17	169 ± 12
		Gly	170 ± 12	169 ± 16	168 ± 14
	AMOEBA	Pro(Xaa)	154 ± 46	158 ± 18	162 ± 15
		Pro(Yaa)	161 ± 34	154 ± 38	153 ± 32
		Gly	161 ± 41	161 ± 41	160 ± 24
	Experiment	Pro(Xaa)		164	
		Pro(Yaa)		154	
		Gly		175	
ω	CHARMM36+TIP3P	All	175 ± 4	177 ± 3	176 ± 4
	CHARMM36+TIP4P	All	175 ± 3	177 ± 4	176 ± 4
	CHARMM36mGP+TIP4P	All	176 ± 4	178 ± 3	176 ± 3
	AMBER+TIP4P	All	174 ± 4	175 ± 3	175 ± 4
	AMOEBA	All	176 ± 5	176 ± 4	176 ± 3
	Experiment	All		178	

Table S2: Dihedral angles (in degrees) for ϕ, ψ and ω from the CHARMM36+TIP3P, CHARMM36+TIP4P, CHARMM36mGP+TIP4P, AMBER+TIP4P and AMOEBA simulations, compared to the experimentally expected values.[6–10]

Angle	Force Field	Residue	$(PPG)_5$	$(PPG)_{12}$	$(PPG)_{25}$
χ	CHARMM36+TIP3P	Exo:Endo (Xaa)	70:30	74:26	69:31
		Exo:Endo (Yaa)	75:25	88:12	85:15
	CHARMM36+TIP4P	Exo:Endo (Xaa)	72:28	74:26	78:22
		Exo:Endo (Yaa)	75:25	83:17	80:20
	CHARMM36mGP+TIP4P	Exo:Endo (Xaa)	56:44	52:48	53:47
		Exo:Endo (Yaa)	84:16	79:21	80:20
	AMBER+TIP4P	Exo:Endo (Xaa)	51:49	56:44	51:49
		Exo:Endo (Yaa)	78:22	80:20	77:23
	AMOEBA	Exo:Endo (Xaa)	54:46	46:54	49:51
		Exo:Endo (Yaa)	72:28	75:25	75:25
	Experiment	Exo:Endo (Xaa)		50:50	
		Exo:Endo (Yaa)		78:22	
	CHARMM36+TIP3P	Endo	34 ± 7	29 ± 8	30 ± 7
		Exo	-29 ± 7	-27 ± 8	-27 ± 7
	CHARMM36+TIP4P	Endo	29 ± 5	29 ± 5	29 ± 5
		Exo	-27 ± 4	-27 ± 4	-27 ± 4
	CHARMM36mGP+TIP4P	Endo	34 ± 7	29 ± 8	30 ± 7
		Exo	-29 ± 7	-27 ± 8	-27 ± 7
	AMBER+TIP4P	Endo	33 ± 10	34 ± 9	33 ± 9
		Exo	-31 ± 12	-32 ± 9	-31 ± 7
	AMOEBA	Endo	34 ± 16	34 ± 11	34 ± 11
		Exo	-33 ± 22	-33 ± 12	-32 ± 9
	Experiment	Endo		31	
		Exo		-30	

Table S3: χ Dihedral angles (in degrees) from the CHARMM36+TIP3P, CHARMM36+TIP4P, CHARMM36mGP+TIP4P, AMBER+TIP4P and AMOEBA simulations, compared to the experimentally expected values.[6–10] The ratio of Exo:Endo for each position and the average χ for each type of ring pucker is shown.

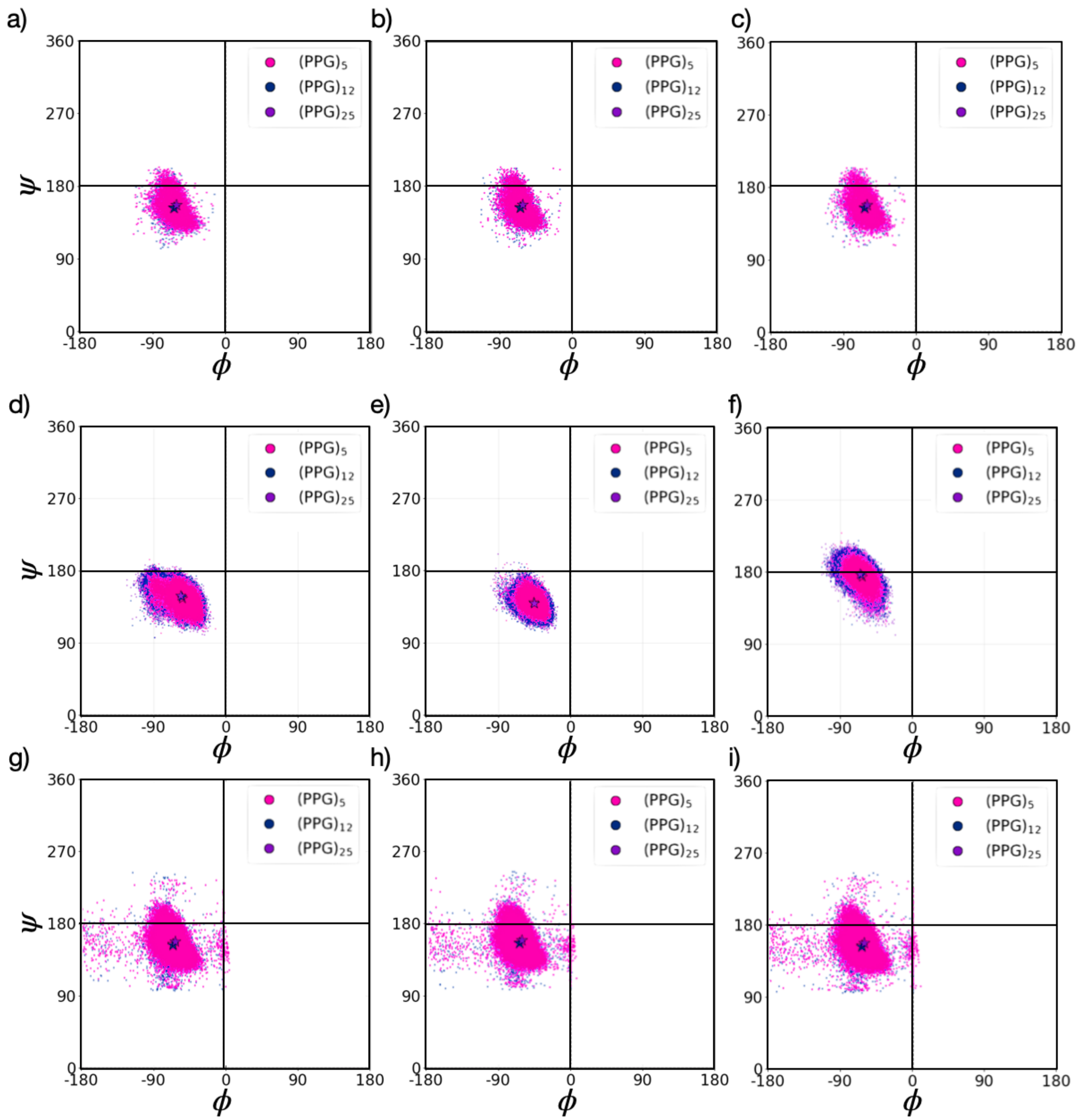

Figure S10: Ramachandran plots of the $(\text{PPG})_n$ proteins. The stars show the mean of the angles, which are expected to be $\langle(\phi, \psi)\rangle \approx (-77°, 164°)$ for Pro(Xaa), $\langle(\phi, \psi)\rangle \approx (-63°, 154°)$ for Pro(Yaa) and $\langle(\phi, \psi)\rangle \approx (-70°, 175°)$ for Gly.[6–10] a) Pro(Xaa), CHARMM36+TIP3P. b) Pro(Yaa), CHARMM36+TIP3P. c) Gly, CHARMM36+TIP3P. d) Pro(Xaa), CHARMM36+TIP4P. e) Pro(Yaa), CHARMM36+TIP4P. f) Gly, CHARMM36+TIP4P. g) Pro(Xaa), CHARMM36mGP+TIP4P. h) Pro(Yaa), CHARMM36mGP+TIP4P. i) Gly, CHARMM36mGP+TIP4P. Figure continued on the next page with plots for AMBER+TIP4P and AMOEBA.

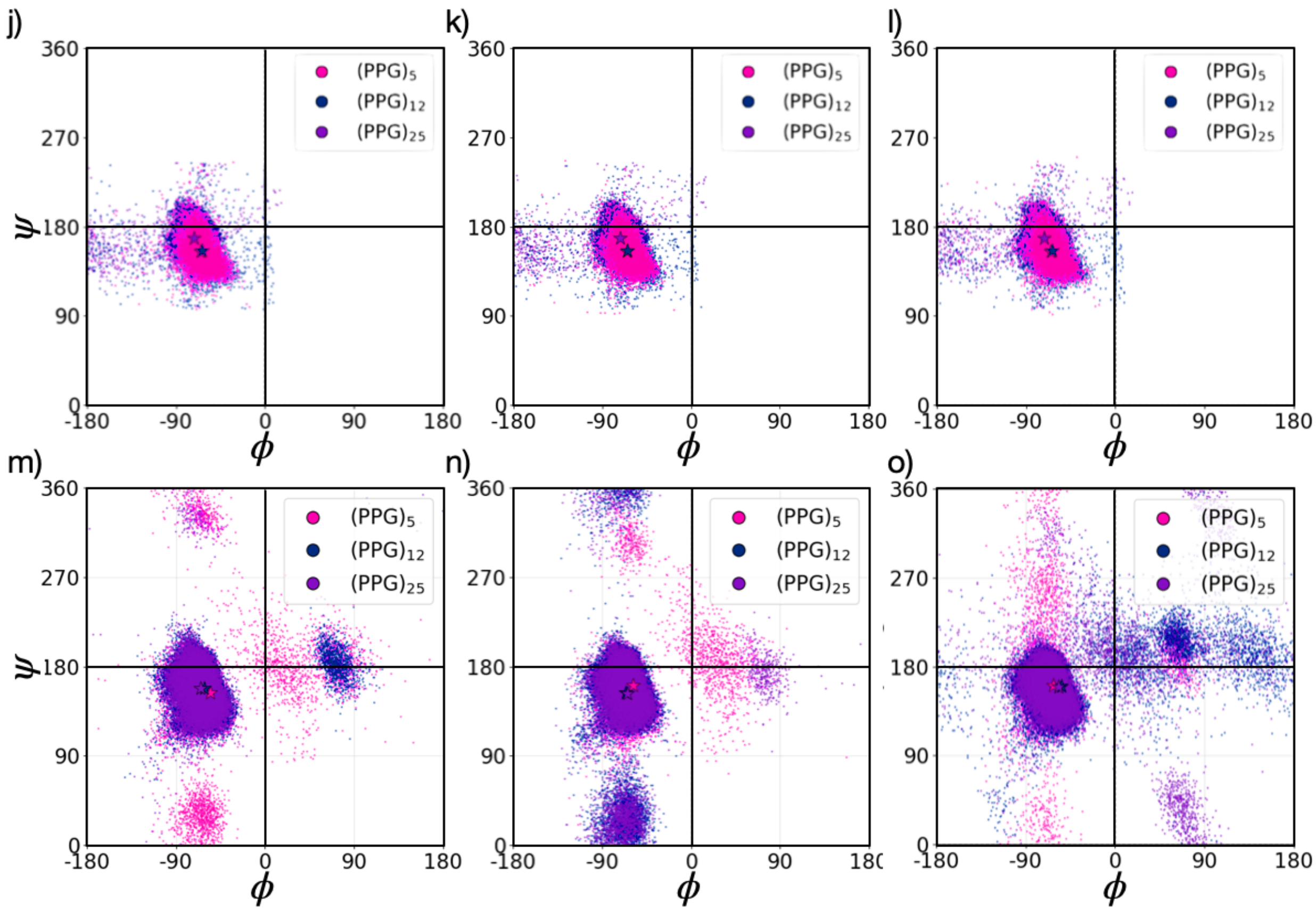

Figure S10: Ramachandran plots of the $(PPG)_n$ proteins (continued from last page). The stars show the mean of the angles, which are expected to be $\langle(\phi, \psi)\rangle \approx (-77°, 164°)$ for Pro(Xaa), $\langle(\phi, \psi)\rangle \approx (-63°, 154°)$ for Pro(Yaa) and $\langle(\phi, \psi)\rangle \approx (-70°, 175°)$ for Gly.[6–10] j) Pro(Xaa), AMBER+TIP4P. k) Pro(Yaa), AMBER+TIP4P. l) Gly, AMBER+TIP4P. m) Pro(Xaa), AMOEBA. n) Pro(Yaa), AMOEBA. o) Gly, AMOEBA.

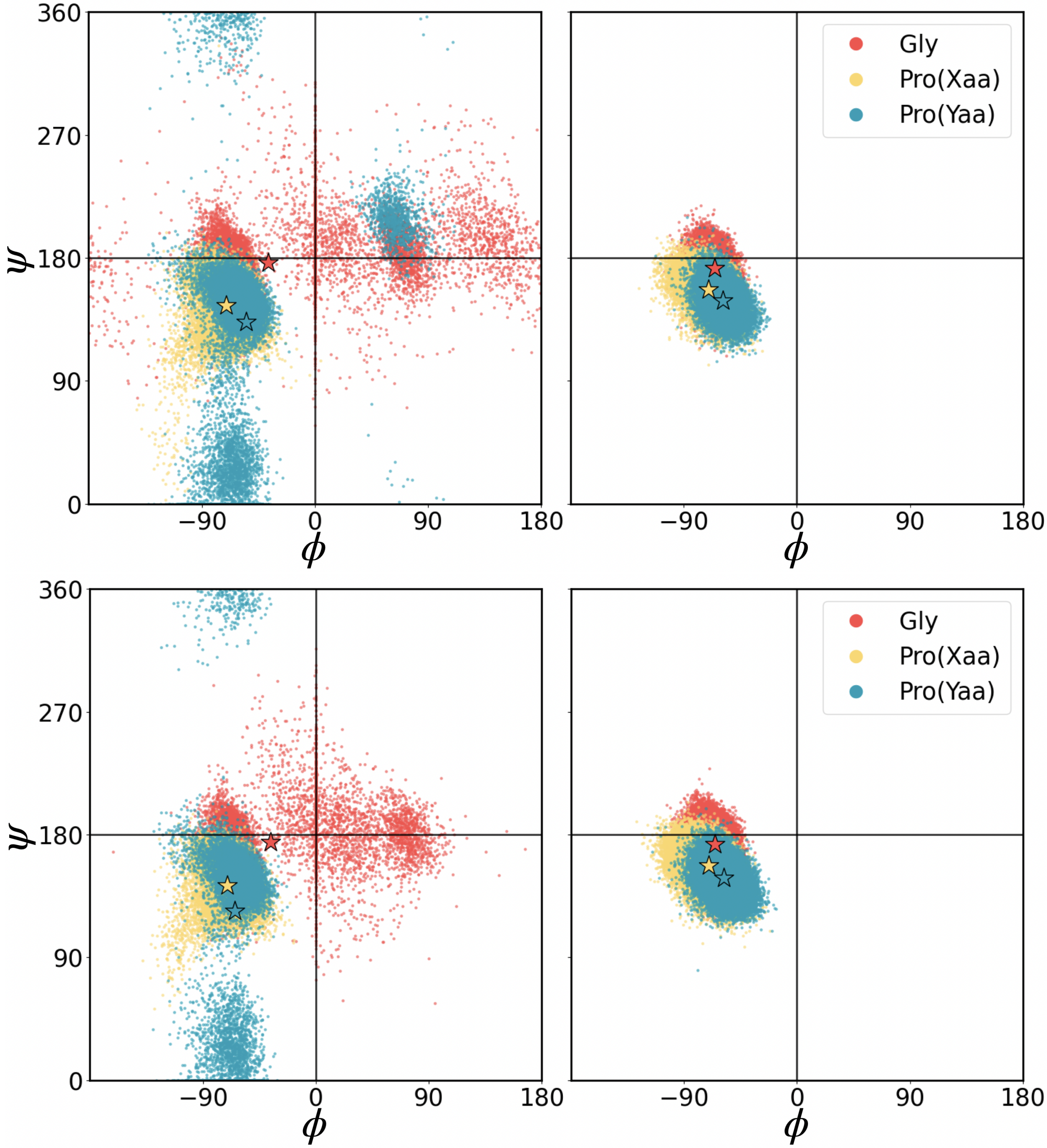

Figure S11: Top row: Ramachandran plot for $(PPG)_{12}$ deformed peptides -4 (left) and non-deformed tripeptides #5-12 (right). Bottom row: Ramachandran plot for $(PPG)_{12}$ deformed peptides -2 (left) and non-deformed tripeptides #3-25 (right). The standard deviation in angle is less than 15 for non-deformed tripeptides. All tripeptides are deformed in $(PPG)_5$.

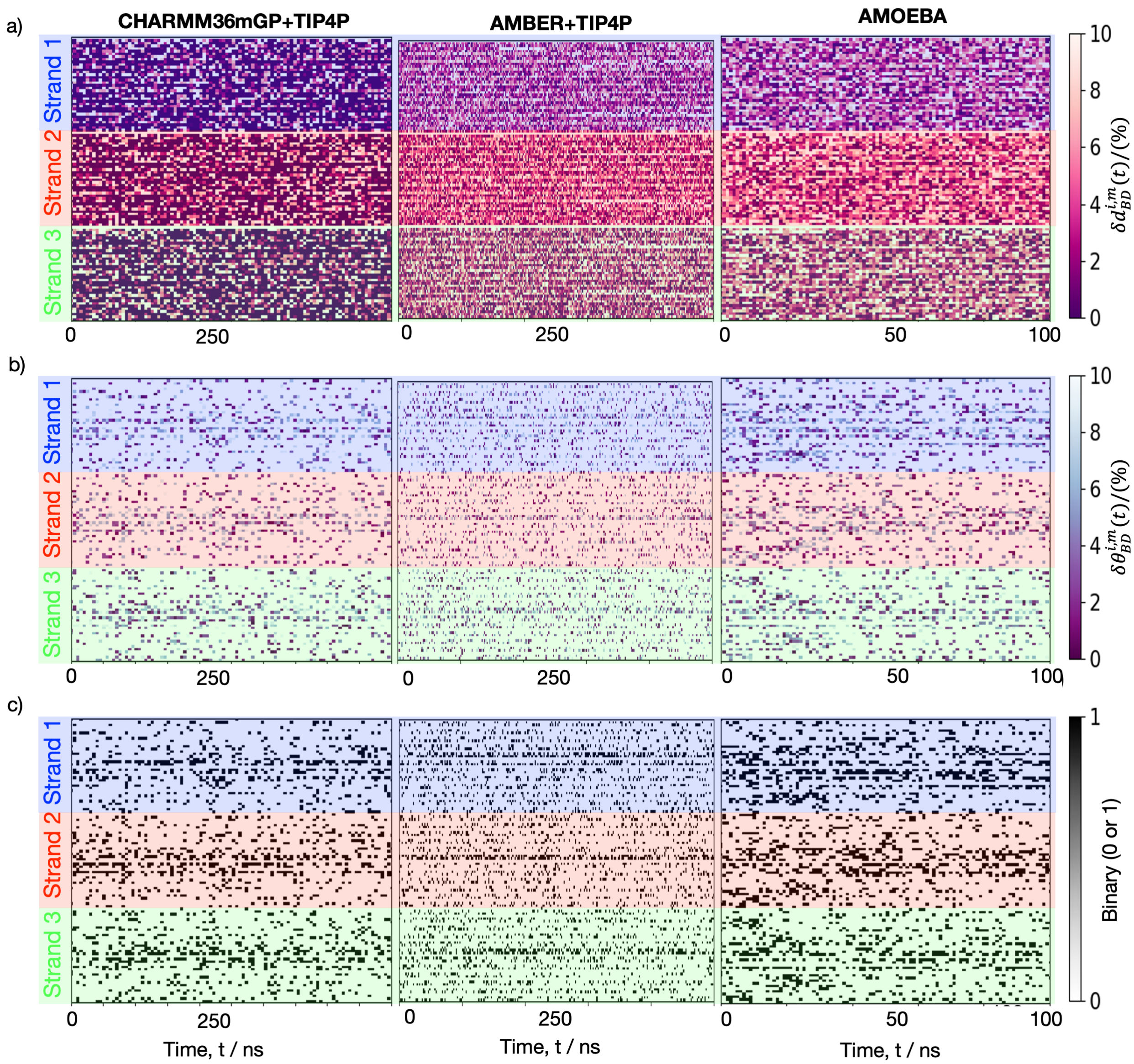

Figure S12: Geometric conditions to determine n→ π^* interactions in $(PPG)_{12}$ with CHARMM36mGP+TIP4P (left column), AMBER+TIP4P (middle column) and AMOEBA (right column). The y-axis indicate residue number, with the three strand highlighted in blue, red and green strands, for clarity. a) Time frames where $\delta d_{BD}^{i,m} \leq 10\%$. b) Time frames where $\delta\theta_{BD}^{i,m} \leq 10\%$. c) Time frames where both $\delta\theta_{BD}^{i,m} \leq 10\%$ and $\delta d_{BD}^{i,m} \leq 10\%$.

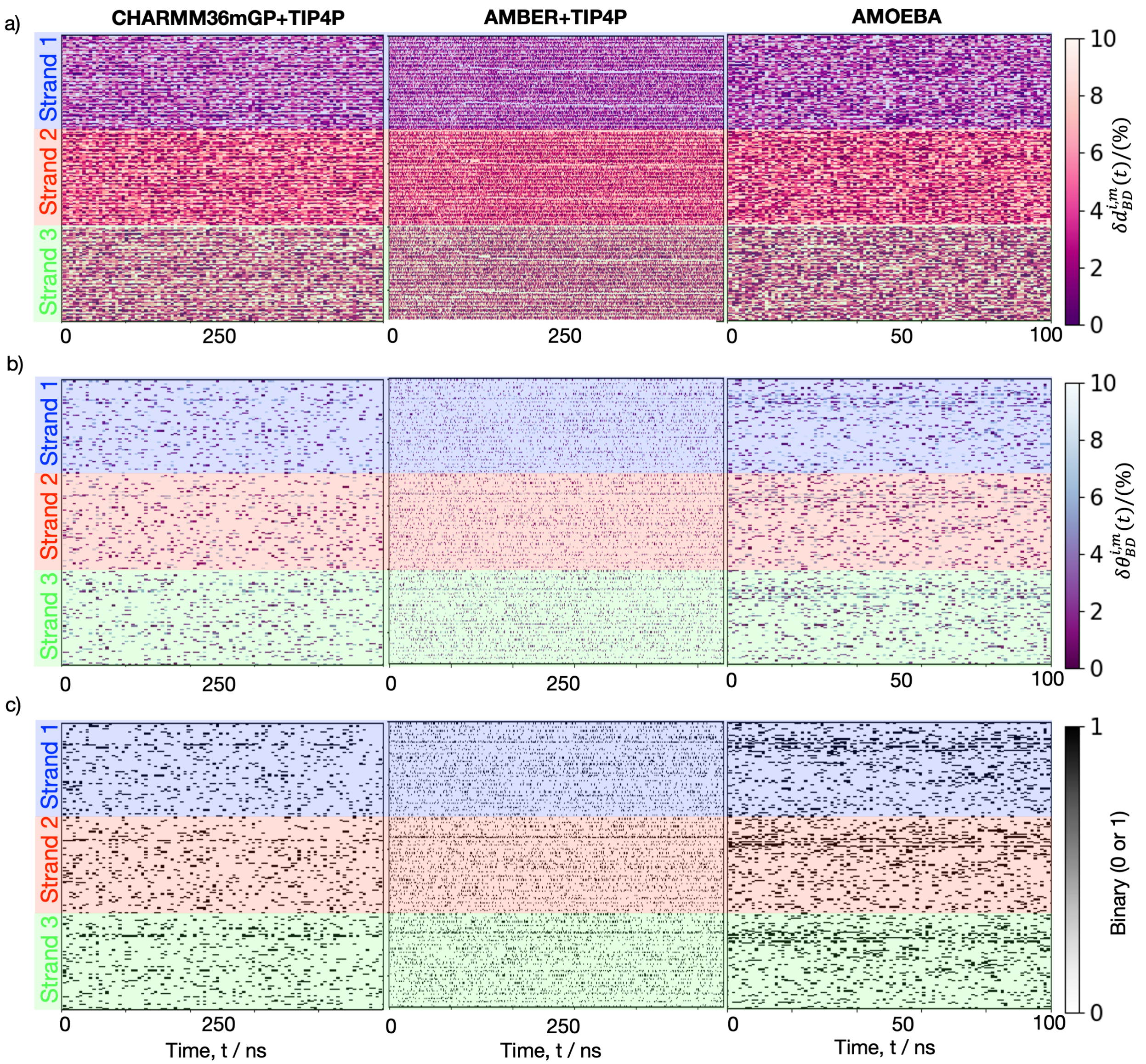

Figure S13: Geometric conditions to determine n→ π^* interactions in $(PPG)_{25}$ with CHARMM36mGP+TIP4P (left column), AMBER+TIP4P (middle column) and AMOEBA (right column). The y-axis indicate residue number, with the three strand highlighted in blue, red and green strands, for clarity. a) Time frames where $\delta d_{BD}^{i,m} \leq 10\%$. b) Time frames where $\delta\theta_{BD}^{i,m} \leq 10\%$. c) Time frames where both $\delta\theta_{BD}^{i,m} \leq 10\%$ and $\delta d_{BD}^{i,m} \leq 10\%$.

Deformation of the triple helix during the MD simulations

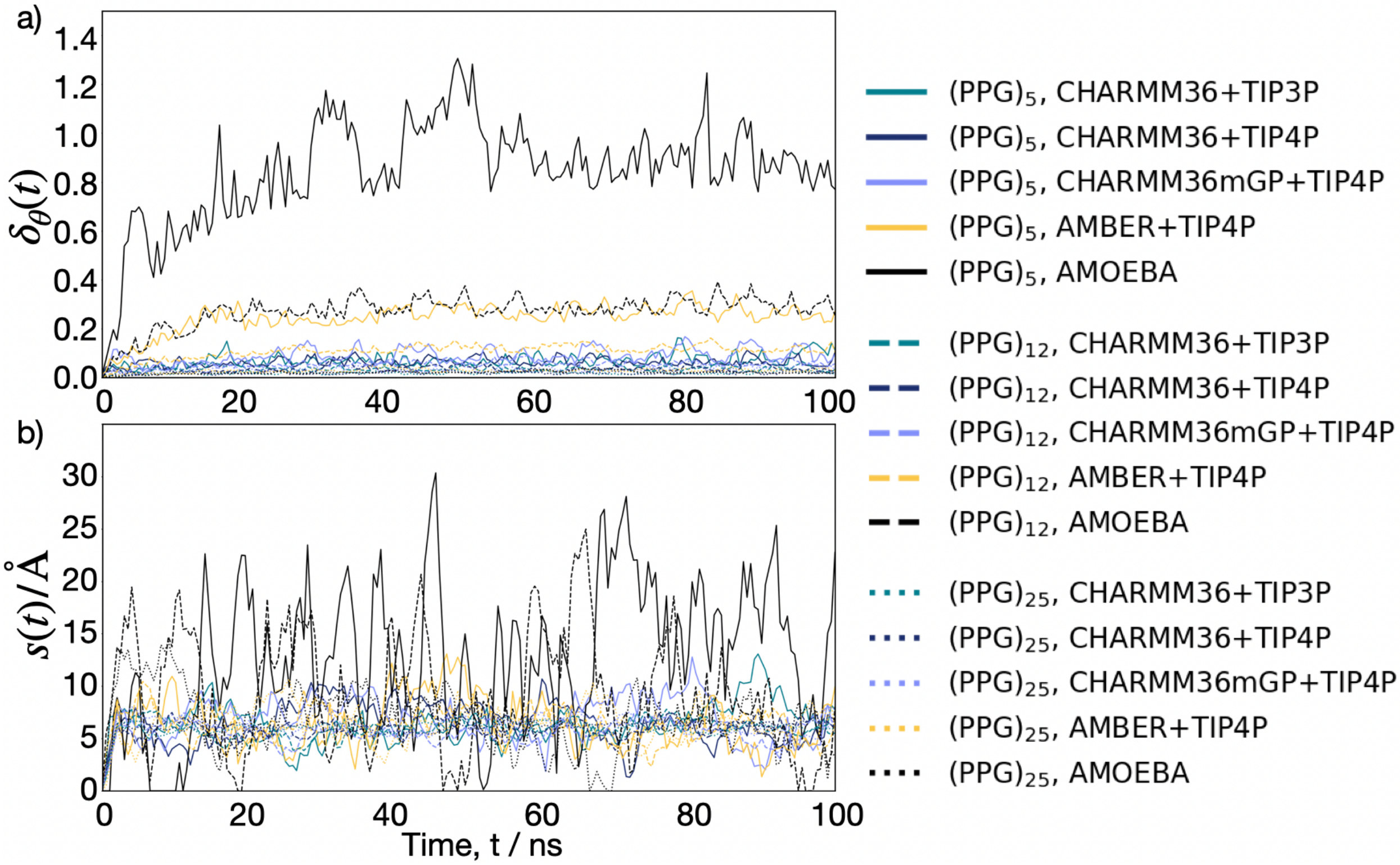

Figure S14: a) Average axis deviation $\delta_\theta(t)$ over time for $(\mathrm{PPG})_n$ in CHARMM36+TIP3P, CHARMM36+TIP4P, CHARMM36mGP+TIP4P, AMBER+TIP4P and AMOEBA. b) Axial shift $s(t)$ over time for $(\mathrm{PPG})_n$ in CHARMM36+TIP3P, CHARMM36+TIP4P, CHARMM36mGP+TIP4P, AMBER+TIP4P and AMOEBA.

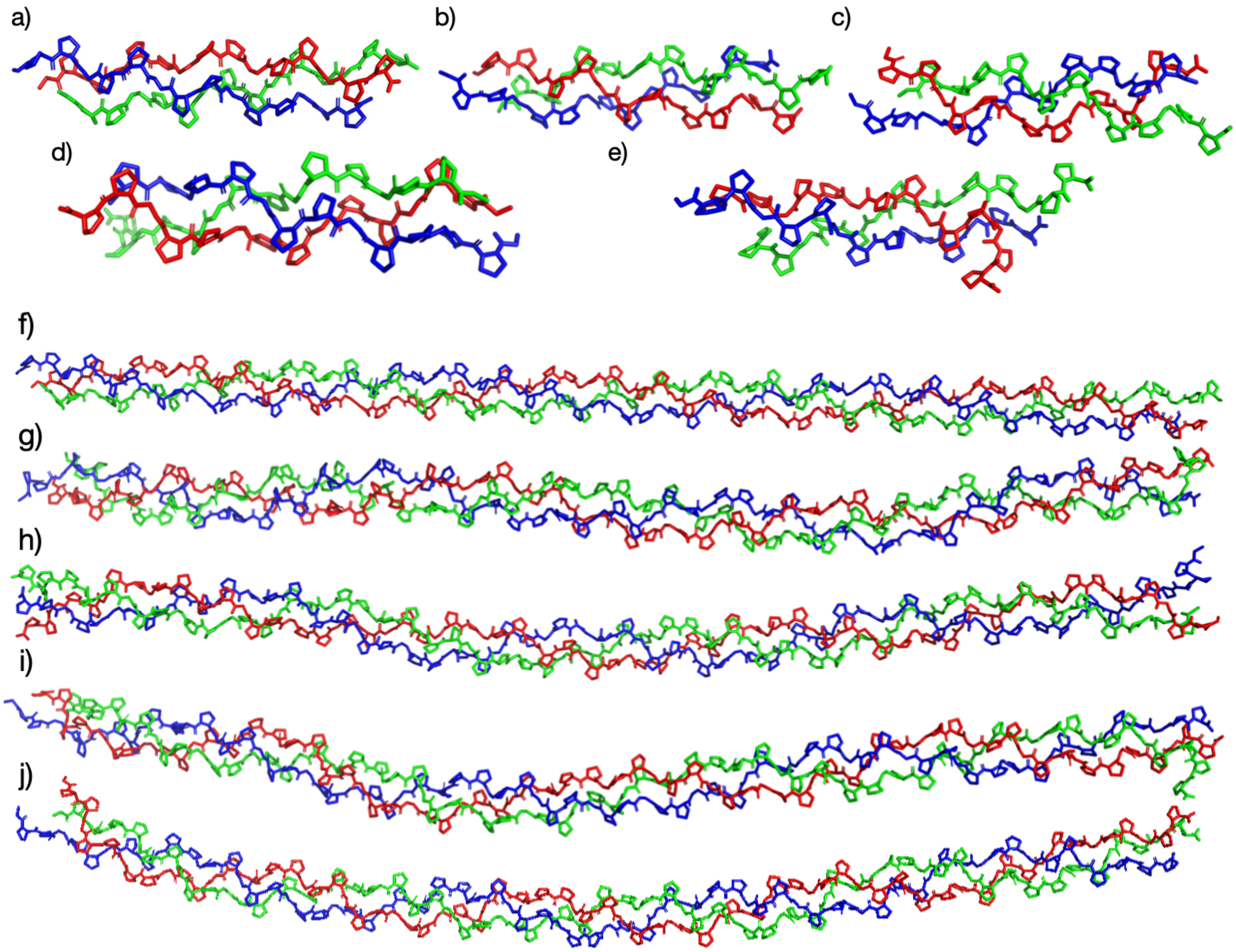

Figure S15: Snapshots from the end of the simulation. Water molecules and hydrogen atoms are omitted for clarity. a) $(PPG)_5$ with CHARMM36+TIP3P. b) $(PPG)_5$ with CHARMM36+TIP4P. c) $(PPG)_5$ with CHARMM36mGP+TIP4P. d) $(PPG)_5$ with AMBER+TIP4P. e) $(PPG)_5$ with AMOEBA. f) $(PPG)_{25}$ with CHARMM36+TIP3P. g) $(PPG)_{25}$ with CHARMM36+TIP4P. h) $(PPG)_{25}$ with CHARMM36mGP+TIP4P. i) $(PPG)_{25}$ with AMBER+TIP4P. j) $(PPG)_{25}$ with AMOEBA.

Movie S1 shows time evolution of $\langle \kappa^{i,m}(t) \rangle_{i,m} - \langle \zeta^i(t) \rangle_i$ correlations in $(PPG)_{12}$ without D-glucose.

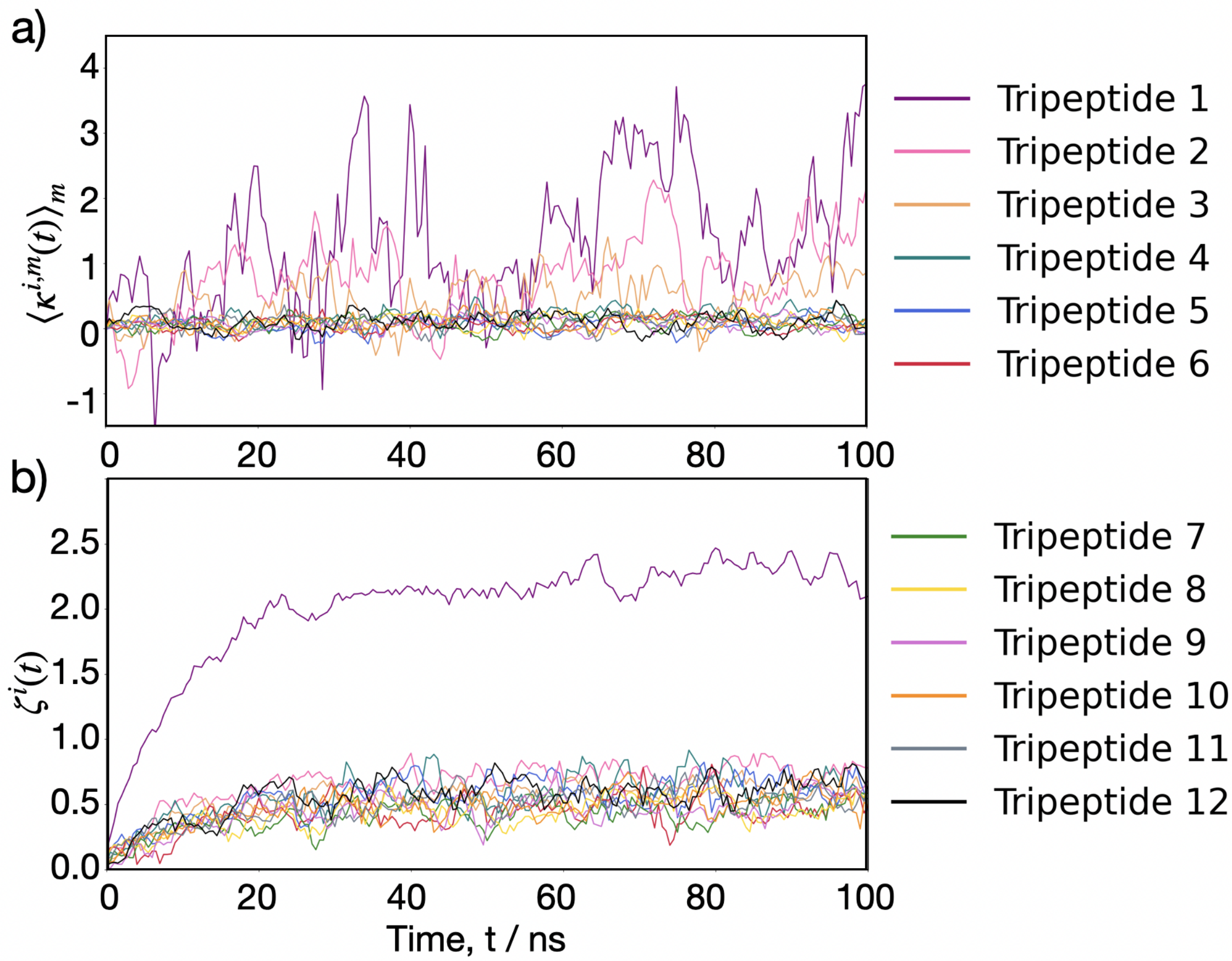

Figure S16: a) $\langle \kappa^{i,m}(t) \rangle_m$ over time for each tripeptide in $(PPG)_{12}$ without D-glucose in AMOEBA. a) $\zeta^i(t)$ over time for each tripeptide in $(PPG)_{12}$ without D-glucose in AMOEBA.

Hydrogen bonds and hydration analysis

**HB**	$\mathbf{(PPG)_5}$	$\mathbf{(PPG)_{12}}$	$\mathbf{(PPG)_{25}}$
Gly(NH)-Xaa(CO) (0)	5	12	25
$\langle$ Gly(NH)-Xaa(CO) $\rangle_t$	1 ± 1	9 ± 2	20 ± 4
Xaa(C_α)-CO (0)	1	1	2
$\langle$ Xaa(C_α)-CO $\rangle_t$	0 ± 1	3 ± 2	5 ± 3

Table S4: Table of inter-strand HBs for $(PPG)_n$ CMPs without any D-glucose from AMOEBA. The rows with "(0)" denote the number of HBs at the beginning of the simulation. The mean over time is shown with the standard deviation.

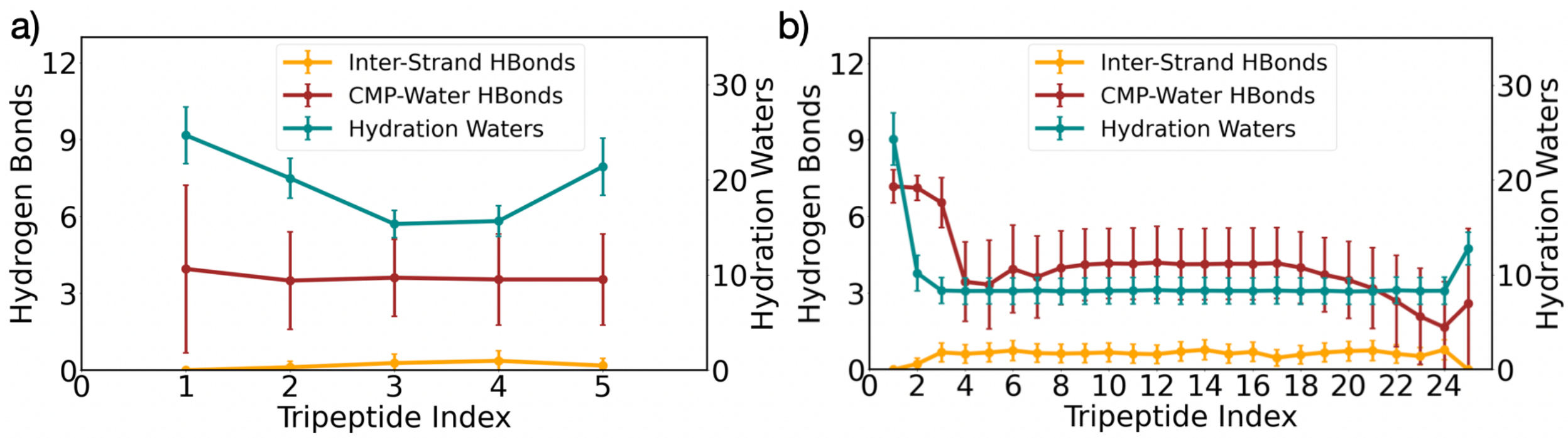

Figure S17: Average inter-strand HBs, CMP-water HBs and hydration waters for each tripeptide for $(PPG)_n$ without any D-glucose in AMOEBA. a) $(PPG)_5$. b) $(PPG)_{25}$. The profile for $(PPG)_{12}$ is in the main text.

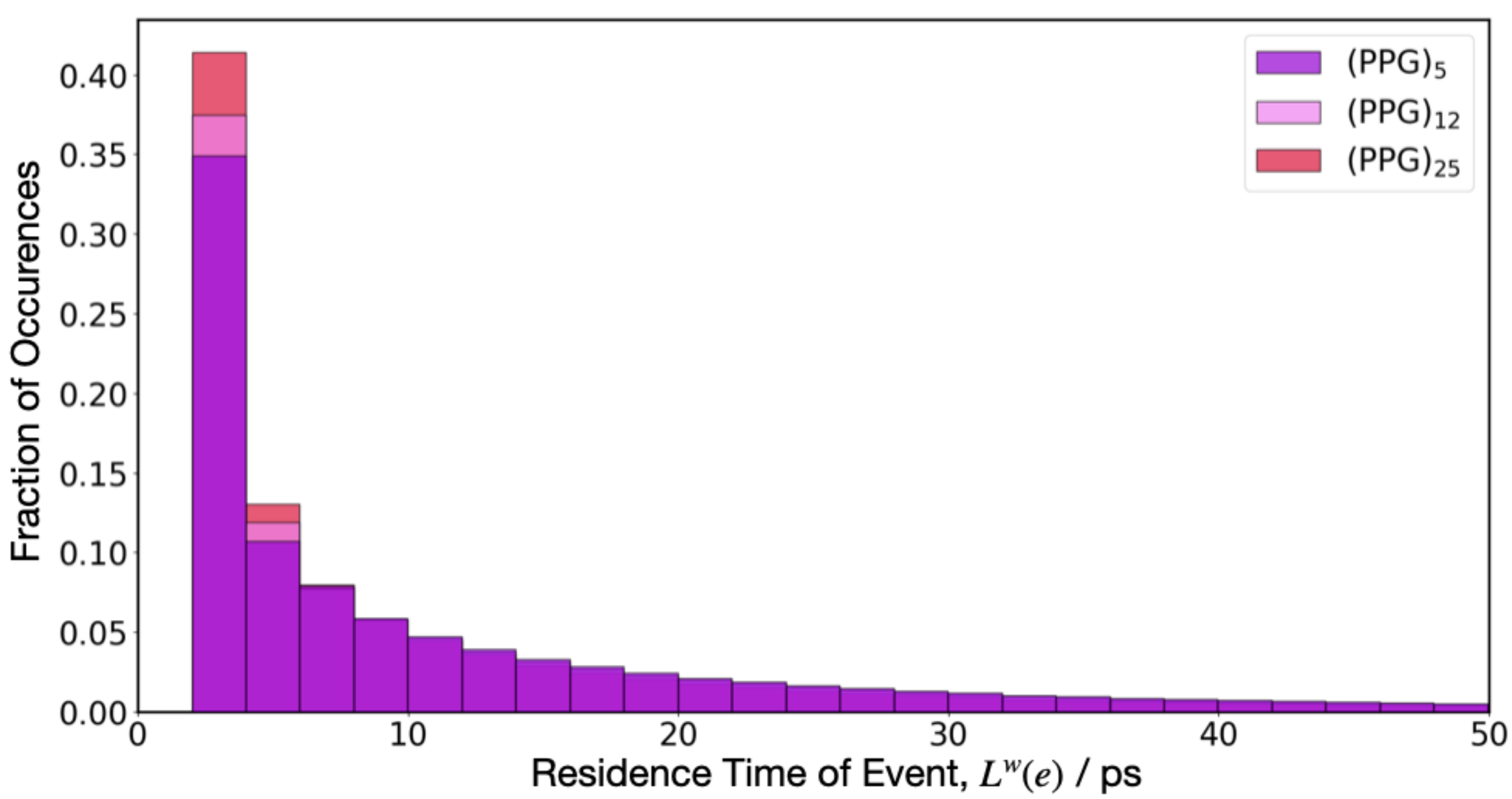

Figure S18: Distribution of residence times $L^w(e)$ for hydration shell waters of $(PPG)_n$ CMPs.

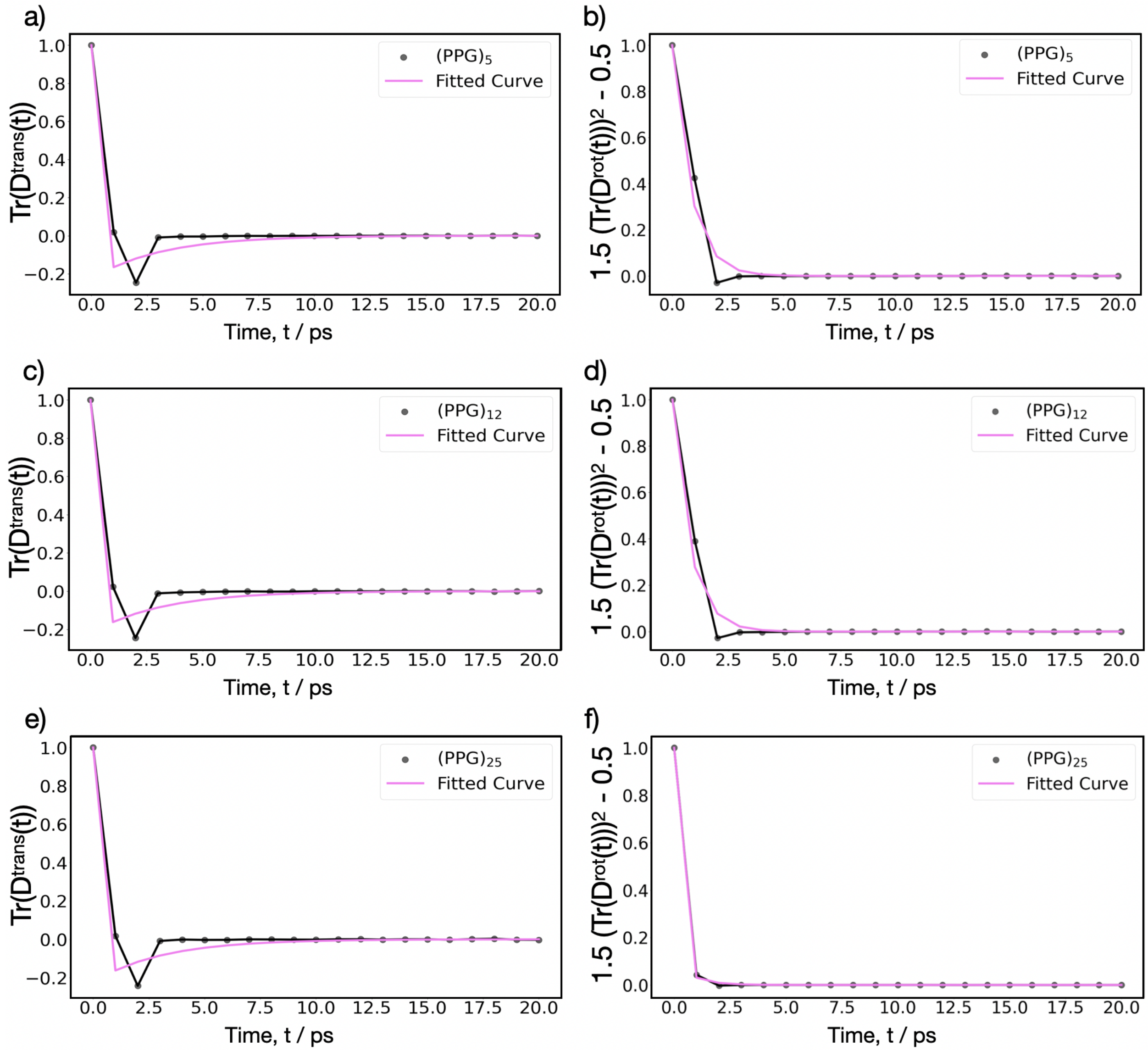

Figure S19: Decay of $\mathrm{Tr}(D^{\mathrm{trans}}(t))$ and $\frac{1}{2}(3(\mathrm{Tr}(D^{\mathrm{rot}}(t)))^2-1)$ for hydration shell waters of $(\mathrm{PPG})_n$. The black circles and line show the actual data, and the violet line shows the best-fit curve based on a biexponential fit for translation and monoexponential fit for rotation. a) Translation for $(\mathrm{PPG})_5$, with $A^{\mathrm{trans}} = 1.23$, $B^{\mathrm{trans}} = -0.23$, $\tau_1^{\mathrm{trans}} = 0.28$ ps, $\tau_2^{\mathrm{trans}} = 3.15$ ps and $R^2 = 0.899$. b) Rotation for $(\mathrm{PPG})_5$, with $A^{\mathrm{rot}} = 1.00$, $\tau^{\mathrm{rot}} = 0.65$ ps and $R^2 = 0.871$. c) Translation for $(\mathrm{PPG})_{12}$, with $A^{\mathrm{trans}} = 1.22$, $B^{\mathrm{trans}} = -0.22$, $\tau_1^{\mathrm{trans}} = 0.3$ ps, $\tau_2^{\mathrm{trans}} = 3.15$ ps and $R^2 = 0.899$. d) Rotation for $(\mathrm{PPG})_{12}$, with $A^{\mathrm{rot}} = 1.00$, $\tau^{\mathrm{rot}} = 0.78$ ps and $R^2 = 0.953$. e) Translation for $(\mathrm{PPG})_{25}$, with $A^{\mathrm{trans}} = 1.23$, $B^{\mathrm{trans}} = -0.23$, $\tau_1^{\mathrm{trans}} = 0.35$ ps, $\tau_2^{\mathrm{trans}} = 3.35$ ps and $R^2 = 0.821$. f) Rotation for $(\mathrm{PPG})_{25}$, with $A^{\mathrm{rot}} = 1.00$, $\tau^{\mathrm{rot}} = 0.80$ ps and $R^2 = 0.993$.

Effect of stress factors on $(PPG)_n$ CMPs

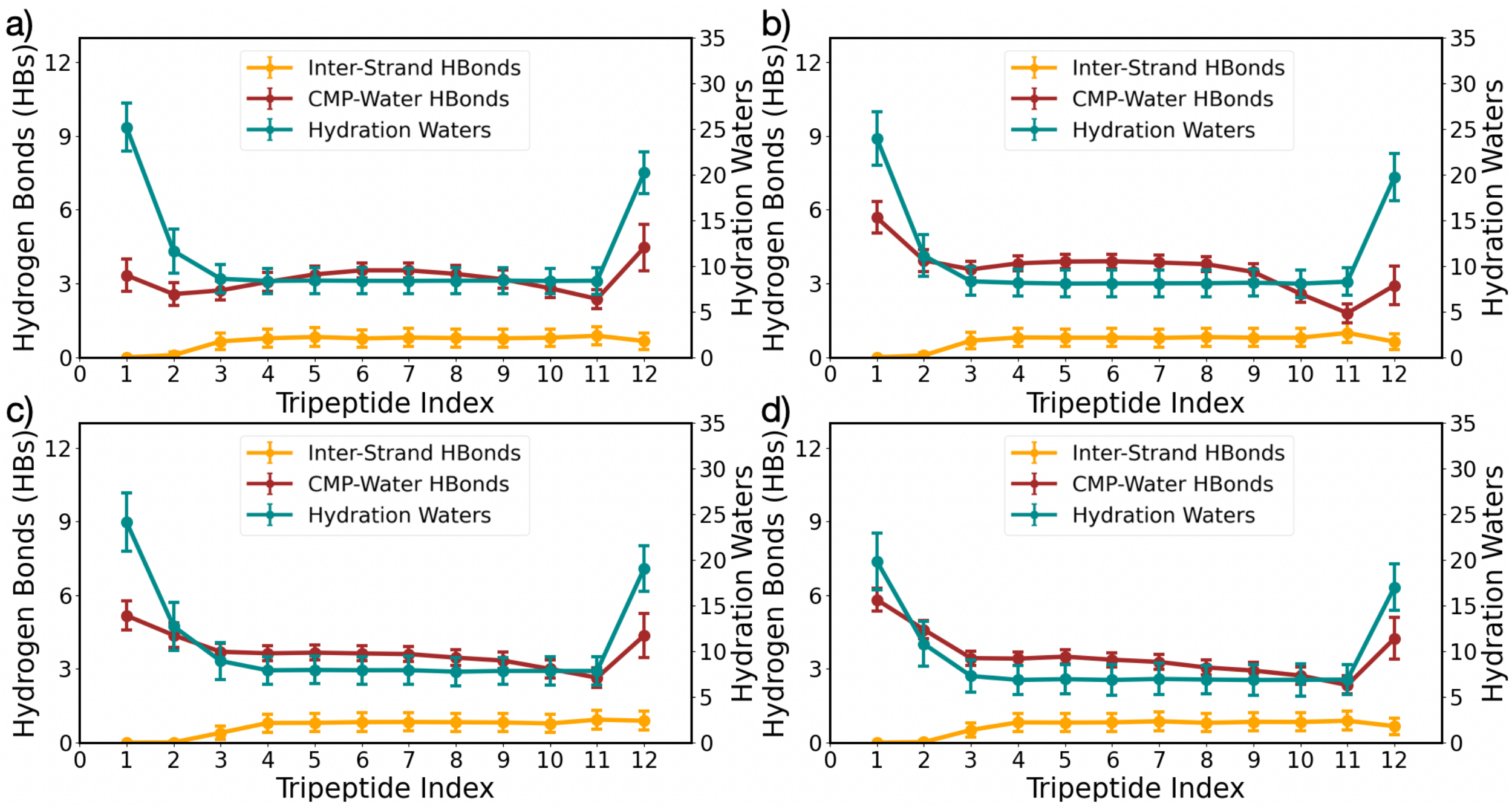

Figure S20: Average inter-strand HBs, collagen-water HBs and number of hydration waters for each tripeptide of $(PPG)_{12}$. a) 0 mM D-glucose: this is also in the main text. b) 500 mM D-glucose. c) 1000 mM D-glucose. d) 2000 mM D-glucose.

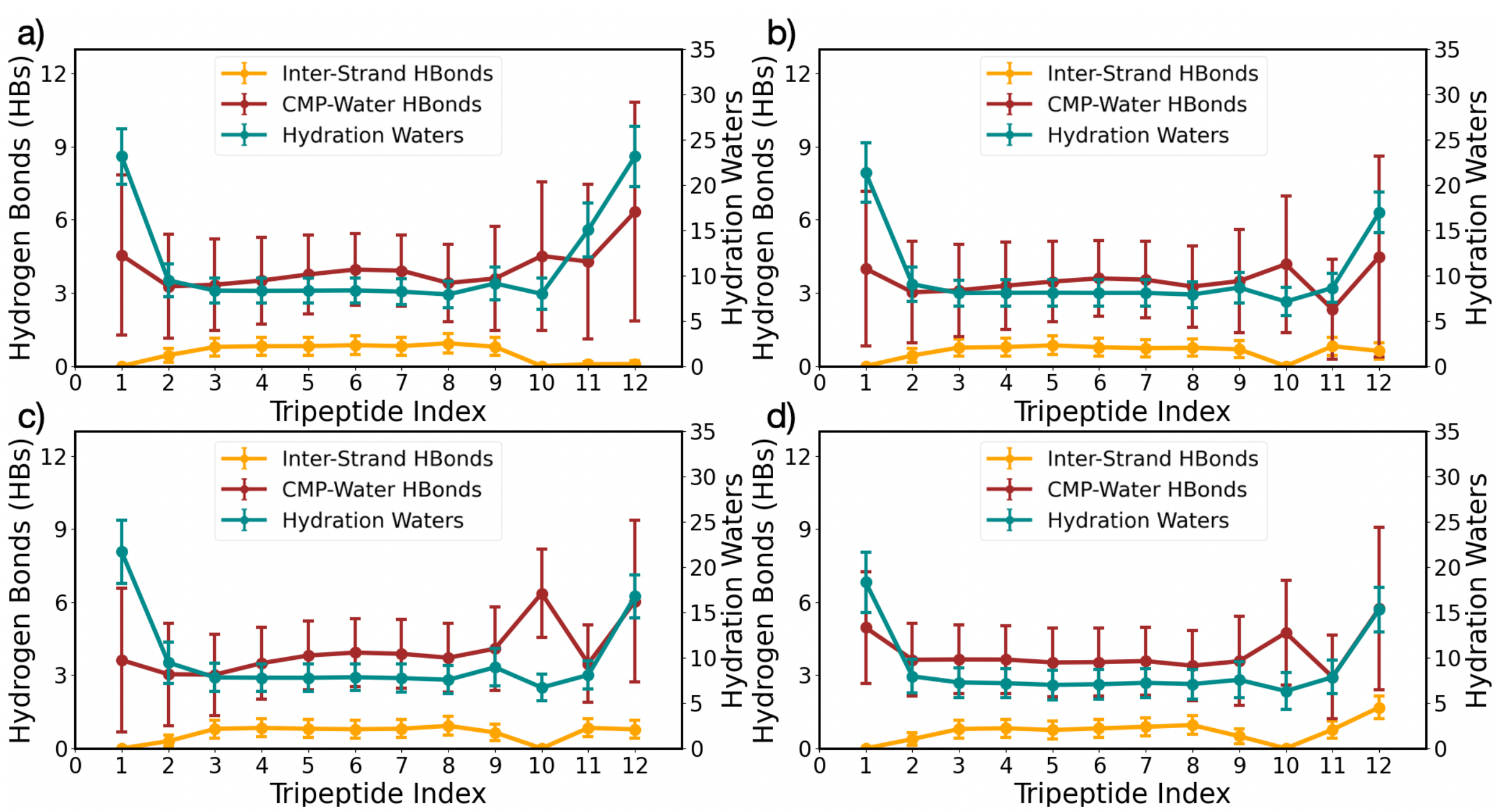

Figure S21: Average inter-strand HBs, CMP-water HBs and hydration waters for each tripeptide of Gly$\xrightarrow{\text{end}}$Ala. a) 0 mM D-glucose. b) 500 mM D-glucose. c) 1000 mM D-glucose. d) 2000 mM D-glucose.

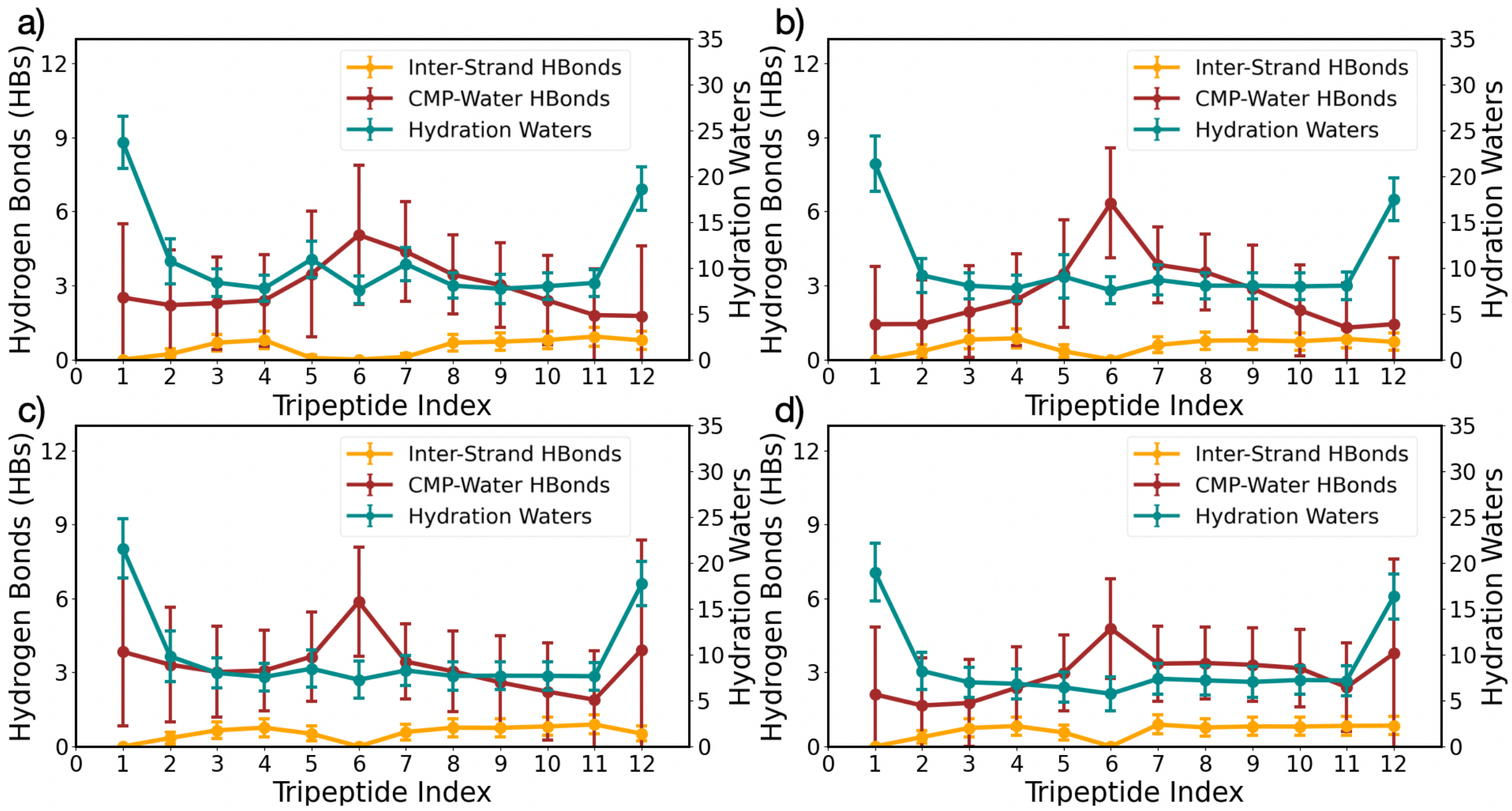

Figure S22: Average inter-strand HBs, CMP-water HBs and hydration waters for each tripeptide of Gly$\xrightarrow{\text{mid}}$Ala. a) 0 mM D-glucose. b) 500 mM D-glucose. c) 1000 mM D-glucose. d) 2000 mM D-glucose.

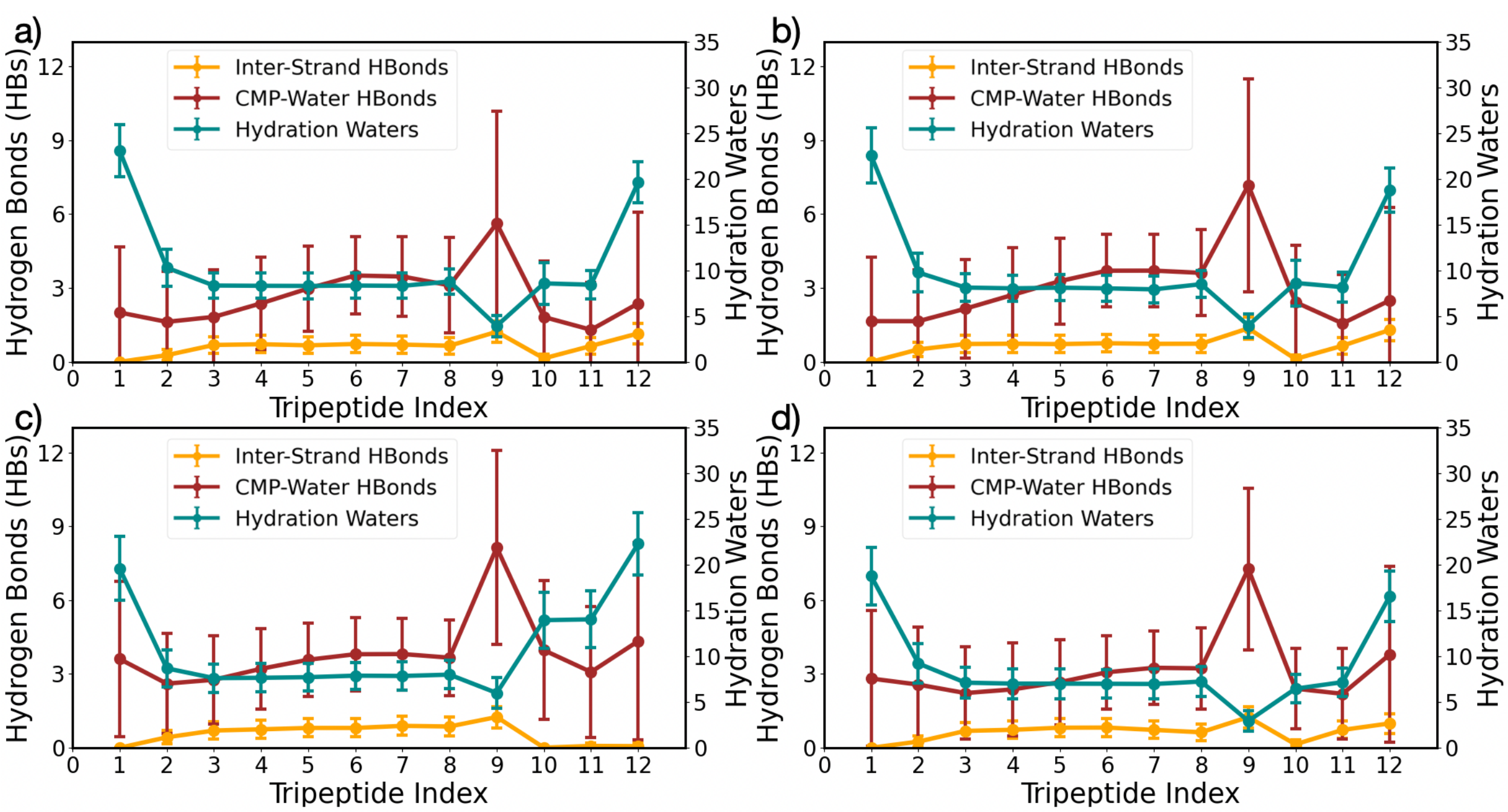

Figure S23: Average inter-strand HBs, CMP-water HBs and hydration waters for each tripeptide of Pro$\xrightarrow{\text{end}}$Lys. a) 0 mM D-glucose. b) 500 mM D-glucose. c) 1000 mM D-glucose. d) 2000 mM D-glucose.

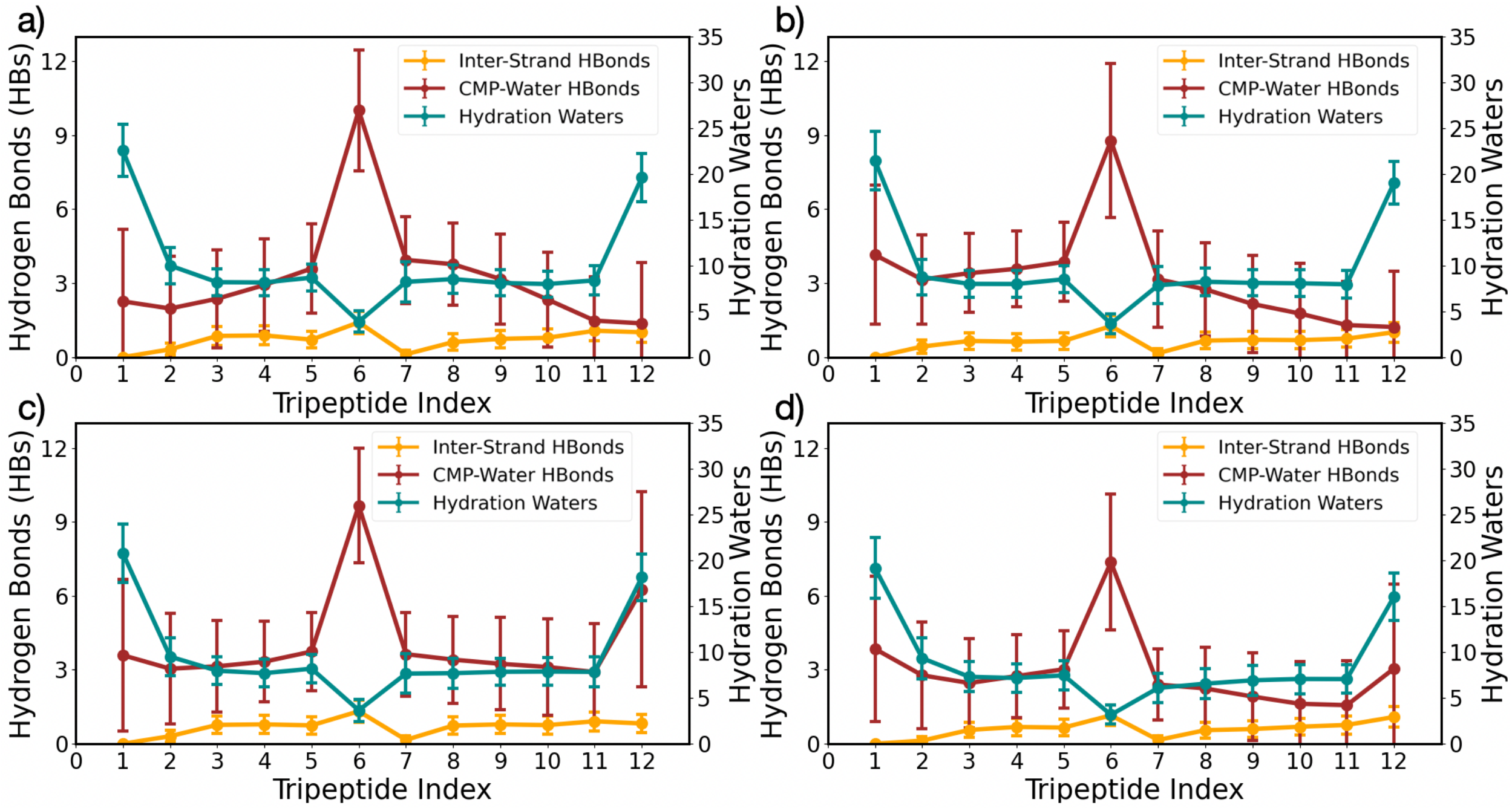

Figure S24: Average inter-strand HBs, CMP-water HBs and hydration waters for each tripeptide of Pro$\xrightarrow{\text{mid}}$Lys. a) 0 mM D-glucose. b) 500 mM D-glucose. c) 1000 mM D-glucose. d) 2000 mM D-glucose.

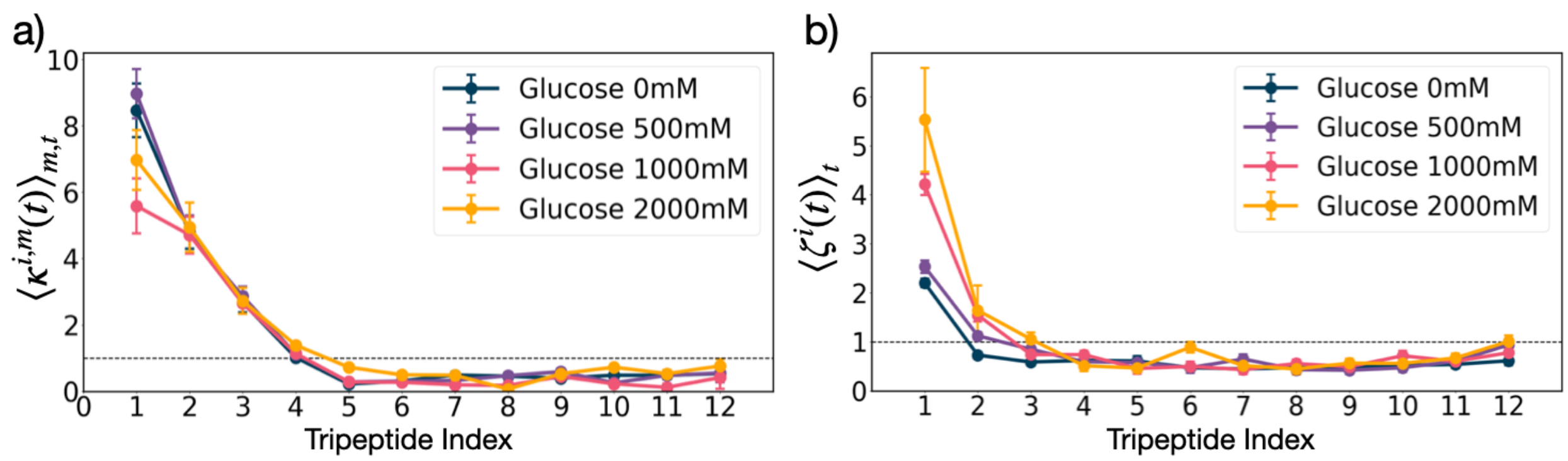

Figure S25: Deformation of the $(PPG)_{12}$ CMPs over tripeptide index. a) $\langle \kappa^{i,m}(t) \rangle_{m,t}$. b) $\langle \zeta^{i}(t) \rangle_{t}$. Every three consecutive residues have been averaged into a PPG tripeptide.

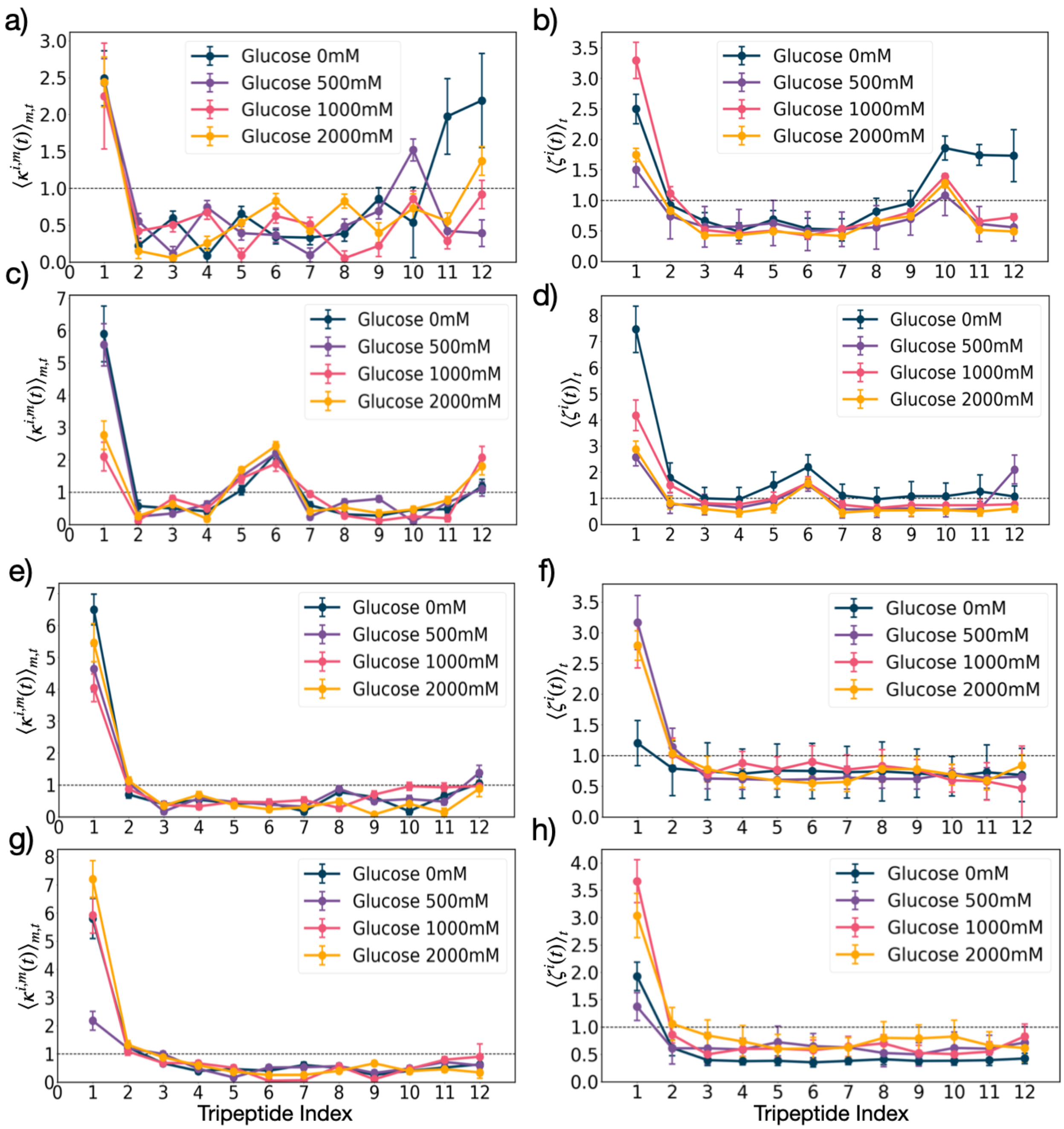

Figure S26: Deformation of the mutated CMPs over tripeptide index. a) Gly$\xrightarrow{\text{end}}$Ala $\langle \kappa^{i,m}(t)\rangle_{m,t}$. b) Gly$\xrightarrow{\text{end}}$Ala $\langle \zeta^{i}(t)\rangle_{t}$. c) Gly$\xrightarrow{\text{mid}}$Ala $\langle \kappa^{i,m}(t)\rangle_{m,t}$. d) Gly$\xrightarrow{\text{mid}}$Ala $\langle \zeta^{i}(t)\rangle_{t}$. e) Pro$\xrightarrow{\text{end}}$Lys $\langle \kappa^{i,m}(t)\rangle_{m,t}$. f) Pro$\xrightarrow{\text{end}}$Lys $\langle \zeta^{i}(t)\rangle_{t}$. g) Pro$\xrightarrow{\text{mid}}$Lys $\langle \kappa^{i,m}(t)\rangle_{m,t}$. h) Pro$\xrightarrow{\text{mid}}$Lys $\langle \zeta^{i}(t)\rangle_{t}$.

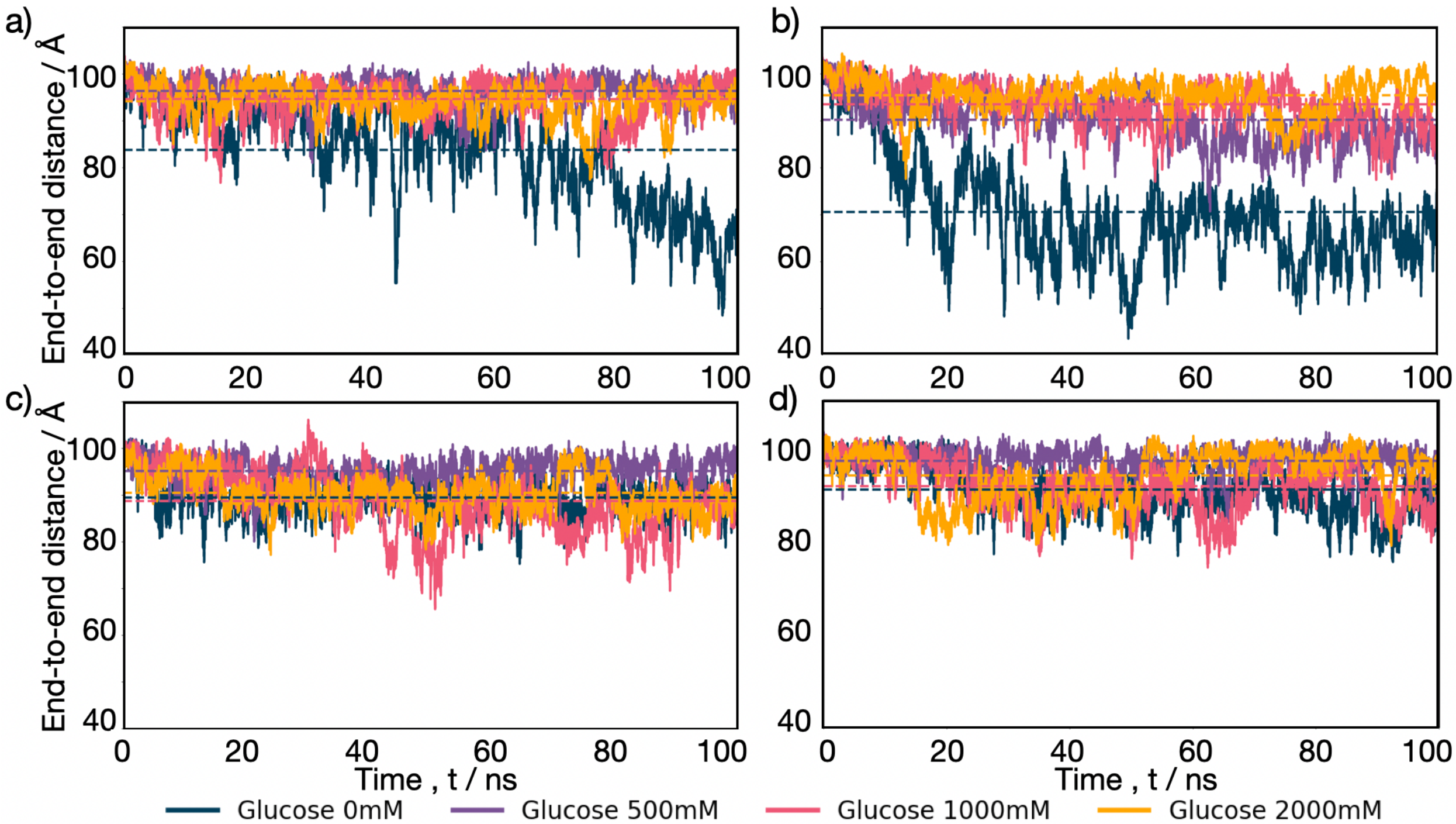

Figure S27: End-to-end distances for a) Gly$\xrightarrow{\text{end}}$Ala, b) Gly$\xrightarrow{\text{mid}}$Ala, c) Pro$\xrightarrow{\text{end}}$Lys, d) Pro$\xrightarrow{\text{mid}}$Lys. The horizontal dashed lines show the average.

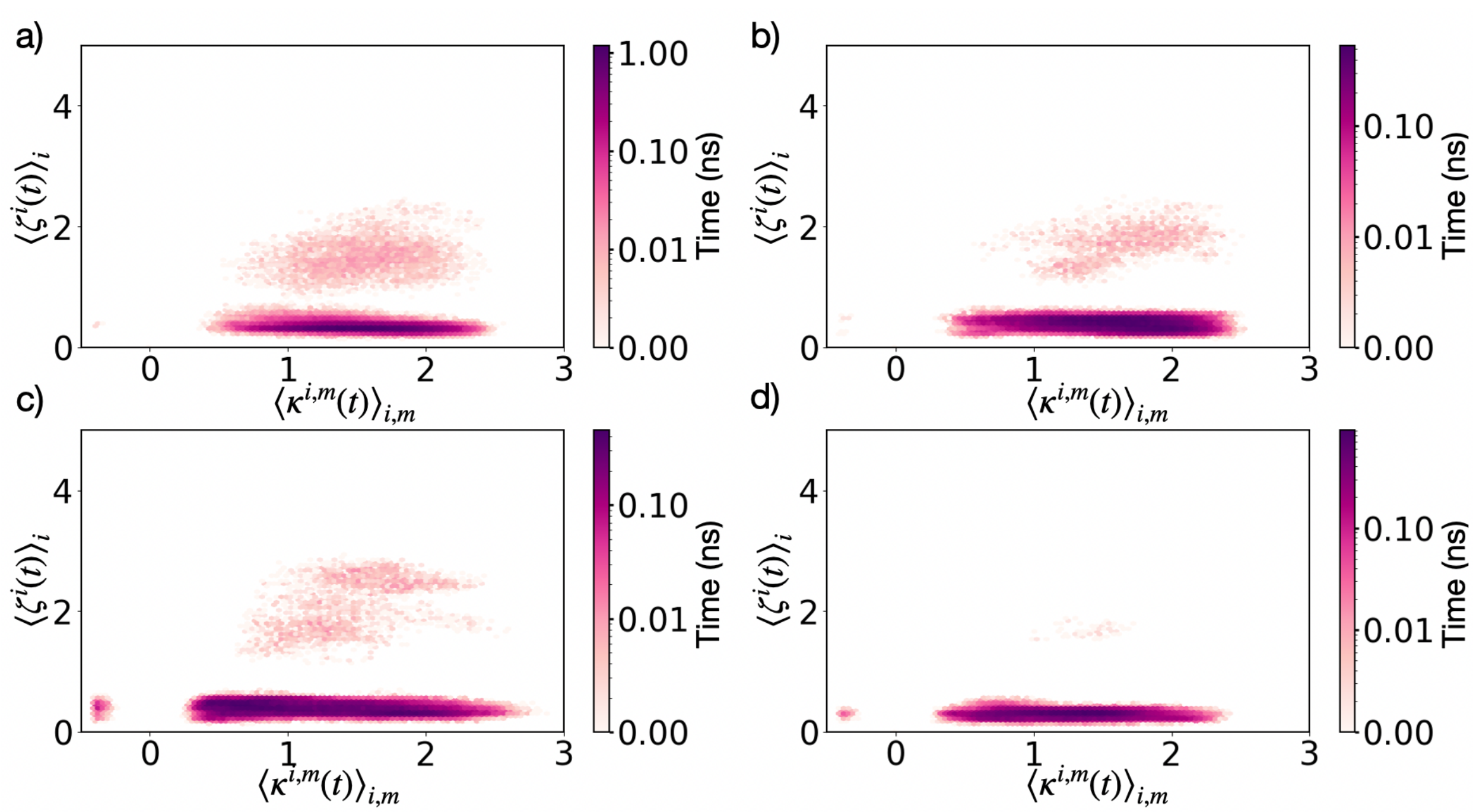

Figure S28: $\langle\kappa^{i,m}(t)\rangle_{i,m} - \langle\zeta^i(t)\rangle_i$ correlation for $(\text{PPG})_{12}$. a) 0 mM D-glucose. b) 500 mM D-glucose. c) 1000 mM D-glucose. d) 2000 mM D-glucose. The size of the second population decreases as 5.13%, 3.12%, 2.99%, 0.77% with D-glucose.

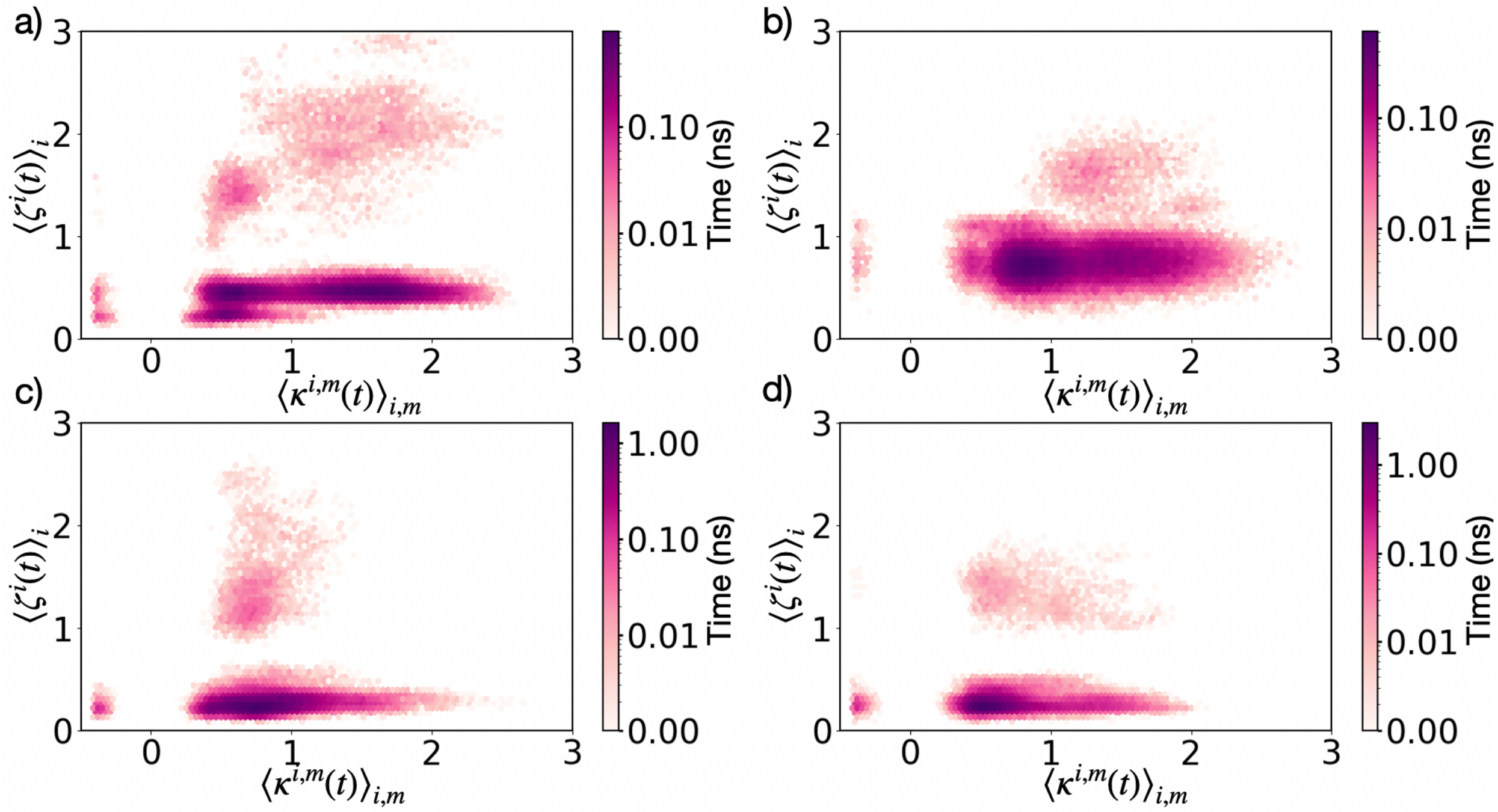

Figure S29: $\langle \kappa^{i,m}(t) \rangle_{i,m} - \langle \zeta^i(t) \rangle_i$ correlation for Gly$\xrightarrow{\text{mid}}$Ala. a) 0 mM D-glucose. b) 500 mM D-glucose. c) 1000 mM D-glucose. d) 2000 mM D-glucose. The size of the second population decreases as 18%, 12%, 5%, 3% with D-glucose.

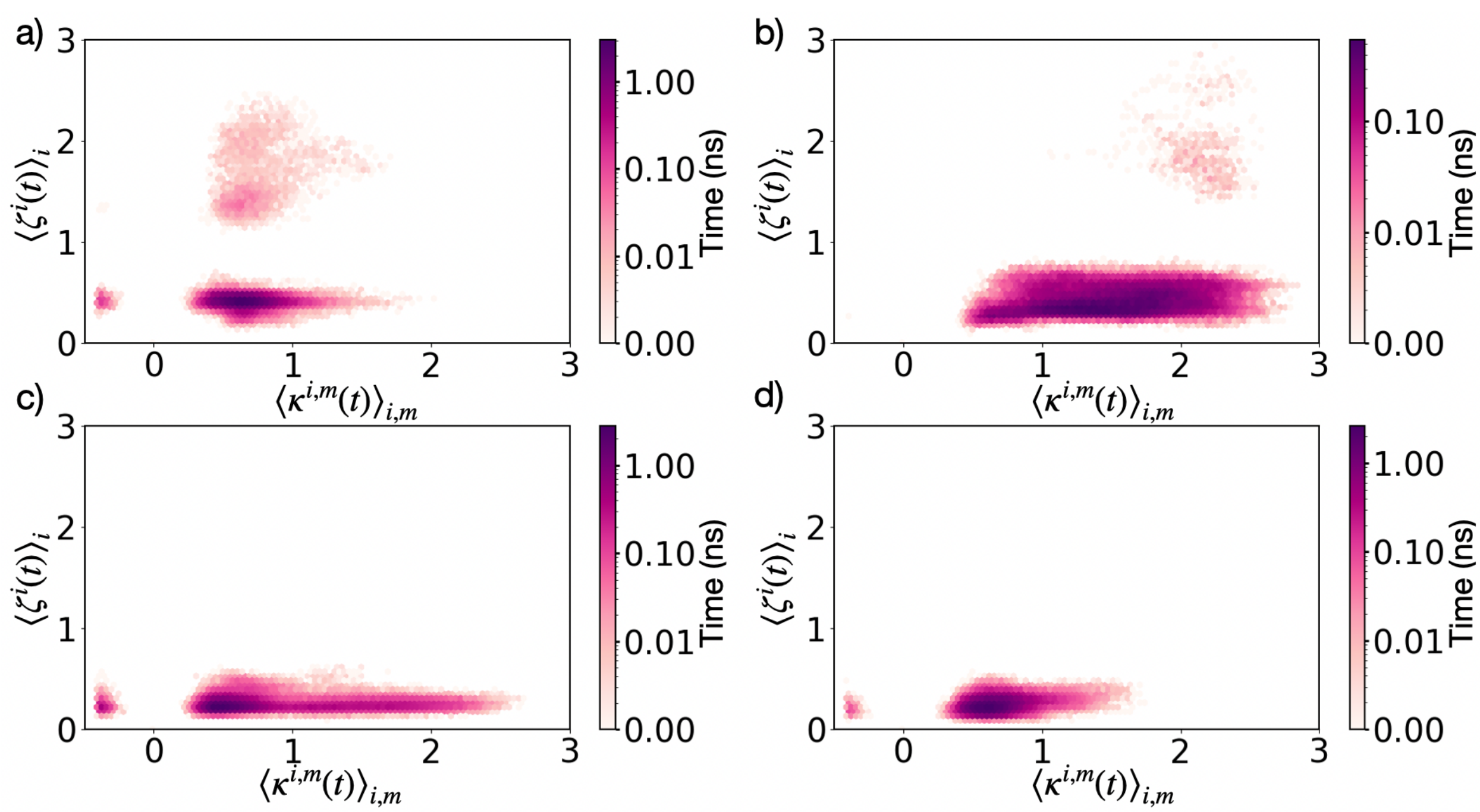

Figure S30: $\langle \kappa^{i,m}(t) \rangle_{i,m} - \langle \zeta^i(t) \rangle_i$ correlation for Gly$\xrightarrow{\text{end}}$Ala. a) 0 mM D-glucose. b) 500 mM D-glucose. c) 1000 mM D-glucose. d) 2000 mM D-glucose. The size of the second population decreases as 9%, 3%, 0%, 0% with D-glucose.

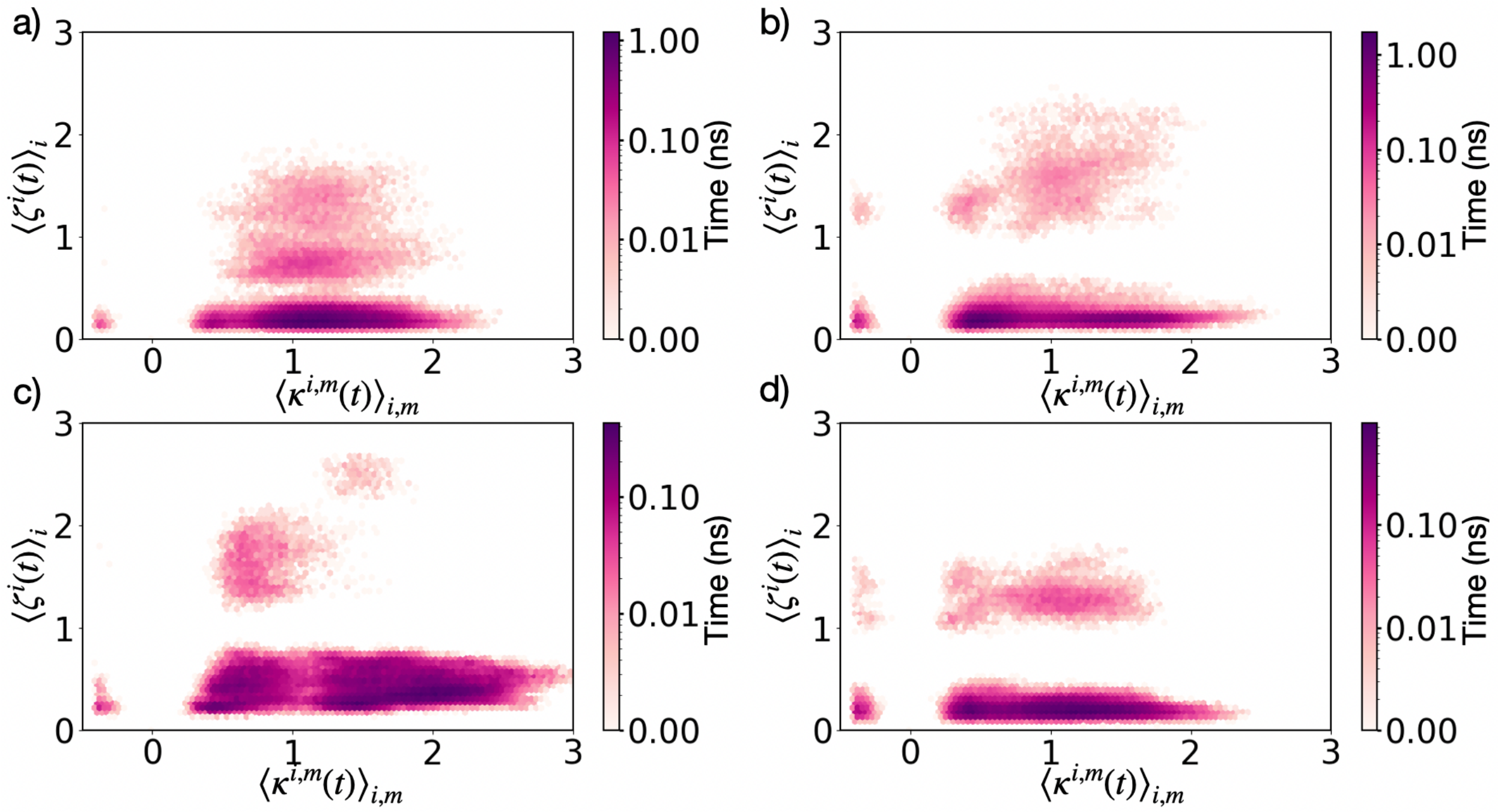

Figure S31: $\langle \kappa^{i,m}(t)\rangle_{i,m} - \langle \zeta^{i}(t)\rangle_{i}$ correlation for Pro$\xrightarrow{\text{end}}$Lys. a) 0 mM D-glucose. b) 500 mM D-glucose. c) 1000 mM D-glucose. d) 2000 mM D-glucose. The size of the second population decreases as 11%, 7%, 6%, 6% with D-glucose.

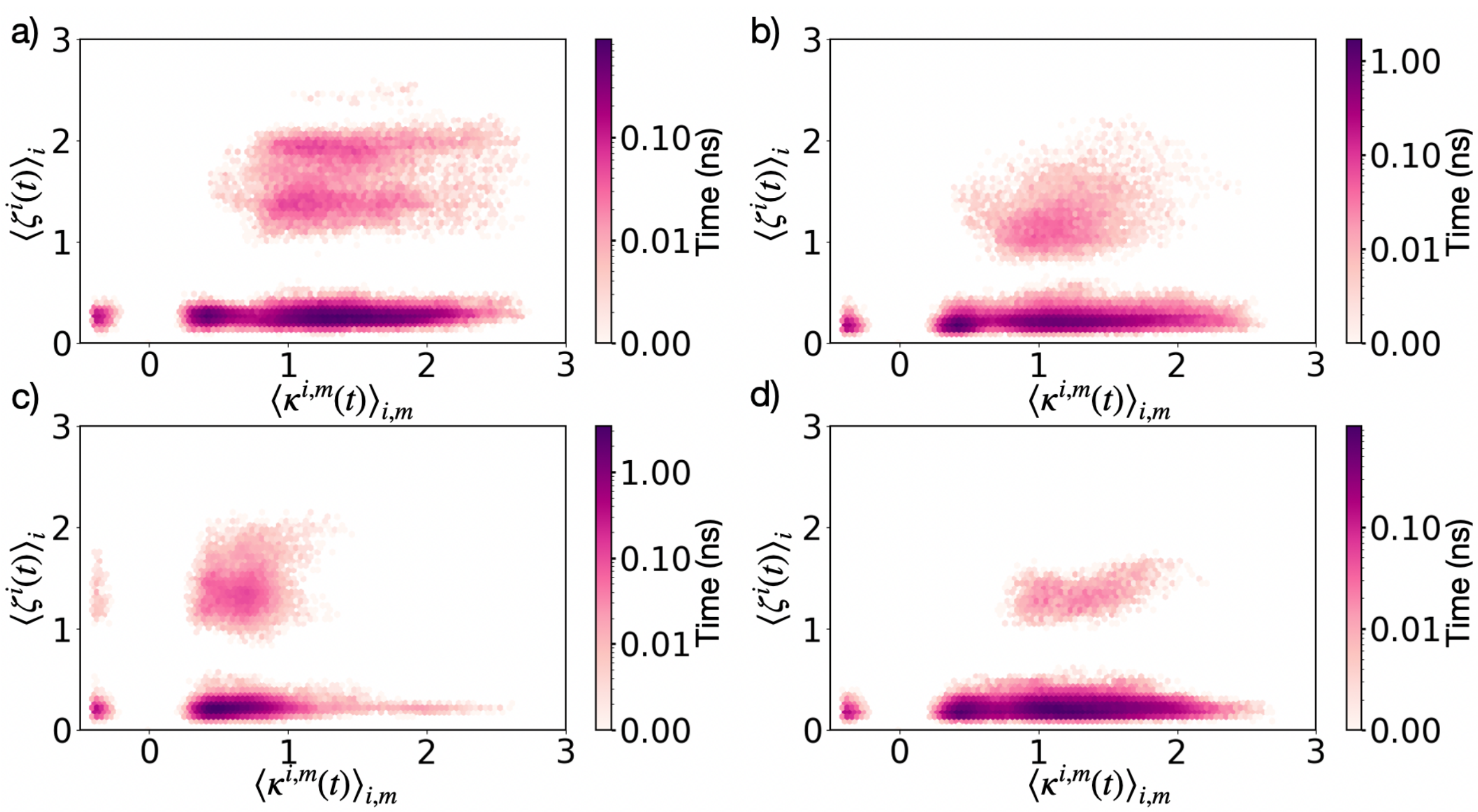

Figure S32: $\langle \kappa^{i,m}(t)\rangle_{i,m} - \langle \zeta^{i}(t)\rangle_{i}$ correlation for Pro$\xrightarrow{\text{mid}}$Lys. a) 0 mM D-glucose. b) 500 mM D-glucose. c) 1000 mM D-glucose. d) 2000 mM D-glucose. The size of the second population decreases as 15%, 12%, 8%, 3% with D-glucose.

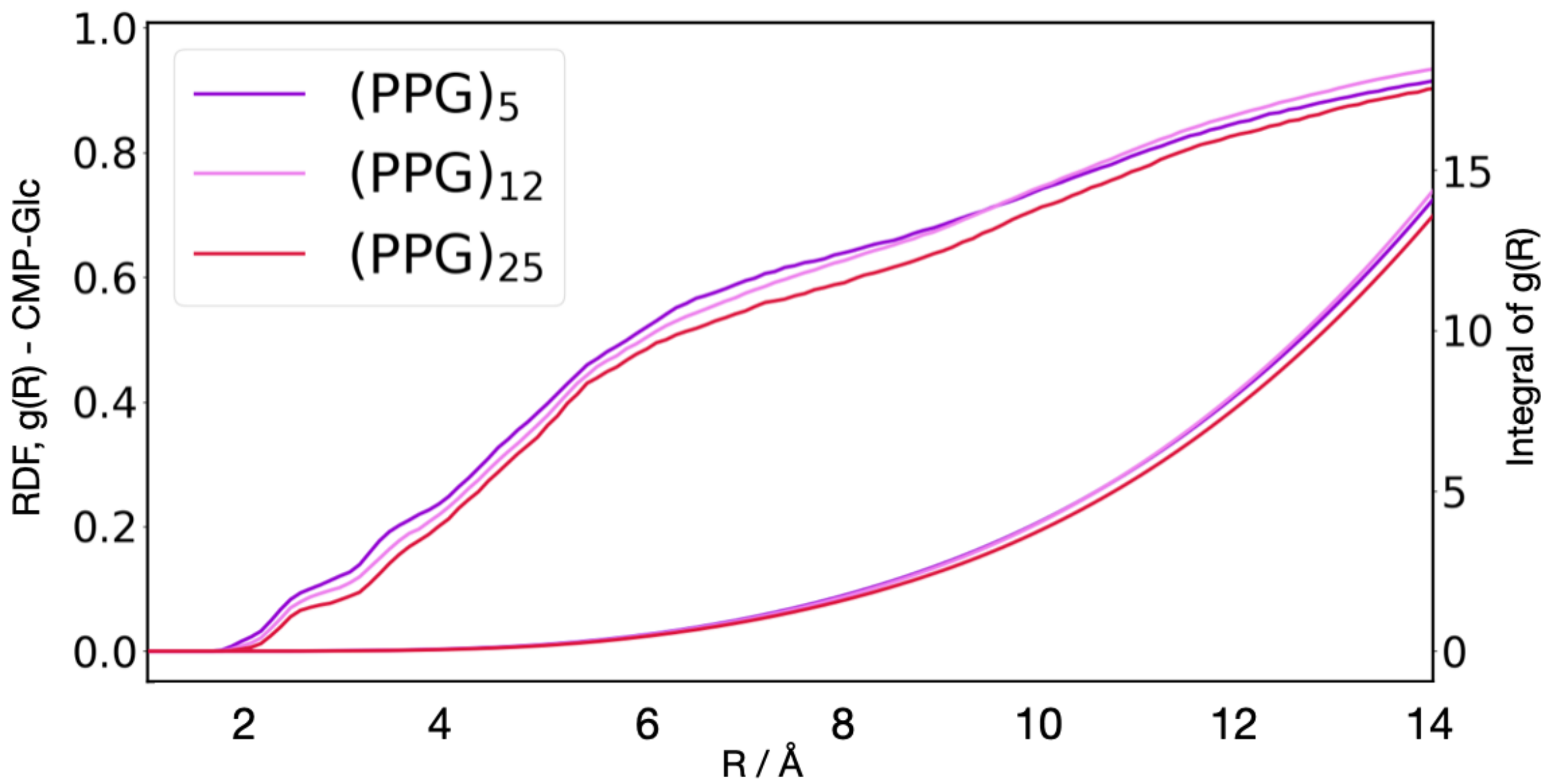

Figure S33: RDF of the $(PPG)_n$ CMPs with 2000 mM D-glucose, used to estimate proximity (5 Å).

**CMP-Glc system**		k_g^{ex} (ps^{-1})	max $L^g(e)$ (ps)
$(PPG)_{12}$	500 mM	0.24	1598
	1000 mM	0.48	2839
	2000 mM	1.24	4083
Gly$\xrightarrow{\text{end}}$Ala	500 mM	0.27	1389
	1000 mM	0.42	2116
	2000 mM	1.11	3784
Gly$\xrightarrow{\text{mid}}$Ala	500 mM	0.31	1711
	1000 mM	0.59	2447
	2000 mM	1.07	3998
Pro$\xrightarrow{\text{end}}$Lys	500 mM	0.15	1200
	1000 mM	0.46	4994
	2000 mM	0.83	4732
Pro$\xrightarrow{\text{mid}}$Lys	500 mM	0.19	1809
	1000 mM	0.45	3593
	2000 mM	0.79	5114
Pro$\xrightarrow{\text{end}}$Lys, Lys NH_3^+ only	500 mM	0.02	1189
	1000 mM	0.05	4971
	2000 mM	0.11	4693
Pro$\xrightarrow{\text{mid}}$Lys, Lys NH_3^+ only	500 mM	0.03	1802
	1000 mM	0.06	3583
	2000 mM	0.10	5089

Table S5: k_g^{ex} (ps^{-1}) and maximum $L^g(e)$ (longest residence event, ps) for D-glucose within 5 Å of the CMP and the Lys terminal NH_3^+ motif for $(PPG)_{12}$, Gly$\xrightarrow{\text{end}}$Ala, Gly$\xrightarrow{\text{mid}}$Ala, Pro$\xrightarrow{\text{end}}$Lys and Pro$\xrightarrow{\text{mid}}$Lys. Note that max $L^g(e)$ from the CMP and the Lys don't match exactly as the radial spheres don't overlap perfectly.

**D-glucose**	$\boldsymbol{D_{kk}^{\text{trans}}}$ **(10^{-5} cm^2/s)**	**(PPG)$_{12}$**	**Gly$\xrightarrow{\text{end}}$Ala**	**Gly$\xrightarrow{\text{mid}}$Ala**	**Pro$\xrightarrow{\text{end}}$Lys**	**Pro$\xrightarrow{\text{mid}}$Lys**
0 mM	$D_{\text{iso}}^{\text{trans}}$	1.2	1.3	1.3	1.3	1.3
	D_{xx}^{trans}	1.5	1.5	1.5	1.6	1.5
	D_{yy}^{trans}	1.2	1.3	1.3	1.3	1.3
	D_{zz}^{trans}	1.0	1.1	1.1	1.1	1.0
	$c_{\text{aniso}}^{\text{trans}}$	0.44	0.31	0.31	0.36	0.39
500 mM	$D_{\text{iso}}^{\text{trans}}$	1.4	1.4	1.4	1.4	1.4
	D_{xx}^{trans}	1.5	1.5	1.5	1.5	1.5
	D_{yy}^{trans}	1.4	1.3	1.4	1.4	1.4
	D_{zz}^{trans}	1.3	1.3	1.3	1.4	1.4
	$c_{\text{aniso}}^{\text{trans}}$	0.14	0.14	0.14	0.09	0.08
1000 mM	$D_{\text{iso}}^{\text{trans}}$	1.4	1.3	1.3	1.3	1.4
	D_{xx}^{trans}	1.5	1.5	1.5	1.5	1.5
	D_{yy}^{trans}	1.4	1.3	1.3	1.3	1.4
	D_{zz}^{trans}	1.2	1.1	1.1	1.2	1.2
	$c_{\text{aniso}}^{\text{trans}}$	0.22	0.31	0.31	0.23	0.24
2000 mM	$D_{\text{iso}}^{\text{trans}}$	1.5	1.4	1.4	1.4	1.3
	D_{xx}^{trans}	1.6	1.5	1.5	1.5	1.4
	D_{yy}^{trans}	1.6	1.4	1.4	1.4	1.4
	D_{zz}^{trans}	1.2	1.3	1.3	1.2	1.1
	$c_{\text{aniso}}^{\text{trans}}$	0.27	0.14	0.14	0.25	0.24

**D-glucose**	$\boldsymbol{D_{kk}^{\text{rot}}}$ **(rad^2/s)**	**(PPG)$_{12}$**	**Gly$\xrightarrow{\text{end}}$Ala**	**Gly$\xrightarrow{\text{mid}}$Ala**	**Pro$\xrightarrow{\text{end}}$Lys**	**Pro$\xrightarrow{\text{mid}}$Lys**
0 mM	$D_{\text{iso}}^{\text{rot}}$	1.2	1.3	1.2	1.6	1.2
	$D_{r_1r_1}^{\text{rot}}$	1.4	1.3	1.3	1.9	1.4
	$D_{r_2r_2}^{\text{rot}}$	1.3	1.3	1.3	1.5	1.2
	$D_{r_3r_3}^{\text{rot}}$	1.0	1.2	0.9	1.4	1.0
	$c_{\text{aniso}}^{\text{rot}}$	0.32	0.08	0.31	0.31	0.32
500 mM	$D_{\text{iso}}^{\text{rot}}$	1.3	1.3	1.2	1.4	1.4
	$D_{r_1r_1}^{\text{rot}}$	1.5	1.5	1.5	1.5	1.6
	$D_{r_2r_2}^{\text{rot}}$	1.3	1.2	1.2	1.4	1.4
	$D_{r_3r_3}^{\text{rot}}$	1.0	1.1	1.0	1.3	1.3
	$c_{\text{aniso}}^{\text{rot}}$	0.39	0.31	0.33	0.17	0.19
1000 mM	$D_{\text{iso}}^{\text{rot}}$	1.4	1.3	1.2	1.4	1.4
	$D_{r_1r_1}^{\text{rot}}$	1.6	1.5	1.5	1.6	1.7
	$D_{r_2r_2}^{\text{rot}}$	1.5	1.2	1.2	1.5	1.3
	$D_{r_3r_3}^{\text{rot}}$	1.1	1.1	1.0	1.1	1.2
	$c_{\text{aniso}}^{\text{rot}}$	0.36	0.31	0.42	0.33	0.31
2000 mM	$D_{\text{iso}}^{\text{rot}}$	1.4	1.4	1.4	1.1	1.3
	$D_{r_1r_1}^{\text{rot}}$	1.6	1.5	1.5	1.3	1.6
	$D_{r_2r_2}^{\text{rot}}$	1.5	1.4	1.5	1.1	1.2
	$D_{r_3r_3}^{\text{rot}}$	1.2	1.2	1.1	0.9	1.1
	$c_{\text{aniso}}^{\text{rot}}$	0.28	0.21	0.29	0.35	0.34

Table S6: Diagonal elements of $\mathbf{D}^{\text{trans}}$ and $\mathbf{D}^{\text{rot}}$ for (PPG)$_{12}$ and the singly-mutated CMPs in D-glucose, along with $D_{\text{iso}}^{\text{trans}}$, $D_{\text{iso}}^{\text{rot}}$, $c_{\text{aniso}}^{\text{trans}}$ and $c_{\text{aniso}}^{\text{rot}}$. The error of the mean (err) is calculated using the standard deviation divided by $\sqrt{5}$, where 5 corresponds to 5 non-overlapping trajectory segments of 20 ns each. $0.1 \leq err \leq 0.3$ for all elements of $\mathbf{D}^{\text{trans}}$ and $\mathbf{D}^{\text{rot}}$.

References

[1] Stanley W Shepperd. Quaternion from rotation matrix. *Journal of guidance and control*, 1(3):223–224, 1978.

[2] Soheil Sarabandi and Federico Thomas. A survey on the computation of quaternions from rotation matrices. *Journal of mechanisms and robotics*, 11(2):021006, 2019.

[3] Jan K Rainey and M Cynthia Goh. An interactive triple-helical collagen builder. *Bioinformatics*, 20(15): 2458–2459, 2004.

[4] Rita Berisio, Luigi Vitagliano, Lelio Mazzarella, and Adriana Zagari. Crystal structure of the collagen triple helix model [(pro-pro-gly) 10] 3. *Protein science*, 11(2):262–270, 2002.

[5] Joshua A Rackers, Zhi Wang, Chao Lu, Marie L Laury, Louis Lagardère, Michael J Schnieders, Jean-Philip Piquemal, Pengyu Ren, and Jay W Ponder. Tinker 8: software tools for molecular design. *Journal of chemical theory and computation*, 14(10):5273–5289, 2018.

[6] W Ying Chow, Dominique Bihan, Chris J Forman, David A Slatter, David G Reid, David J Wales, Richard W Farndale, and Melinda J Duer. Hydroxyproline ring pucker causes frustration of helix parameters in the collagen triple helix. *Scientific reports*, 5(1):12556, 2015.

[7] Jürgen Engel and Hans Peter Bächinger. Structure, stability and folding of the collagen triple helix. *Collagen: primer in structure, processing and assembly*, pages 7–33, 2005.

[8] Roberto Improta, Franca Mele, Orlando Crescenzi, Caterina Benzi, and Vincenzo Barone. Understanding the role of stereoelectronic effects in determining collagen stability. 2. a quantum mechanical/molecular mechanical study of (proline-proline-glycine) n polypeptides. *Journal of the American Chemical Society*, 124(26):7857–7865, 2002.

[9] Sanghyun Park, Randall J Radmer, Teri E Klein, and Vijay S Pande. A new set of molecular mechanics parameters for hydroxyproline and its use in molecular dynamics simulations of collagen-like peptides. *Journal of computational chemistry*, 26(15):1612–1616, 2005.

[10] V Nagarajan, Shigehiro Kamitori, and Kenji Okuyama. Crystal structure analysis of collagen model peptide (pro-pro-gly) 10. *The journal of biochemistry*, 124(6):1117–1123, 1998.
